# A novel dataset for nuclei and tissue segmentation in melanoma with baseline nuclei segmentation and tissue segmentation benchmarks

**DOI:** 10.1093/gigascience/giaf011

**Published:** 2025-02-19

**Authors:** Mark Schuiveling, Hong Liu, Daniel Eek, Gerben E Breimer, Karijn P M Suijkerbuijk, Willeke A M Blokx, Mitko Veta

**Affiliations:** Department of Medical Oncology, University Medical Center Utrecht, Utrecht University, 3584 CG Utrecht, the Netherlands; Medical Image Analysis, Department of Biomedical Engineering, Eindhoven University of Technology, 5600 MB Eindhoven, the Netherlands; Medical Image Analysis, Department of Biomedical Engineering, Eindhoven University of Technology, 5600 MB Eindhoven, the Netherlands; Department of Pathology, University Medical Center Utrecht, Utrecht University, 3584 CG Utrecht, the Netherlands; Department of Medical Oncology, University Medical Center Utrecht, Utrecht University, 3584 CG Utrecht, the Netherlands; Department of Pathology, University Medical Center Utrecht, Utrecht University, 3584 CG Utrecht, the Netherlands; Medical Image Analysis, Department of Biomedical Engineering, Eindhoven University of Technology, 5600 MB Eindhoven, the Netherlands

**Keywords:** Melanoma, H&E-stained histopathology, nuclei segmentation, tissue segmentation, TILs

## Abstract

**Background:**

Melanoma is an aggressive form of skin cancer in which tumor-infiltrating lymphocytes (TILs) are a biomarker for recurrence and treatment response. Manual TIL assessment is prone to interobserver variability, and current deep learning models are not publicly accessible or have low performance. Deep learning models, however, have the potential of consistent spatial evaluation of TILs and other immune cell subsets with the potential of improved prognostic and predictive value. To make the development of these models possible, we created the Panoptic Segmentation of nUclei and tissue in advanced MelanomA (PUMA) dataset and assessed the performance of several state-of-the-art deep learning models. In addition, we show how to improve model performance further by using heuristic postprocessing in which nuclei classes are updated based on their tissue localization.

**Results:**

The PUMA dataset includes 155 primary and 155 metastatic melanoma hematoxylin and eosin–stained regions of interest with nuclei and tissue annotations from a single melanoma referral institution. The Hover-NeXt model, trained on the PUMA dataset, demonstrated the best performance for lymphocyte detection, approaching human interobserver agreement. In addition, heuristic postprocessing of deep learning models improved the detection of noncommon classes, such as epithelial nuclei.

**Conclusion:**

The PUMA dataset is the first melanoma-specific dataset that can be used to develop melanoma-specific nuclei and tissue segmentation models. These models can, in turn, be used for prognostic and predictive biomarker development. Incorporating tissue and nuclei segmentation is a step toward improved deep learning nuclei segmentation performance. To support the development of these models, this dataset is used in the PUMA challenge.

## Background

Melanoma is an aggressive form of skin cancer with increasing incidence [1]. Primary melanoma is treated with surgical excision, whereas advanced, metastasized melanoma is most commonly treated with immune checkpoint inhibition therapy, a form of cancer immunotherapy. However, half of the patients with advanced melanoma do not respond to this treatment, which is costly and potentially toxic [2–5].

Previous studies showed that tumor infiltrating lymphocytes (TILs) in hematoxylin and eosin (H&E)–stained slides of metastatic melanoma before the start of treatment are associated with a higher chance of response to immune checkpoint inhibition and an increase in survival [6, 7]. In addition, TILs are associated with reduced recurrence rates in primary melanoma [8]. Therefore, assessment of TILs can serve as a prognostic biomarker in melanoma treatment.

Currently, TILs are scored manually, either by estimating a stromal percentage or through using a multitier system like the Clark score, which categorizes TILs as absent, nonbrisk, or brisk [6, 9, 10]. However, substantial interobserver variability exists among pathologists [6, 11, 12]. A deep learning–based assessment could result in a more precise, consistent, and fine-grained assessment of TILs.

**Figure 1:**
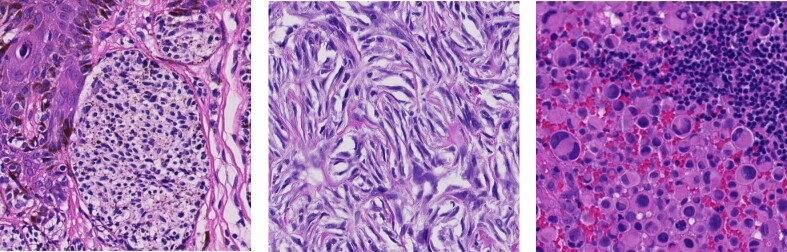
Examples of melanoma histopathology appearances: a lymphocyte-like morphology on the left, a stromal growth pattern in the center, and a tumor with large, variable nuclei on the right.

To date, 2 deep learning–based models have been used to evaluate TILs in melanoma histopathology. The first model, NN192, uses watershed segmentation followed by a fully connected neural network [7]. However, this older, suboptimal technique results in lower performance [13]. The second model, LUNIT’s Scope IO pan-tumor model, is not melanoma specific and not publicly available [14, 15]. Publicly available models, such as Hover-Net pretrained on the PanNuke dataset (which includes skin samples), can be used to detect TILs in melanoma but have suboptimal performance. This is due to the model not being melanoma-specific [16, 17]. As a result, misclassifications occur because melanoma cells can resemble other cell types, such as stroma or lymphocytes (Fig. 1) [13, 18, 19].

As for datasets that can be used to train deep learning models usable in melanoma, the NulnsSeg [20] and CryoNuSeg [21] datasets also partly consist of skin samples. However, these datasets do not have separate classes for nuclei such as tumors or lymphocytes, rendering them less useful for creating deep learning nuclei classification algorithms. In contrast, the SegPath dataset does classify nuclei as lymphocytes using a ground truth based on immunofluorescence staining. However, it contains only 1 melanoma sample with CD3/CD20 annotations [22].

Next to the presence of (deep learning–detected) TILs, the localization of TILs is of importance. In breast cancer and non–small cell lung cancer, TILs located within the tumor or intratumoral stroma regions, rather than in necrotic tissue, are predictive of outcomes [23, 24]. No public dataset with tissue annotations or a public model capable of segmenting melanoma tumor and necrotic tissue regions exists at this moment.

Furthermore, other immune cell subsets might also have prognostic implications. For example, neutrophils are associated with an increased chance of primary melanoma metastasizing, and B-cell presence is associated with response to immune checkpoint inhibition therapy in melanoma [25, 26]. The 2020 MoNuSAC challenge showed that it is possible to segment immune cell subsets in H&E-stained histopathology images [27].

Consequently, there is a need for a deep learning model capable of segmenting nuclei of tumor cells and different immune cell subsets in H&E slides of melanoma. In addition, such a model should be capable of segmenting tissue areas that can be used for nuclei localization. To address these needs, we created the Panoptic Segmentation of nUclei and tissue in advanced MelanomA (PUMA) dataset.

In this article, we describe the methodology used to create the dataset. Furthermore, we provide nuclei instance segmentation and tissue semantic segmentation benchmarks as well as a first step in improving nuclei segmentation due to the integration of a tissue and nuclei segmentation.

## Data Description

The PUMA dataset consists of regions of interest (ROIs) with nuclei and tissue annotations. ROIs originate from H&E-stained histological slides of melanoma specimens. The dataset’s goal is to facilitate the development of deep learning models capable of segmenting nuclei and tissue. To stimulate the use of the dataset and create novel deep learning models, the dataset will also be used for a medical image analysis challenge hosted on the grand-challenge.org platform (accessible through the PUMA Grand Challenge website [28]). The models created with the dataset and the PUMA challenge, in turn, can be used for prognostic biomarker generation in melanoma treatment.

The dataset consists of 155 primary and 155 metastatic melanoma manually selected ROIs, scanned at 40× magnification (0.23 µm/px) with a resolution of 1,024 × 1,024 pixels. For these ROIs, annotations of both tissue and nuclei are supplied, as well as a context ROI of 5,120 × 5,120 pixels centered on the ROI. Annotations were created by a medical expert (author M.S.) and checked and corrected by a dermatopathologist (author W.B.). All cases were digitized in a large melanoma referral center, but 76 cases are revisions or consultations originating from other treatment hospitals. Annotations are in the .GeoJSON format, making annotations easily visualizable with the open-source pathology image viewer QuPath [29, 30].

From the total dataset of 310 ROIs, a training set consisting of 103 primary and 103 metastatic ROIs has been made publicly available [31]. The remaining 104 ROIs are not publicly available as they are used in the PUMA challenge. In this challenge, 10 ROIs are used a preliminary test set for testing of functioning of models. The remaining 94 samples are used as an independent test set for final metric calculations. To be able to compare results from the PUMA challenge to our article, results in this study are shown for this independent test set.

**Figure 2:**
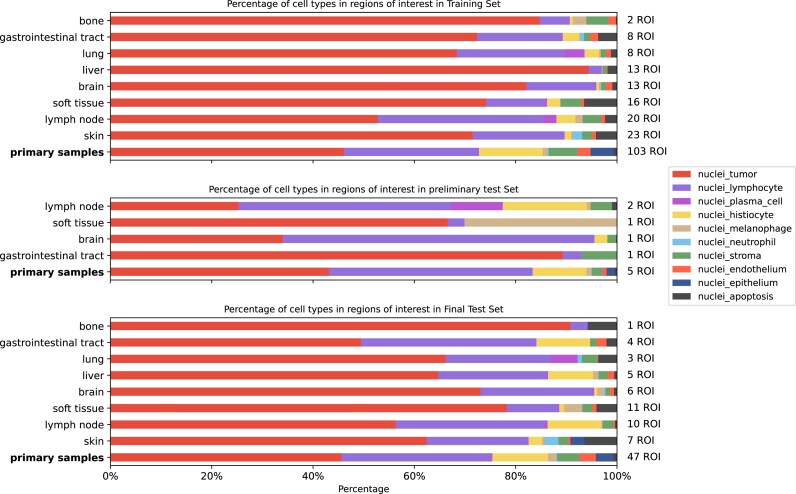
Distribution of nuclei and sampled tissue in the PUMA dataset for primary melanoma regions of interest and metastatic melanoma regions of interest stratified according to metastasis location. The primary samples are depicted as the last row of each figure graph.

The public set consists of 97,429 nuclei in 103 primary melanoma ROIs and 103 metastatic melanoma ROIs. The preliminary test set consists of 5 primary and 5 metastatic melanoma ROIs with a total of 4,860 nuclei. The final test set, consisting of the remaining 47 primary and 47 metastatic samples, contains 45,406 nuclei. The distribution of nuclei types and metastatic sample location is visualized in Fig. 2.

As we published previously, more lymphocytes are present in primary samples than in metastatic samples [6]. The most common metastatic lesion sites are lymph nodes and skin metastases. In all samples, lymphocytes and tumor nuclei form the majority of nuclei.

**Figure 3:**
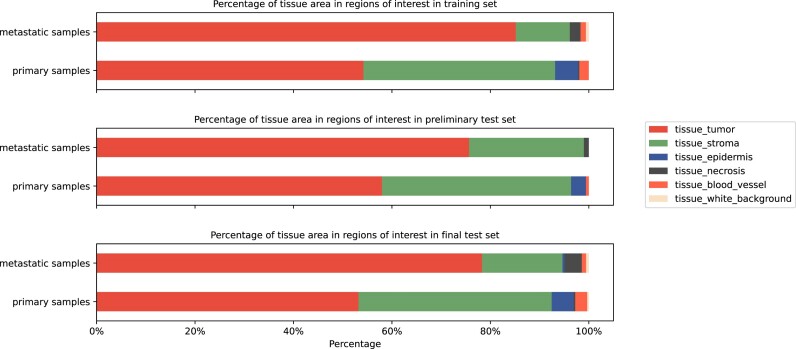
Distribution of tissue class area in the PUMA dataset.

The tissue distribution for the training, preliminary, and final test sets is visualized in Fig. 3. In primary samples, more tumoral stroma is present compared to metastatic samples. The epidermis and necrotic area are underrepresented in both datasets.

A detailed description of the dataset creation process can be found in the Methods section.

### Analyses

To set a baseline for the PUMA dataset, we performed 4 experiments. The first experiment was semantic segmentation of the tumor, stroma, epidermis, blood vessel, and necrotic tissue classes. The second experiment was nuclei segmentation with 3 nuclei classes: tumor, lymphocyte, and other. For the third experiment, we performed nuclei segmentation with all nuclei classes: tumor, lymphocyte, plasma cell, histiocyte, melanophage, neutrophil, stroma, endothelium, epithelium, and apoptosis. Finally, we show an incorporation of tissue predictions to update the nuclei predictions as a form of heuristic postprocessing.

We used 5-fold cross-validation while training and report the results of inference on the 94 ROIs of the final independent test set used in the PUMA challenge. In addition, we report the results of the inter- and intraobserver agreement as performed on 12 random samples.

### Tissue segmentation

In the first experiment, semantic segmentation of tissue was performed with a nnU-Net model and a Mask2Former model with the backbone replaced by the UNI pathology foundation model [32–34]. The goal of this analysis was to assess to what extent semantic segmentation of different tissue classes is possible with state-of-the-art segmentation models and to evaluate how this correlates with intra- and interobserver agreement. For evaluation of segmentation, the Dice score was computed for each class per sample and averaged across all samples (referred to as average Dice). Additionally, a Dice score was calculated per class on a concatenated sample. To create this sample, all images were combined along 1 axis, resulting in a single large image with the width of 1 image and a length equal to the number of images × the height of 1 image. This is referred to as the micro Dice.

**Figure 4:**
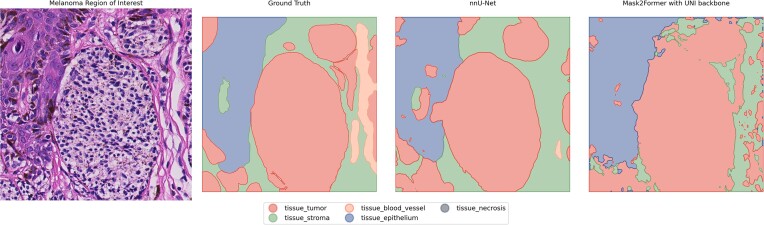
The visual result of semantic segmentation of tissue with the ground truth, the result of the nnU-Net model, and the Mask2Former model.

Dice scores are visualized in Table 1, and a visualization of the segmentation is presented in Fig. 4. The nnU-Net model achieved the highest overall Dice scores but could not recognize necrosis in the dataset. While Mask2Former Dice scores were lower, it could detect a small part of the necrosis area. When compared to the Dice score of intra- and interobserver agreement, the Dice scores for both models were low in the stroma, epidermis, blood vessel, and necrosis classes.

**Table 1:** Average and micro-average Dice scores of semantic segmentation of tissue over all samples. Results are displayed for a nnU-Net model and a Mask2Former model with the backbone replaced by the UNI pathology foundation model. For comparison, DICE scores of intra- and interobsever agreement are displayed.

	Class	Tumor [95% CI]	Stroma [95% CI]	Epidermis [95% CI]	Blood vessel [95% CI]	Necrosis [95% CI]	Average [95% CI]
**nnU-Net**	Average	0.87 [0.83–0.90]	0.59 [0.52–0.68]	0.91 [0.86–0.96]	0.54 [0.45–0.63]	0.93 [0.87–0.98]	0.77 [0.73–0.80]
	Micro-Average	0.91 [0.88–0.94]	0.78 [0.71–0.84]	0.69 [0.42–0.86]	0.35 [0.23–0.47]	0.01 [0.00–0.04]	0.55 [0.54–0.55]
**Mask2-Former (UNI backbone)**	Average	0.81 [0.76–0.86]	0.47 [0.39–0.55]	0.90 [0.85–0.96]	0.46 [0.36–0.57]	0.92 [0.86–0.97]	0.71 [0.68–0.75]
	Micro-Average	0.86 [0.82–0.89]	0.62 [0.52–0.71]	0.63 [0.34–0.84]	0.01 [0.00–0.02]	0.09 [0.00–0.23]	0.44 [0.43–0.44]
**Intraobserver**	Average	0.96 [0.93–0.99]	0.89 [0.72–0.99]	1.00 [0.99–1.00]	0.86 [0.75–0.95]	0.97 [0.92–1.00]	0.94 [0.90–0.97]
	Micro-Average	0.98 [0.95–0.99]	0.94 [0.90–0.97]	0.98 [0.98–1.00]	0.68 [0.52–0.79]	0.91 [0.70–1.00]	0.90 [0.90–0.91]
**Interobserver**	Average	0.97 [0.95–0.98]	0.89 [0.73–0.99]	1.00 [0.99–1.00]	0.72 [0.50–0.90]	0.97 [0.91–1.00]	0.91 [0.87–0.95]
	Micro-Average	0.98 [0.97–0.99]	0.94 [0.89–0.97]	0.97 [0.96–1.00]	0.73 [0.59–0.83]	0.90 [0.67–1.00]	0.90 [0.90–0.90]

### Segmentation of 3 nuclei classes

In the second experiment, nuclei segmentation was performed for 3 classes: tumor nuclei, lymphocytes (including plasma nuclei), and other. The goal of the analysis was to evaluate the usability of existing models in skin and/or melanoma histopathology, compare them with models trained on our dataset, and assess how this correlates with intra- and interobserver agreement. For this experiment, we compared the NN192 model, Hover-Net and Hover-NeXt trained on the PanNuke dataset (a pan-tissue dataset that includes skin tissue), and Hover-Net and Hover-NeXt trained on our dataset. Among all evaluated models, the Hover-NeXt model trained on the PUMA dataset, using 3 classes as input for training, demonstrated the highest performance, with a F_1_ score for lymphocytes close to the intra- and interobserver agreement.

**Figure 5:**
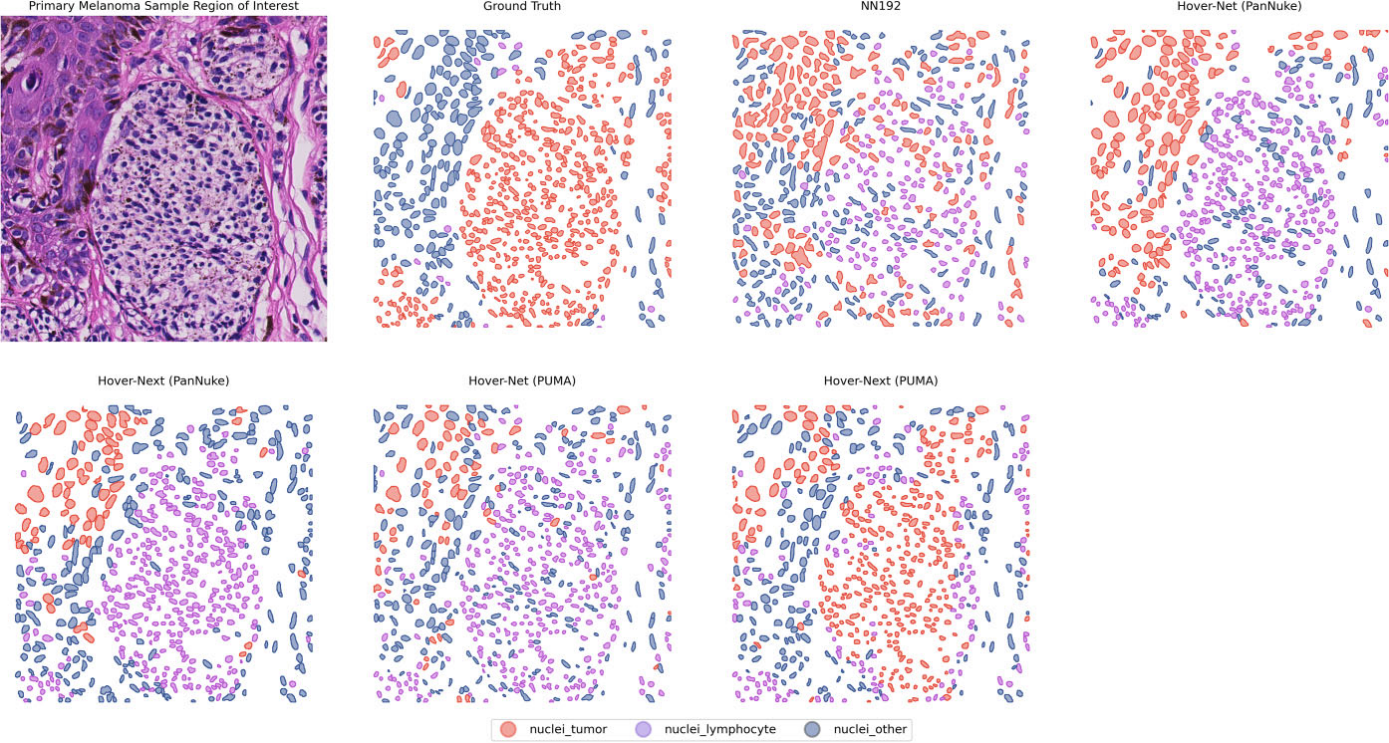
Visual results of segmentation of tumor nuclei, lymphocytes, and other nuclei. Results are shown for the NN192 model, the Hover-Net and Hover-NeXt model trained on the PanNuke dataset, and the Hover-Net and Hover-NeXt model trained on the PUMA dataset.

The NN192 model, a melanoma-specific model trained to recognize lymphocytes, showed the lowest performance. The Hover-Net and Hover-NeXt models trained on the PanNuke dataset were more successful in detecting lymphocytes. However, the PanNuke-trained Hover-Net model tended to misclassify tumor nuclei as lymphocytes. Models trained on the PUMA dataset, logically, had better performance on evaluation of the test dataset. Results are displayed in Table 2, and an example of the model predictions is shown in Fig. 5.

**Table 2:** F_1_ scores for segmentation of tumor, lymphocyte, and other nuclei categories. Results are shown for intra- and interobserver agreement, the NN192 model, Hover-Net and Hover-NeXt trained on the PanNuke dataset, and Hover-Net and Hover-NeXt trained on the PUMA dataset.

	Tumor [95% CI]	Lymphocyte [95% CI]	Other [95% CI]	Micro F_1_ [95% CI]	Average F_1_ [95% CI]
**Intraobserver agreement**	0.92 [0.90–0.94]	0.85 [0.80–0.89]	0.82 [0.77–0.86]	0.89 [0.89–0.89]	0.86 [0.85–0.87]
**Interobserver agreement**	0.89 [0.87–0.91]	0.83 [0.79–0.88]	0.76 [0.71–0.81]	0.85 [0.85–0.85]	0.83 [0.82–0.84]
**NN192**	0.59 [0.59–0.60]	0.11 [0.10–0.11]	0.13 [0.12–0.14]	0.46 [0.46–0.46]	0.28 [0.27–0.28]
**Hover-Net (PanNuke)**	0.74 [0.74–0.75]	0.57 [0.55–0.58]	0.37 [0.36–0.38]	0.64 [0.64–0.64]	0.56 [0.56–0.56]
**Hover-NeXt (PanNuke)**	0.68 [0.67–0.68]	0.68 [0.67–0.70]	0.38 [0.37–0.39]	0.61 [0.61–0.61]	0.58 [0.58–0.58]
**Hover-Net (PUMA)**	0.74 [0.73–0.74]	0.69 [0.68–0.69]	0.41 [0.41–0.42]	0.66 [0.66–0.66]	0.61 [0.61–0.61]
**Hover-NeXt (PUMA)**	0.81 [0.81–0.81]	0.80 [0.79–0.81]	0.50 [0.49–0.51]	0.76 [0.76–0.76]	0.70 [0.70–0.71]

### Classification of all nuclei classes

In the third experiment, segmentation was performed for all nuclei classes within the PUMA dataset using Hover-Net and Hover-NeXt. In this analysis, we evaluated to what extent Hover-Net and Hover-NeXt are able to correctly segment all nuclei classes present in the dataset. In addition, this analysis aimed at establishing intra- and interobserver agreement for all classes.

**Figure 6:**
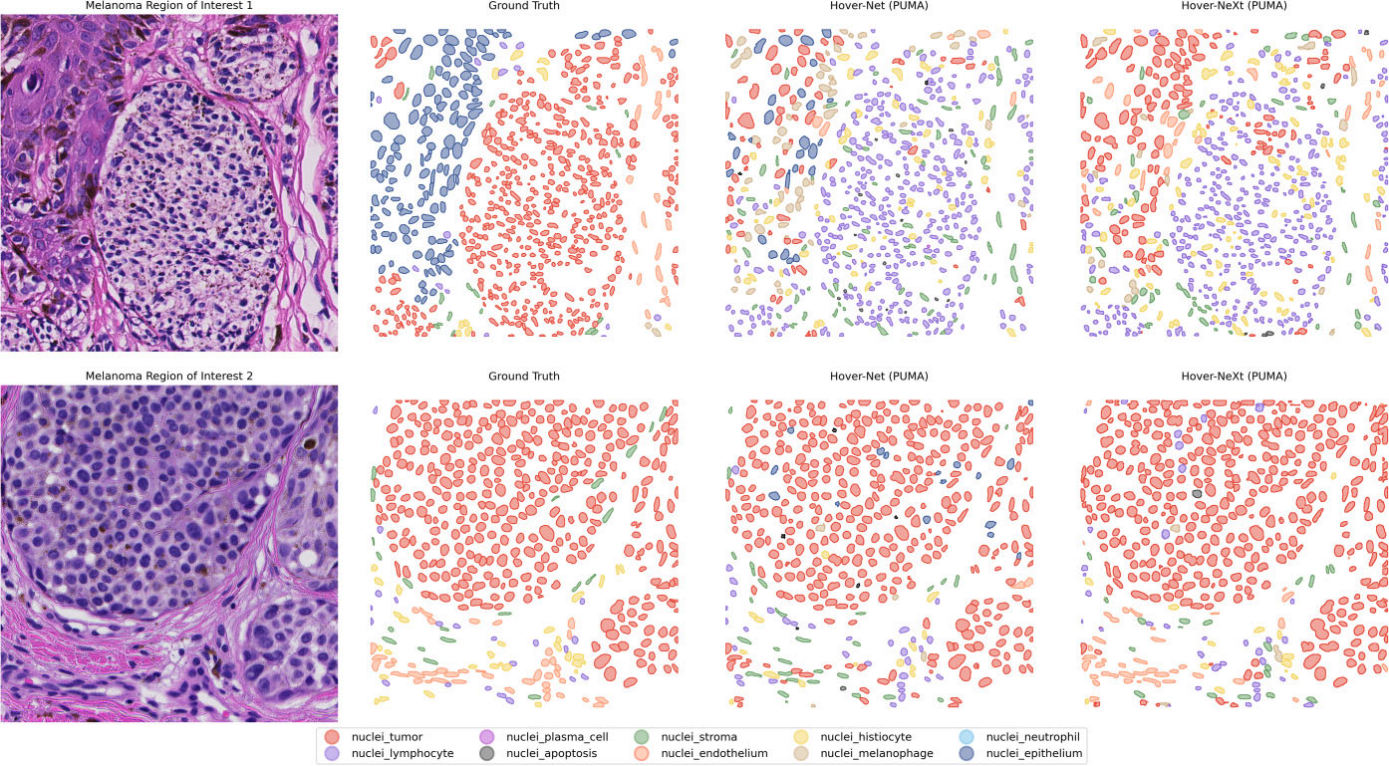
Visual results of segmentation of all nuclei classes. Results are shown for the Hover-Net and Hover-NeXt model trained on the PUMA dataset. In ROI 1, neither of the models is able to classify tumor nuclei and lymphocytes. In ROI 2, model performance is better with identification of tumor nuclei and endothelium nuclei.

Both Hover-Net and Hover-Next showed low performance in terms of segmentation of all noncommon classes (Table 3). In addition, the F_1_ scores for tumors and lymphocytes were lower when compared to the same models trained with 3 nuclei classes. In the visual representation (Fig. 6), the decreased capacity of the Hover-NeXt model of segmenting difficult cases is clearly visible.

**Table 3:** F_1_ scores for intra- and interobserver agreement and model segmentation in all classes

	Tumor	Lymphocyte	Plasma cell	Histiocyte	Melanophage	Neutrophil
**Intraobserver**	0.92 [0.90–0.94]	0.85 [0.80–0.89]	0.0 [0.0–0.0]	0.60 [0.53–0.67]	0.59 [0.26–0.97]	0.77 [0.61–0.93]
**Interobserver**	0.89 [0.87–0.91]	0.83 [0.79–0.88]	0.0 [0.0–0.0]	0.44 [0.37–0.51]	0.28 [0.00–0.64]	0.68 [0.50–0.85]
**Hover-Net (PUMA)**	0.73 [0.72–0.73]	0.67 [0.66–0.67]	0.07 [0.06–0.09]	0.27 [0.26–0.27]	0.31 [0.29–0.33]	0.15 [0.13–0.18]
**Hover-NeXt (PUMA)**	0.70 [0.70–0.71]	0.71 [0.70–0.71]	0.10 [0.09–0.11]	0.33 [0.32–0.34]	0.26 [0.25–0.28]	0.06 [0.03–0.08]
	**Stroma**	**Endothelium**	**Epithelium**	**Apoptosis**	**Micro F_1_**	**Average F_1_**
**Intraobserver**	0.51 [0.37–0.64]	0.71 [0.59–0.84]	0.92 [0.77–1.07]	0.68 [0.57–0.79]	0.86 [0.86–0.86]	0.65 [0.61–0.70]
**Interobserver**	0.34 [0.27–0.41]	0.45 [0.34–0.57]	0.91 [0.77–1.05]	0.60 [0.51–0.70]	0.80 [0.80–0.80]	0.54 [0.50–0.58]
**Hover-Net (PUMA)**	0.31 [0.29–0.32]	0.15 [0.14–0.17]	0.13 [0.12–0.14]	0.22 [0.21–0.24]	0.61 [0.61–0.61]	0.27 [0.27–0.28]
**Hover-NeXt (PUMA)**	0.21 [0.20–0.22]	0.24 [0.23–0.25]	0.04 [0.03–0.05]	0.04 [0.04–0.05]	0.61 [0.61–0.61]	0.27 [0.27–0.27]

Inter- and intraobserver F_1_ scores are lower due to disagreement in the classification of plasma cells, melanophages, stroma nuclei, and endothelium. In the 12 randomly selected samples used to assess intra- and interobserver agreement, plasma cells and melanophages had a low incidence; 2 plasma cells and 14 melanophages were present (Table 4 ). Stroma, endothelium, and histiocytes had a higher incidence but were still subject to substantial disagreement.

**Table 4:** Nuclei count in intra- and interobserver ground-truth dataset consisting of 12 samples

	Tumor	Lymphocyte	Plasma cell	Histiocyte	Melanophage
**Nuclei count**	3,394	1,242	2	353	14
	**Neutrophil**	**Stroma**	**Endothelium**	**Epithelium**	**Apoptosis**
**Nuclei count**	103	110	161	173	196

### Heuristic postprocessing: combining tissue and nuclei predictions

**Figure 7:**
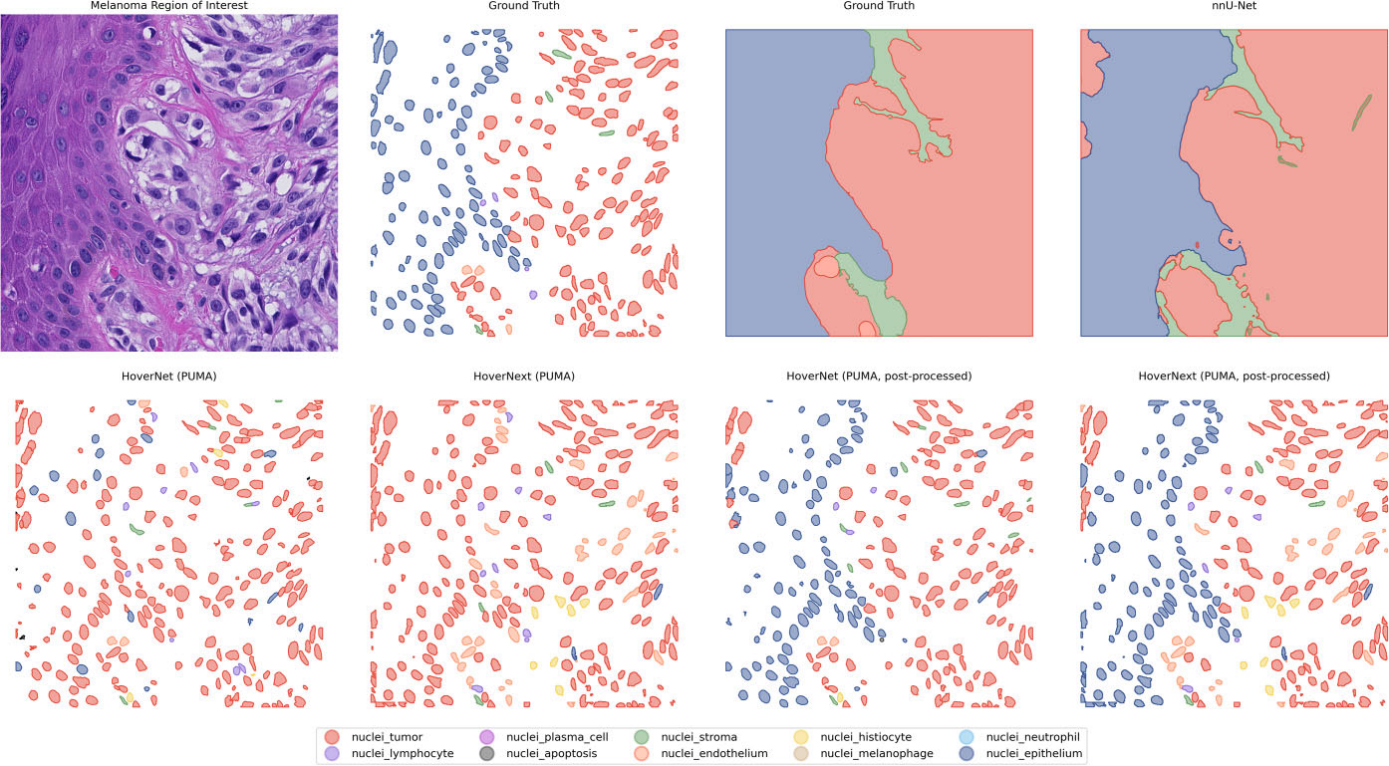
Visual results of heuristic postprocessing. For clarification, the ground truth and original predictions of Hover-Net and Hover-NeXt are displayed.

In the fourth experiment, we used the tissue mask as predicted by the nnU-Net model to improve nuclei segmentation. If a nucleus center was placed inside a tissue mask of the epidermis, the nuclei was classified as an epidermal nuclei. In addition, if a nucleus was present inside the blood vessel class, the nucleus was classified as an endothelial nucleus. In Hover-Net and Hover-NeXt, the average F1 score increased from 0.27 and 0.27 to 0.31 and 0.32, respectively. This is mainly due to an increase in the F_1_ score for epithelium (Table 5). Results for postprocessing are visualized in Fig 7.

**Table 5:** F_1_ scores for heuristic postprocessing of the Hover-Net and Hover-NeXt output compared with their non-postprocessing output

	Tumor	Lymphocyte	Plasma cell	Histiocyte	Melanophage	Neutrophil
**Hover-Net (PUMA)**	0.73 [0.72–0.73]	0.67 [0.66–0.67]	0.07 [0.06–0.09]	0.27 [0.26–0.27]	0.31 [0.29–0.33]	0.15 [0.13–0.18]
**Hover-Net (PUMA—postprocessed)**	0.73 [0.73–0.73]	0.67 [0.66–0.67]	0.07 [0.05–0.08]	0.27 [0.26–0.27]	0.32 [0.30–0.33]	0.15 [0.12–0.17]
**Hover-NeXt (PUMA)**	0.70 [0.70–0.71]	0.71 [0.70–0.71]	0.10 [0.09–0.11]	0.33 [0.32–0.34]	0.26 [0.25–0.28]	0.06 [0.03–0.08]
**Hover-NeXt (PUMA—postprocessed)**	0.71 [0.71–0.71]	0.71 [0.70–0.71]	0.10 [0.09–0.11]	0.33 [0.32–0.34]	0.27 [0.25–0.28]	0.06 [0.04–0.08]
	**Stroma**	**Endothelium**	**Epithelium**	**Apoptosis**	**Micro F_1_**	**Average F_1_**
**Hover-Net (PUMA)**	0.31 [0.29–0.32]	0.15 [0.14–0.17]	0.13 [0.12–0.14]	0.22 [0.21–0.24]	0.61 [0.61–0.61]	0.27 [0.27–0.28]
**Hover-Net (PUMA—postprocessed)**	0.31 [0.29–0.32]	0.2 [0.19–0.21]	0.44 [0.42–0.46]	0.22 [0.21–0.24]	0.62 [0.62–0.62]	0.31 [0.30–0.31]
**Hover-NeXt (PUMA)**	0.21 [0.20–0.22]	0.24 [0.23–0.25]	0.04 [0.03–0.05]	0.04 [0.04–0.05]	0.61 [0.61–0.61]	0.27 [0.27–0.27]
**Hover-NeXt (PUMA—postprocessed)**	0.21 [0.20–0.22]	0.25 [0.24–0.26]	0.52 [0.50–0.55]	0.05 [0.04–0.06]	0.62 [0.62–0.62]	0.32 [0.32–0.32]

## Discussion

In the present study, we show the methodology used to create the PUMA dataset, which is the first melanoma-specific nuclei and tissue dataset. In addition, we provide baseline benchmarks in which we show that the Hover-NeXt model trained with 3 classes (tumor, lymphocyte, and other) can detect lymphocytes almost on human interobserver-level performance. Finally, we show an example of the integration of tissue and nuclei annotations leading to improved nuclei segmentation. We believe that further improvement of deep learning models through this integration is possible, and therefore, the dataset will be used to organize the PUMA challenge.

Earlier studies regarding nuclei segmentation have been performed with multiple tissue datasets [17, 27] or other tissue-specific datasets in, for example, breast cancer [35] and colon cancer [36]. However, thus far, no melanoma-specific dataset is available. Since melanocytes and melanoma cells can mimic other cell types, such as lymphocytes and stroma cells, models created with this dataset are not fully applicable for use in melanoma histopathology [13, 18]. Models created with this dataset will be usable for the creation of prognostic and/or predictive biomarkers in melanoma histopathology.

The PUMA dataset combines tissue and nuclei segmentation. Earlier studies, such as the OCELOT challenge [37] and the MuTILs model [38], showed that incorporating tissue masks in nuclei prediction increased the capability of models that detect either tumor nuclei or TILs. With this dataset, the goal is to see whether implementation of tissue masks can improve multiclass nuclei segmentation for classes such epidermis, endothelium, tumor, and immune cell subsets. In this article, we demonstrated this by means of a simple postprocessing technique. However, other solutions might be passing information from a tissue segmentation model directly to a nuclei segmentation model or making use of multiple sequential models [37, 39]. To make use of the creativity and knowledge of the deep learning community, we initiated the PUMA challenge to assess how precise segmentation of melanoma samples can become. Participants in the PUMA challenge can choose to participate in 2 tracks. For track 1, the output needs to be nuclei segmentation for 3 classes (tumor, lymphocyte, and other) and tissue segmentation for all classes, whereas in track 2, participants need to segment all nuclei classes and tissue segmentation for all classes.

With the dataset, we also aimed to assess the performance of existing models usable in skin and/or melanoma histopathology. Due to unavailability, we were unable to assess the LUNIT scope IO model. However, we were able to evaluate the NN192 algorithm, which is used in multiple studies in which lymphocytes in melanoma histopathology were associated with a decreased chance of recurrence [40] and an increased chance of responding to immune checkpoint inhibition therapy [7]. Surprisingly, the NN192 model showed the lowest capability to detect lymphocytes. This might be due to variations in staining and scanners used in the studies performed earlier and the inability of the techniques used to correct for this. The Hover-Net and Hover-NeXt algorithm architectures can compensate for this by extensive data augmentation in the training (Hover-Net, Hover-NeXt) and inference stages (Hover-NeXt), accounting for better results with the out-of-the-box model trained on the PanNuke dataset. However, both models trained on the PanNuke dataset still showed diminished performance in more difficult to segment melanoma samples when compared to the Hover-NeXt model trained on the PUMA dataset itself.

A strength of this study is the annotation process by a medical expert and an expert dermatopathologist using context ROI to more accurately segment and classify nuclei. In addition, we validated the annotations with a second independent pathologist (G.B.). From this, it became clear that there is a low interobserver agreement in the less common classes, such as histiocytes, melanophages, and stroma. Melanophages are a subtype of histocytes that have phagocytosed melanin, resulting in a more pigmented appearance. However, there is a continuum between the cells, which could explain the difference in classification. Stroma cells, in addition, are present just like histiocytes in the tissue around the tumor, and it is not always possible to distinguish them from histiocytes on H&E-stained slides.

A second strong point of the study is the manual selection of ROIs. This allowed us to focus on immune cell subsets and less common nuclei and tissue classes. In addition, we tried to include regions with artifacts or much pigmentation. We believe this makes the dataset and the models more applicable to whole-slide images, which by nature have artifacts, unsharp regions, and, especially in the case of melanoma, pigmented more difficult to segment regions.

A downside of the dataset and the study is that all samples are, due to privacy regulations, from 1 scanner and a single hospital. However, 76 of 310 cases are consultation cases from referral hospitals, already leading to substantial staining variation. In addition, we believe that through stain normalization and data augmentation, models can become less sensible to domain-adversarial effects. This makes models trained on this dataset applicable to whole-slide images from other hospitals and scanner types.

Our study did not demonstrate high performance in segmentation of all nuclei classes. This is partly due to the used models losing discriminative ability by introducing more classes. In addition, some classes are less common in the dataset such as apoptosis or histiocytes. Furthermore, there is overlap between classes such as melanophages and histiocytes. We believe that further improvement of models is possible and hope that through leveraging the knowledge of the deep learning community in the PUMA challenge, this will become more clear.

In conclusion, we created a novel dataset with tissue and nuclei segmentations in advanced cutaneous melanoma. With this dataset, we showed that deep learning–based lymphocyte segmentation can achieve performance levels close to those of human interobserver agreement. In addition, we demonstrated that it is possible to more accurately segment nuclei classes by incorporating tissue predictions. However, we believe that further improvement is possible by further integration of tissue and nuclei segmentation. Future work in the PUMA challenge will demonstrate to what extent segmentation of different nuclei can be improved.

## Potential Implications

The PUMA dataset and the deep learning models created with this dataset can be used for biomarker generation in the treatment of melanoma. Thus far, it is known that lymphocytes have predictive value for melanoma recurrence and immune checkpoint inhibition treatment response [6, 7, 41]. However, this is done either by manual scoring or scoring by automated models, which are not generalizable to new unseen tissue and scanners. With this public dataset and the generalizable deep learning models developed from it, the next step will be to translate these models into clinical applications. This will result in more personalized treatment plans, such as de-escalation of immune checkpoint inhibition therapy in patients with advanced melanoma or a less intensive follow-up regimen in patients with primary melanoma. Next to the assessment of TILs as an explainable biomarker, this study will also enable future biomarker discovery studies through the assessment of other nuclei and tissue types.

In addition, this study adds a new step toward further improvement of nuclei segmentation deep learning models in H&E-stained histological slides by combining tissue segmentation models into multiclass nuclei segmentation models. This process is closer to how a pathologist evaluates histological slides and has already resulted in improved nuclei segmentation benchmark scores.

## Methods

### Dataset generation

For the dataset, digitized melanoma whole-slide H&E-stained images generated through regular diagnostic procedures were used from the archive of the UMC Utrecht. All slides were scanned with a Nanozoomer XR C12000–21/–22 (Hamamatsu Photonics; Hamamatsu) at 40× magnification with a resolution of 0.23 µm per pixel. Out of 310 cases in the dataset, 76 are consultation cases from referral hospitals or general practitioners, leading to variation in used staining protocols. From each slide, a 40× magnified ROI of 1,024 × 1,024 pixels was selected for annotation. In addition, a context ROI of 5,120 × 5,120 pixels was sampled to provide information about the broader context for the annotation process. Selection was done by a trained medical expert (M.S.) and subsequently verified by an expert dermatopathologist (W.B.). Manual ROI selection ensured diverse tissue and nuclei types and the inclusion of more difficult to segment areas due to blurring, pigmentation, and scanning artifacts.

Nuclei segmentations were generated with Hover-Net pretrained on the PanNuke dataset [16, 17]. Manual annotation adjustment was performed by author M.S. using QuPath with the following nuclei categories: tumor, stroma, vascular endothelium, histiocyte, melanophage, lymphocyte, plasma cell, neutrophil, apoptotic, and epithelium [30]. Annotation categories were based on earlier datasets. In addition, we chose categories based on possible predictive value. All annotations were checked and corrected where needed by a dermatopathologist (W.B.). Intra- and interobserver agreement (by pathologist G.B.) were determined on 12 randomly selected ROIs.

Tissue segmentations were created manually with QuPath by author M.S. using the following categories: tumor, stroma, epidermis, necrosis, blood vessel, and background. Annotation categories are based on the approach used by Hwang et al. [15], who segmented TILs in tumor and stroma areas, and the guidelines from the International Immuno-Oncology Biomarker Working Group [42]. Annotations are checked and corrected when needed by a dermatopathologist (W.B.). Intra- and interobserver agreement (by pathologist G.B.) were determined on 12 randomly selected ROIs to ensure the inclusion of all tissue types.

### Benchmark models

#### Nuclei segmentation

To establish baseline nuclei segmentation benchmarks, we performed nuclei segmentation for 2 sets of experiments. The first experiment compares models that output 3 nuclei categories: tumor, lymphocyte, and other. The second experiment is an analysis of the segmentation of all individual nuclei categories. Model training was performed with 5-fold cross-validation on the public training dataset without adjusting training parameters or data augmentation of the algorithm used. Inference was performed on the 94 ROIs of the final hidden test set of the PUMA challenge. The remaining 10 ROIs will be used in the PUMA challenge for sanity checking of submitted models.

For the first experiment, the following models were compared: NN192, Hover-Net trained on the PanNuke dataset, Hover-Net trained on the PUMA dataset, Hover-Next trained on the PanNuke dataset, and Hover-Next trained on the PUMA dataset [16, 17, 41, 43]. The NN192 model outputs 4 categories: tumor, lymphocytes, stroma, and other. From this output, the stroma and other categories were merged into one other category. The models trained on the PanNuke dataset classify nuclei into 5 categories: neoplastic, nonneoplastic epithelial, inflammatory, connective, and apoptotic. For the benchmark comparison, neoplastic and inflammatory nuclei were used; nonneoplastic epithelial, connective, and apoptotic were merged into the other category. For training on the PUMA dataset, the plasma cell and lymphocyte categories were merged into a single lymphocyte category, and the remaining classes were combined into the other category. For the second experiment, Hover-Net and Hover-NeXt were trained on all samples and compared.

For the calculation of the F1 score, the center distance between the predicted nuclei and the ground-truth nuclei was used. For each ground-truth nucleus, predictions within 15 pixels (3.3 μm) were identified. This radius is smaller than the average size of lymphocytes, which form the smallest nuclei in the dataset. Matching was performed based on the highest predictive score (if available) or the shortest distance. After matching, the ground truth was censored until all ground-truth nuclei were either matched or classified as a false negative. Using the identified true positives, false positives, and false negatives, precision (all correct predictions divided by all predictions) and recall (all correct predictions divided by all ground-truth nuclei) were calculated. The class F1 score was computed as the harmonic mean of precision and recall, ranging from 0 to 1. Finally, to compare models, micro F_1_ (aggregation of true positive, false positive, and false negetative over all classes, followed by F1 score calculation) and Average F_1_ (the average of class F_1_ scores) were calculated [44]. Results are shown with a 95% confidence interval, which is calculated through bootstrapping the samples.

#### Tissue segmentation

For tissue segmentation, nnU-Net and Mask2Former were used to establish baseline benchmarks [32, 34]. For training of nnU-Net, the same 5-fold cross-validation was used to create an ensemble model. Mask2Former was pretrained on the COCO instance segmentation task using a Swin Transformer backbone. Images were resized to 512 × 512 pixels before loading into the model. For our experiment, we replaced the backbone with the UNI pathology foundation model, which is better able at feature extraction from H&E-stained histopathology, after which we fine-tuned the model on the whole training dataset [33, 34, 45]. Both models were used for inference on the final hidden test set from the PUMA challenge.

Both models and intra- and interobserver agreement were evaluated using the Dice score. The Dice score is a harmonic mean between 0 and 1, in which 1 is a perfect segmentation prediction, and 0 is no correct prediction. This can result in inflated high average Dice scores if a tissue class is present in only a few samples, as the Dice score is 1 in the case of a correct absent prediction. To accommodate this, we calculated not only the average Dice score over all samples but also the micro-average Dice. This is the Dice score for all predictions concatenated along 1 axis, resulting in a prediction mask of 1,024 × 96.256 pixels. Results are shown with a 95% confidence interval, which is generated through bootstrapping the sample results.

#### Postprocessing

For postprocessing nuclei, centers were calculated using the Point function from the Python Shapely library. From these points, the spatial location inside a tissue mask prediction was determined by using the GeoPandas Python Package. Based on this location, the classification label of the nuclei was adjusted.

## Availability of Source Code and Requirements

Project name: PUMA

Project homepage: https://puma.grand-challenge.org/ [28]

Operating system: Platform independent

Programming language: Python 3.10

Other requirements: Hover-Net [16, 46], Hover-NeXt [43] training code [47] and inference code [48], the NN192 [41] classification algorithm [49], QuPath 0.1.2 [30, 50], QuPath 0.5.0 [30, 51], nnU-Net [32, 52] and Mask2Former [34, 53]

The code and weights for the PUMA challenge inference baseline solutions and metric calculation can be found in Zenodo [54] and GitHub repository for the following, all of which have been archived in Software Heritage: Evaluation Track 1 [ 55], Evaluation Track 2 [56], Baseline Track 1 [57], and Baseline Track 2 [58].

QuPath scripts to import .geoJSONs for viewing and exporting annotations as masks can be found in Zenodo [54].

No changes were made to the training code for the used algorithms.

License: The PUMA codebase is licensed with a CC0 1.0 license (dataset) and the MIT license.

Restrictions to use by nonacademics: Both the CC0 1.0 license (dataset) and the MIT license (codebase) allow for non-commercial use. License terms can be reviewed for details.

## Abbreviations

H&E: hematoxylin and eosin; PUMA: Panoptic Segmentation of nUclei and tissue in advanced MelanomA; ROI: region of interest; TIL: tumor infiltrating lymphocyte.

## Declaration

The Biobank Research Ethics Committee (TCBio) UMC Utrecht confirms that it has reviewed the release file in accordance with the UMC Utrecht Biobank Regulations and all other applicable regulations and laws. Based on the requirements as defined in these regulations and laws, the TCBio UMC Utrecht hereby issues an approval of the aforementioned dataset (reference number TCBio 23-270/U-B).

## Supplementary Material

giaf011_GIGA-D-24-00434_Original_Submission

giaf011_GIGA-D-24-00434_Revision_1

giaf011_GIGA-D-24-00434_Revision_2

giaf011_Response_to_Reviewer_Comments_Original_Submission

giaf011_Response_to_Reviewer_Comments_Revision_1

giaf011_Reviewer_1_Report_Original_Submission Shan Raza -- 11/20/2024

giaf011_Reviewer_2_Report_Original_Submission Amirreza Mahbod -- 11/21/2024

## Data Availability

The PUMA training dataset is available on Zenodo [31]. Joining the PUMA challenge is possible through the grand challenge platform [28]. The preliminary and final test set will stay hidden until 10 October 2029 due to the PUMA challenge still being open for submissions until then. Readers can request earlier access to the hidden dataset for educational or collaborative purposes by contacting the corresponding author via email. DOME-ML (Data, Optimisation, Model, and Evaluation in Machine Learning) annotation supporting the current study is available through DOME Registry [59].
