# Supplementary material for: A novel dataset for nuclei and tissue segmentation in melanoma with baseline nuclei segmentation and tissue segmentation benchmarks
Source: Gigascience. 2025 Feb 19;14:giaf011. doi: 10.1093/gigascience/giaf011 (PMC11837757; doi:10.1093/gigascience/giaf011)

GigaScience

A novel dataset for nuclei and tissue segmentation in melanoma with baseline nuclei segmentation and tissue segmentation benchmarks

--Manuscript Draft--

**Manuscript Number:**	GIGA-D-24-00434R1
**Full Title:**	A novel dataset for nuclei and tissue segmentation in melanoma with baseline nuclei segmentation and tissue segmentation benchmarks
**Article Type:**	Research
**Funding Information:**	Hanarth Fonds / prof. dr. Karijn P.M. Suijkerbuijk
**Abstract:**	Background<br><br>Melanoma is an aggressive form of skin cancer in which tumor-infiltrating lymphocytes (TILs) are a biomarker for recurrence and treatment response. Manual TIL assessment is prone to interobserver variability, and current deep learning models are not publicly accessible or have low performance. Deep learning models, however, have the potential of consistent spatial evaluation of TILs and other immune cell subsets with the potential of improved prognostic and predictive value. To make the development of these models possible, we created the Panoptic Segmentation of nUclei and tissue in advanced MelanomA (PUMA) dataset and assessed the performance of several state-of-the-art deep learning models. In addition, we show how to improve model performance further by using heuristic post-processing in which nuclei classes are updated based on their tissue localization.<br><br>Results<br><br>The PUMA dataset includes 155 primary and 155 metastatic melanoma H&E stained regions of interest with nuclei and tissue annotations from a single melanoma referral institution. The Hover-NeXt model, trained on the PUMA dataset, demonstrated the best performance for lymphocyte detection, approaching human interobserver agreement. In addition, heuristic post-processing of deep learning models improve the detection of non-common classes, such as epithelial nuclei.<br><br>Conclusion<br><br>The PUMA dataset is the first melanoma specific dataset that can be used to develop melanoma-specific nuclei and tissue segmentation models. These models can, in turn, be used for prognostic and predictive biomarker development. Incorporating tissue and nuclei segmentation is a step towards improved deep learning nuclei segmentation performance. To support the development of these models, this dataset is used in the PUMA challenge.
**Corresponding Author:**	Mark Schuiveling, MD<br>UMC Utrecht: Universitair Medisch Centrum Utrecht<br>Utrecht, NETHERLANDS
**Corresponding Author Secondary Information:**	
**Corresponding Author's Institution:**	UMC Utrecht: Universitair Medisch Centrum Utrecht
**Corresponding Author's Secondary Institution:**	
**First Author:**	Mark Schuiveling, MD
**First Author Secondary Information:**	
**Order of Authors:**	Mark Schuiveling, MD
	Hong Liu
	Daniel Eek

Powered by Editorial Manager® and ProduXion Manager® from Aries Systems Corporation

	Gerben E. Breimer, MD, PhD
	Karijn P.M. Suijkerbuijk, MD, PhD
	Willeke A.M. Blokx, MD, PhD
	Mitko Veta, PhD
**Order of Authors Secondary Information:**	
**Response to Reviewers:**	Re: GIGA-D-24-00434, "A Novel Dataset for Nuclei and Tissue Segmentation in Melanoma with baseline nuclei segmentation and tissue segmentation benchmarks"<br><br>Dear, Nicole Nogoy, Ph.D,<br><br>Thank you for your evaluation of our manuscript "A Novel Dataset for Nuclei and Tissue Segmentation in Melanoma with baseline nuclei segmentation and tissue segmentation benchmarks". We highly appreciate the thoughtful feedback and constructive suggestions from the reviewers and editors, which have helped us to revise our manuscript.<br><br>We have addressed each comment from the reviewers and editors point-by-point below. We hope that the revisions will meet the expectations of the journal and contribute positively to the quality of our data and its description.<br><br>We look forward to your response and thank you for the opportunity to resubmit our manuscript.<br><br>Yours sincerely, on behalf of all co-authors,<br><br>Mark Schuiveling, MD MSc<br>Dr. Mitko Veta, PhD<br><br>Medical Image Analysis,<br>Department of Biomedical Engineering,<br>Eindhoven University of Technology,<br>Eindhoven, the Netherlands<br><br>--<br><br>Comments from the Reviewers<br>Reviewer #1:<br>The paper presents a dataset for nuclei and tissue segmentation in melanoma with about 155 primary and 155 metastatic cases from a single referral institution. The authors use Hover-NeXt model to demonstrate the performance of lymphocyte detection. The post processing further improves the detection results. The lymphocyte detection is used to estimate TILs scores as there is evidence that TILs are associated with reduced rates in primary melanoma. The authors have used original pipelines for training HoverNet and HoVerNext so the comparison is fair.<br><br>1.For metrics however, I would suggest to add additional metrics for a fair comparison for segmentation. Is DICE loss used in training the network? If yes then the comparison is not fair and additional metrics should be used such as pixel accuracy, F1-score, Hausdorff distance and panoptic quality to estimate the performance of segmentation and classification for both tissue and nuclei segmentation. DICE loss does not give an overall picture.<br><br>We thank the reviewer for their assessment of our manuscript and valuable suggestions. When training nnU-Net and Mask2Former no changes were made to the original code. This means that for nnU-Net training a complex loss combining the sum of cross-entropy and Dice loss is used. Regarding Mask2Former, a complex loss based on dice loss, cross-entropy and focal loss is used.<br><br>Regarding the metrics for tissue segmentation:<br>We deliberately chose DICE score as a metric for tissue segmentation due as it is a

commonly used (if not the most common) metric for medical image segmentation tasks [1]. As both our segmentation models use Dice loss in their loss calculation, we still believe it to be fair. In addition, due its common use it makes our results easily interpretable. Haussdorf distance is not a suitable metric whereas it does not take holes in segmentation into account.

Regarding the metrics for nuclei segmentation:
We used F1 score based on centroid distance for nuclei segmentation, as the small size of nuclei makes metrics such as panoptic quality and aggregated Jaccard index unsuitable due their dependence on Intersection over Union. Foucart et al. (2023) demonstrated that IoU based metrics such as panoptic quality are overly sensitive for small annotations, leading to substantial metric changes from minor segmentation differences [2]. This results in a higher chance in misclassifications for small objects such as lymphocytes. Therefore, we used classification based on centroid distance as used by the Ocelot challenge, which follow the Metrics-Reloaded guidelines as stated by Maier-Hein et al [1].

2.Combining the tissue and nuclei segmentation/classification improves the results as expected. One of the major issues I had while reading the paper was to understand what data has been made publicly available. In data availability section, it says training dataset has been made available but then it says 104 annotated ROIs are hidden and will remain so for another five years. I am not clear if the hidden ROIs include the training dataset? Or the hidden ROIs are the test set? If this is the test set then it makes sense.

We understand the confusion this generates. We therefore adjusted the manuscript with the following with more clearly describes what part of the dataset is publicly available. In addition, it more clearly describes the dataset division which was also confusing as stated by the second reviewer.
“ From the total dataset of 310 ROIs, a training set consisting of 103 primary and 103 metastatic ROI has been made publicly available [3]. The remaining 104 ROIs are not publicly available as they are used in the PUMA challenge. In this challenge, 10 ROIs are used a preliminary test set for testing of functioning of models. The remaining 94 samples are used as an independent test set for final metric calculations. To be able to compare results from the PUMA challenge to our paper results in this paper are shown for this independent test set.

The tissue distribution for the training, preliminary and final test set is visualized in Figure 3. In primary samples, more tumoral stroma is present compared to metastatic samples. The epidermis and necrotic area are underrepresented in both datasets.

Figure 3. Distribution of tissue class area in the PUMA dataset.
“

3.I have also looked at the grand challenge website. I couldn't find a link between the paper "data availability" section and the challenge website. I would suggest to make it clear in the data availability. You can specify training data set available at ..., validation available at ... and test can be run on grand challenge. If that's the case. The other point as I mentioned above is related to the metrics used for evaluation they should be broad.

We agree with the reviewer that a link to the PUMA challenge needs to be added to the data availability statement. We therefore added this to data availability statement which resulted in the following:

“The PUMA training dataset is available on Zenodo at: https://zenodo.org/records/10940194. Joining the PUMA challenge is possible through the grand challenge platform at https://puma.grand-challenge.org/. The preliminary and final test set will stay hidden until October 10th 2029 due the PUMA challenge still

Powered by Editorial Manager® and ProduXion Manager® from Aries Systems Corporation

being open for submissions until then. Readers can request earlier access to the hidden dataset for educational or collaborative purposes by contacting the corresponding author via email."

Reviewer #2: *
General Comments: The paper is well-written and easy to follow.
We thank the reviewer for their kind words.

1.* Current Text: "We will use this dataset to organize the PUMA challenge in which the goal is to further improve model performance." --> Recommendation: Remove "will" as the PUMA challenge is already in progress.

We agree with the reviewer that this is no longer the correct statement. The abstract conclusion has been changed into the following:

"The PUMA dataset is the first melanoma specific dataset that can be used to develop melanoma-specific nuclei and tissue segmentation models. These models can, in turn, be used for prognostic and predictive biomarker development. Incorporating tissue and nuclei segmentation is a step towards improved deep learning nuclei segmentation performance. To support the development of these models, this dataset is used in the PUMA challenge. "

2.* As this dataset is currently used for the PUMA challenge, further details about the challenge, including a link to the challenge website, should be added to the text.

We agree that this should be added. As stated in the response to reviewer 1 a link to the challenge has been added to the data availability statement. In addition, we added a link to the data description:
"The PUMA dataset consists of regions of interest (ROIs) with nuclei and tissue annotations. ROIs originate from H&E stained histological slides of melanoma specimens. The dataset's goal is to facilitate the development of deep learning models capable of segmenting nuclei and tissue. To stimulate the use of the dataset and create novel deep learning models, the dataset will also be used for a medical image analysis challenge hosted on the grand-challenge.org platform (accessible through https://puma.grand-challenge.org/). The models created with the dataset and the PUMA challenge, in turn, can be used for prognostic biomarker generation in melanoma treatment. "

3.* Figure 2 shows the distribution of nuclei and sampled tissue in the PUMA dataset for primary melanoma regions (half of the dataset). However, what about the distribution for metastatic ROIs (the other half)?

Figure 2 shows the distribution for the primary samples (in bold). The other samples are all metastatic samples. Due to the confusion we updated the figure caption:

"Figure 2. Distribution of nuclei and sampled tissue in the PUMA dataset for primary melanoma regions of interest and metastatic melanoma regions of interest stratified according to metastasis location. The primary samples are depicted as the last row of each graph. "

4.* Current Text: "We used 5-fold cross-validation while training and report the results of inference on the 94 ROIs of the final independent test set used in the PUMA challenge." Earlier in the text, it states: "The test set consists of 50,490 nuclei in 52 primary and 52 metastatic ROIs" (104 images). Upon careful reading, I understood that

Powered by Editorial Manager® and ProduXion Manager® from Aries Systems Corporation

94 images are used as the final test set, while 10 are used for validation. To prevent confusion, this information should be stated clearly and as early as possible in the text, such as on page 6.

We agree that this is not clear in the current manuscript. We therefore adjusted this on page 6 to make it more clear. The changes made are visible in the response of question 2 from reviewer 1.

5.* Since this dataset is specifically designed for histological image analysis of melanoma, other publicly available nuclei instance segmentation (or classification) datasets, such as NuInsSeg or CryoNuSeg, which include H&E-stained skin images, should be mentioned. These datasets can be used for tasks like pre-training to enhance the performance of DL-based approaches on the PUMA dataset.
https://www.sciencedirect.com/science/article/pii/S0010482521001438
https://www.nature.com/articles/s41597-024-03117-2

We agree that these datasets, which also include skin samples, should also be mentioned. Next to this dataset, we also added a section about the SegPath dataset as this also contains a melanoma sample. The following has been added to the introduction of the manuscript:
“ As for datasets which can be used to train deep learning models usable in melanoma, the NuInsSeg [4] and CryoNuSeg [5] datasets also partly consist of skin samples. However these datasets do not have separate classes for nuclei such as tumor or lymphocyte rendering them less useful for creating deep learning nuclei classification algorithms. In contract, the SegPath dataset does classify nuclei into distinct groups using a ground truth based on immunofluorescence staining. However, it contains only one melanoma sample with CD3/CD20 annotations [6]. “

6.* The hyperparameters of the used nn-UNet should be added to the text. Has any ensembling been employed?

nnU-Net is a segmentation framework that automatically adapts to the characteristics of a given dataset. By analyzing the provided training data, it configures a U-Net-based segmentation pipeline tailored to the dataset[7]. We utilized nnU-Net as-is, without modifying its default hyperparameters, which is why no hyperparameters were explicitly added to the Methods section.
In our Methods, we state:
"For training of nnU-Net the same fivefold cross validation was used to create an ensemble model."
This follows the standard practice in nnU-Net's training code and reflects the framework's default ensembling strategy.

7.* The nn-UNet is primarily designed for 3D datasets. What motivated its use for 2D image segmentation in this work rather than opting for more advanced architectures specifically designed for 2D segmentation, such as those based on vision transformers?

nnU-Net is a versatile framework capable of handling both 2D and 3D datasets across various imaging modalities [7]. It has been successfully applied to 2D segmentation tasks in histopathology with high performance [8].
In addition, we explored a vision transformer based architecture with a Mask2Former model with the backbone replaced by the UNI pathology foundation model [7,9,10]. Nn-UNet, however, had better performance as it tunes automatically all its hyperparameters during the training which for small datasets such as ours gives better performance.

Powered by Editorial Manager® and ProduXion Manager® from Aries Systems Corporation

8.* The results reported in Table 2 are based on the F1 score. Given that the challenge name implies panoptic segmentation of nuclei, why were common evaluation metrics for nuclei instance segmentation and classification tasks, such as Average Panoptic Quality or Average AJI, not used? Is the F1 score sensitive to the ability of the model to separate overlapping nuclei?

We used F1 score based on centroid distance for nuclei segmentation, as the small size of nuclei makes metrics such as panoptic quality and aggregated Jaccard index unsuitable due their dependence on Intersection over Union. Foucart et al. (2023) demonstrated that IoU based metrics such as panoptic quality are overly sensitive for small annotations, leading to substantial metric changes from minor segmentation differences [2]. This results in a higher chance in misclassifications for small objects such as lymphocytes. Therefore, we used classification based on centroid distance as used by the Ocelot challenge, which follow the Metrics-Reloaded guidelines as stated by Maier-Hein et al [1].
This metric is also able to separate overlapping nuclei due the assessment of each individual nuclei from a .geoJSON (or JSON in the PUMA challenge) with polygon coordinates.

9.* A challenge participant has noted a mismatch between the annotation and image file training_set_metastatic_roi_103 ((https://grand-challenge.org/forums/forum/panoptic-segmentation-of-nuclei-and-tissue-in-advanced-melanoma-737/topic/potential-annotation-issue-in-file-training_set_metastatic_roi_103-2877/). Please comment on this issue and confirm that no such problems exist in your validation and test datasets.

We regret that this has happened. We deleted the sample from the dataset to level the playing field for the PUMA challenge. After the challenge deadline (1st of March 2025) has been passed we will upload the correct image. As an additional check, we added an overlay of the preliminary and final test set to the rebuttal to show that there are no more errors in the dataset. This is downloadable from surfdrive with the following link: https://surfdrive.surf.nl/files/index.php/s/PHjbEjbokfNx244 (password gigascience).

10.* While annotations are currently provided in .geojson format, it would be beneficial to include them in other conventional formats, such as .png files or as overlays on the images. This could be achieved either by including additional files in the dataset or by adding a Qupath script in the GitHub repository to save the annotations for all images in various formats.

We added qupath scripts to the zenodo repository with the weights which give users the possibility to export the annotations as masks. A link to this has been added to the availability of source code and requirements section:
“ Qupath scripts to import .geoJSONs for viewing and exporting annotations as masks can be found here: https://zenodo.org/records/13897135.“

11.* From my understanding, the context ROIs of 5120×5120 pixels are provided only for the training set. If a model relies on this information during training, similar data would also be needed for the validation and test sets. Are context ROIs provided for validation and test data as well? Please clarify.

We understand that this might seem strange. Our idea was that these context regions of interest could be used to generate more data through inference on these samples. Giving the context regions of interest for the validation and test set would give participants the opportunity to reconstruct the ground truth from these data sets. This would not be in line with the goal of the challenge which is generating the best possible deep learning model for inference on previously unseen data.
However, after the 5 year embargo this data will be released together with the

Powered by Editorial Manager® and ProduXion Manager® from Aries Systems Corporation

	annotations and regions of interest of the test set.<br><br>12.* On page 26, the link https://pumachallenge.com/ is not working. Please update or replace it with a functional link.<br><br>We replaced the link with a link to the PUMA grand challenge website. (Project home page: https://puma.grand-challenge.org/)<br><br>13.* "This research was funded by an unrestricted grant of Stichting Hanarth Fonds, The Netherlands.." --> Please remove one of the dots at the end<br><br>The dot was removed.<br><br>---------------------------------------<br><br>References:<br>1. Maier-Hein L, Reinke A, Godau P, Tizabi MD, Buettner F, Christodoulou E, et al.. Metrics reloaded: recommendations for image analysis validation. Nat Methods. Nature Publishing Group; 2024; doi: 10.1038/s41592-023-02151-z.<br>2. Foucart A, Debeir O, Decaestecker C. Panoptic quality should be avoided as a metric for assessing cell nuclei segmentation and classification in digital pathology. Sci Rep. Nature Publishing Group; 2023; doi: 10.1038/s41598-023-35605-7.<br>3. Schuiveling M. Melanoma Histopathology Dataset with Tissue and Nuclei Annotations. Zenodo; 2024; doi: 10.5281/zenodo.10940193.<br>4. Mahbod A, Polak C, Feldmann K, Khan R, Gelles K, Dorffner G, et al.. NuInsSeg: A fully annotated dataset for nuclei instance segmentation in H&E-stained histological images. Sci Data. Nature Publishing Group; 2024; doi: 10.1038/s41597-024-03117-2.<br>5. Mahbod A, Schaefer G, Bancher B, Löw C, Dorffner G, Ecker R, et al.. CryoNuSeg: A dataset for nuclei instance segmentation of cryosectioned H&E-stained histological images. Computers in Biology and Medicine. 2021; doi: 10.1016/j.compbiomed.2021.104349.<br>6. Komura D, Onoyama T, Shinbo K, Odaka H, Hayakawa M, Ochi M, et al.. Restaining-based annotation for cancer histology segmentation to overcome annotation-related limitations among pathologists. Patterns. 2023; doi: 10.1016/j.patter.2023.100688.<br>7. Isensee F, Jaeger PF, Kohl SAA, Petersen J, Maier-Hein KH. nnU-Net: a self-configuring method for deep learning-based biomedical image segmentation. Nat Methods. Nature Publishing Group; 2021; doi: 10.1038/s41592-020-01008-z.<br>8. Spronck J, Gelton T, Eekelen L van, Bogaerts J, Tessier L, Rijthoven M van, et al.. nnUNet meets pathology: Bridging the gap for application to whole slide images and computational biomarkers. Medical Imaging with Deep Learning. PMLR; p. 1859–74.<br>9. : Towards a general-purpose foundation model for computational pathology | Nature Medicine. https://www.nature.com/articles/s41591-024-02857-3 Accessed 2024 Sep 23.<br>10. Cheng B, Misra I, Schwing AG, Kirillov A, Girdhar R. Masked-attention Mask Transformer for Universal Image Segmentation. arXiv;
**Additional Information:**	
**Question**	**Response**
Are you submitting this manuscript to a special series or article collection?	No
**Experimental design and statistics**<br><br>Full details of the experimental design and statistical methods used should be given	Yes

Powered by Editorial Manager® and ProduXion Manager® from Aries Systems Corporation

in the Methods section, as detailed in our Minimum Standards Reporting Checklist. Information essential to interpreting the data presented should be made available in the figure legends.<br><br>Have you included all the information requested in your manuscript?	
**Resources**<br><br>A description of all resources used, including antibodies, cell lines, animals and software tools, with enough information to allow them to be uniquely identified, should be included in the Methods section. Authors are strongly encouraged to cite Research Resource Identifiers (RRIDs) for antibodies, model organisms and tools, where possible.<br><br>Have you included the information requested as detailed in our Minimum Standards Reporting Checklist?	Yes
**Availability of data and materials**<br><br>All datasets and code on which the conclusions of the paper rely must be either included in your submission or deposited in publicly available repositories (where available and ethically appropriate), referencing such data using a unique identifier in the references and in the “Availability of Data and Materials” section of your manuscript.<br><br>Have you have met the above requirement as detailed in our Minimum Standards Reporting Checklist?	No
If not, please give reasons for any omissions below.<br><br>as follow-up to "**Availability of data and**	Part of the dataset has been publicly released, while a section of the data has been withheld for the organization of the PUMA challenge. As this is a rolling challenge, the withheld data will remain hidden for five years. Upon request, the hidden test set can be sent to the editorial team for review.

**materials**<br><br>All datasets and code on which the conclusions of the paper rely must be either included in your submission or deposited in publicly available repositories (where available and ethically appropriate), referencing such data using a unique identifier in the references and in the "Availability of Data and Materials" section of your manuscript.<br><br>Have you have met the above requirement as detailed in our Minimum Standards Reporting Checklist?<br><br>"	
GigaScience has policies and guidelines in place for the use of generative AI-writing tools such as ChatGPT. If you have used such writing tools to assist with writing the manuscript this must be declared and cited in the text. Authors should not list AI-writing tools and other AI-assisted technologies as an author or co-author and should acknowledge that they are fully responsible for text generated or refined by AI-writing tools.<br><br>A summary of use (particularly in the introduction or among methods) needs to be included at the end of the paper, and the outputs should also be included as a supplementary file hosted in GigaDB or other open repositories. Please read our guidelines for more information. <br>By submitting to GigaScience, you are aware of the journal's AI-writing tools policy, and if you have declared use of such tools below, you have acknowledged this where appropriate in your manuscript and have made a summary of use and outputs available. <br><br>**AI-assisted writing tools have been used in the preparation of this manuscript?<br>** 	No

Manuscript

Click here to access/download;Manuscript;A Novel Dataset for Nuclei and Tissue clean.docx

A novel dataset for nuclei and tissue segmentation in melanoma with baseline nuclei segmentation and tissue segmentation benchmarks

Mark Schuiveling, MD[1]; Hong Liu[2]; Daniel Eek[3]; Gerben E. Breimer, MD, PhD [4]; Karijn P.M. Suijkerbuijk[5], MD, PhD; Willeke A.M. Blokx[6*], MD, PhD; Mitko Veta, PhD[7*]

Affiliations

1. Department of Medical Oncology, University Medical Center Utrecht, Utrecht University, Heidelberglaan 100, 3584 CG Utrecht, the Netherlands. m.schuiveling@umcutrecht.nl
2. Medical Image Analysis, Department of Biomedical Engineering, Eindhoven University of Technology, Eindhoven, the Netherlands. h.liu2@tue.nl
3. Medical Image Analysis, Department of Biomedical Engineering, Eindhoven University of Technology, Eindhoven, the Netherlands. d.b.eek@student.tue.nl
4. Department of Pathology, University Medical Center Utrecht, Utrecht University, Heidelberglaan 100, 3584 CG Utrecht, the Netherlands. g.e.breimer-2@umcutrecht.nl
5. Department of Medical Oncology, University Medical Center Utrecht, Utrecht University, Heidelberglaan 100, 3584 CG Utrecht, the Netherlands. k.suijkerbuik@umcutrecht.nl
6. Department of Pathology, University Medical Center Utrecht, Utrecht University, Heidelberglaan 100, 3584 CG Utrecht, the Netherlands. w.a.m.blokx@umcutrecht.nl
7. Medical Image Analysis, Department of Biomedical Engineering, Eindhoven University of Technology, Eindhoven, the Netherlands. m.veta@tue.nl

* These authors contributed equally to this work.

Corresponding author: Mark Schuiveling, MD, Department of Medical Oncology, University Medical Center Utrecht, Utrecht University, Postbus 85500, 3508 GA Utrecht, the Netherlands m.schuiveling@umcutrecht.nl)

Abstract

Background
Melanoma is an aggressive form of skin cancer in which tumor-infiltrating lymphocytes (TILs) are a biomarker for recurrence and treatment response. Manual TIL assessment is prone to interobserver variability, and current deep learning models are not publicly accessible or have low performance. Deep learning models, however, have the potential of consistent spatial evaluation of TILs and other immune cell subsets with the potential of improved prognostic and predictive value. To make the development of these models possible, we created the Panoptic Segmentation of nUclei and tissue in advanced MelanomA (PUMA) dataset and assessed the performance of several state-of-the-art deep learning models. In addition, we show how to improve model performance further by using heuristic post-processing in which nuclei classes are updated based on their tissue localization.

Results
The PUMA dataset includes 155 primary and 155 metastatic melanoma H&E stained regions of interest with nuclei and tissue annotations from a single melanoma referral institution. The Hover-NeXt model, trained on the PUMA dataset, demonstrated the best performance for lymphocyte detection, approaching human interobserver agreement. In addition, heuristic post-processing of deep learning models improve the detection of non-common classes, such as epithelial nuclei.

Conclusion
The PUMA dataset is the first melanoma specific dataset that can be used to develop melanoma-specific nuclei and tissue segmentation models. These models can, in turn, be used for prognostic and predictive biomarker development. Incorporating tissue and nuclei segmentation is a step towards improved deep learning nuclei segmentation performance. To support the development of these models, this dataset is used in the PUMA challenge.

Keywords:

Melanoma, H&E stained histopathology, nuclei segmentation, tissue segmentation, TILs

Background

Melanoma is an aggressive form of skin cancer with increasing incidence [1]. Primary melanoma is treated with surgical excision, whereas advanced, metastasized melanoma is most commonly treated with immune checkpoint inhibition therapy, a form of cancer immunotherapy. However, half of the advanced melanoma patients do not respond to this treatment, which is costly and potentially toxic [2–5].

Previous studies showed that tumor infiltrating lymphocytes (TILs) in hematoxylin & eosin (H&E) stained slides of metastatic melanoma before the start of treatment are associated with a higher chance of response to immune checkpoint inhibition and an increase in survival [6,7]. In addition, TILs are associated with reduced recurrence rates in primary melanoma [8]. Therefore, assessment of TILs can serve as a prognostic biomarker in melanoma treatment.

Currently, TILs are scored manually, either by estimating a stromal percentage or through using a multitier system like the Clark score, which categorizes TILs as absent, non-brisk, or brisk [6,9,10]. However, substantial interobserver variability exists among pathologists [6,11,12]. A deep learning-based assessment could result in a more precise, consistent and fine-grained assessment of TILs.

To date, two deep learning-based models have been used to evaluate TILs in melanoma histopathology. The first model, NN192, uses watershed segmentation followed by a fully connected neural network [7]. However, this older, suboptimal technique results in lower performance [13]. The second model, LUNIT's Scope IO pan tumor model, is not melanoma specific and not publicly available [14,15]. Publicly available models, such as Hover-Net pretrained on the PanNuke dataset (which includes skin samples) can be used to detect TILs in melanoma, but have suboptimal performance. This is due to the model not being melanoma-specific [16,17]. As a result, misclassifications occur because melanoma cells can resemble other cell types, such as stroma or lymphocytes [13,18,19].

As for datasets which can be used to train deep learning models usable in melanoma, the NuInsSeg [20] and CryoNuSeg [21] datasets also partly consist of skin samples. However these datasets do not have separate classes for nuclei such as tumor or lymphocyte rendering them less useful for creating deep learning nuclei classification algorithms. In contract, the SegPath dataset does classify nuclei as lymphocytes using a ground truth based on immunofluorescence staining. However, it contains only one melanoma sample with CD3/CD20 annotations [22].

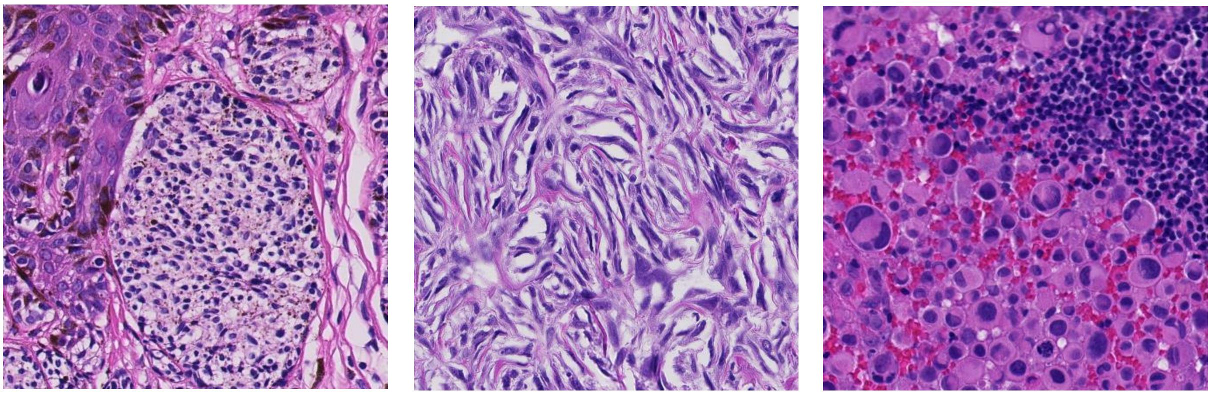

Figure 1. Examples of melanoma histopathology appearances: a lymphocyte-like morphology on the left, a stromal growth pattern in the center, and a tumor with large, variable nuclei on the right.

Next to the presence of (deep learning detected) TILs, the localization of TILs is of importance. In breast cancer and non-small cell lung cancer, TILs located within the tumor or intra-tumoral stroma regions, rather than in necrotic tissue, are predictive of outcomes [23,24]. No public dataset with tissue annotations or public model capable of segmenting melanoma tumor and necrotic tissue regions exists at this moment.

Furthermore, other immune cell subsets might also have prognostic implications. For example, neutrophils are associated with an increased chance of primary melanoma metastasizing, and B cell presence is associated with response to immune checkpoint inhibition therapy in melanoma [25,26]. The 2020 MoNuSAC challenge showed that it is possible to segment immune cell subsets in H&E stained histopathology images [27].

Consequently, there is a need for a deep learning model capable of segmenting nuclei of tumor cells and different immune cell subsets in H&E slides of melanoma. In addition, such a model should be capable of segmenting tissue areas which can be used for nuclei localization. To address these needs, we created the Panoptic Segmentation of nUclei and tissue in advanced MelanomA (PUMA) dataset. In this paper, we describe the methodology used to create the dataset. Furthermore, we provide nuclei instance segmentation and tissue semantic segmentation benchmarks as well as a first step in improving nuclei segmentation due to the integration of a tissue and nuclei segmentation.

Data description

The PUMA dataset consists of regions of interest (ROIs) with nuclei and tissue annotations. ROIs originate from H&E stained histological slides of melanoma specimens. The dataset's goal is to facilitate the development of deep learning models capable of segmenting nuclei and tissue. To stimulate the use of the dataset and create novel deep learning models, the dataset will also be used for a medical image analysis challenge hosted on the grand-challenge.org platform (accessible through https://puma.grand-challenge.org/). The models created with the dataset and the PUMA challenge, in turn, can be used for prognostic biomarker generation in melanoma treatment.

The dataset consists of 155 primary and 155 metastatic melanoma manually selected ROIs, scanned at 40× magnification (0.22 µm/px) with a resolution of 1024 × 1024 pixels. For these ROIs, annotations of both tissue and nuclei are supplied, as well as a context ROI of 5120 × 5120 pixels centered around the ROI. Annotations were created by a medical expert (author M.S.) and checked and corrected by a dermatopathologist (author W.B.). All cases were digitized in a large melanoma referral center, however 76 cases are revisions or consultations originating from other treatment hospitals. Annotations are in the .GeoJSON format, making annotations easily visualizable with the opensource pathology image viewer Qupath [28,29].

From the total dataset of 310 ROIs, a training set consisting of 103 primary and 103 metastatic ROI has been made publicly available [30]. The remaining 104 ROIs are not publicly available as they are used in the PUMA challenge. In this challenge 10 ROIs are used a preliminary test set for testing of functioning of models. The remaining 94 samples are used as an independent test set for final metric calculations. To be able to compare results from the PUMA challenge to our paper results in this paper are shown for this independent test set.

The public set consists of 97 429 nuclei in 103 primary melanoma ROIs and 103 metastatic melanoma ROIs. The preliminary test set consists out of 5 primary and 5 metastatic melanoma ROIs with a total of 4.860 nuclei. The final test set, consisting of the remaining 47 primary and 47 metastatic samples contains 45.406 nuclei. The distribution of nuclei types and metastatic sample location is visualized in Figure 2.

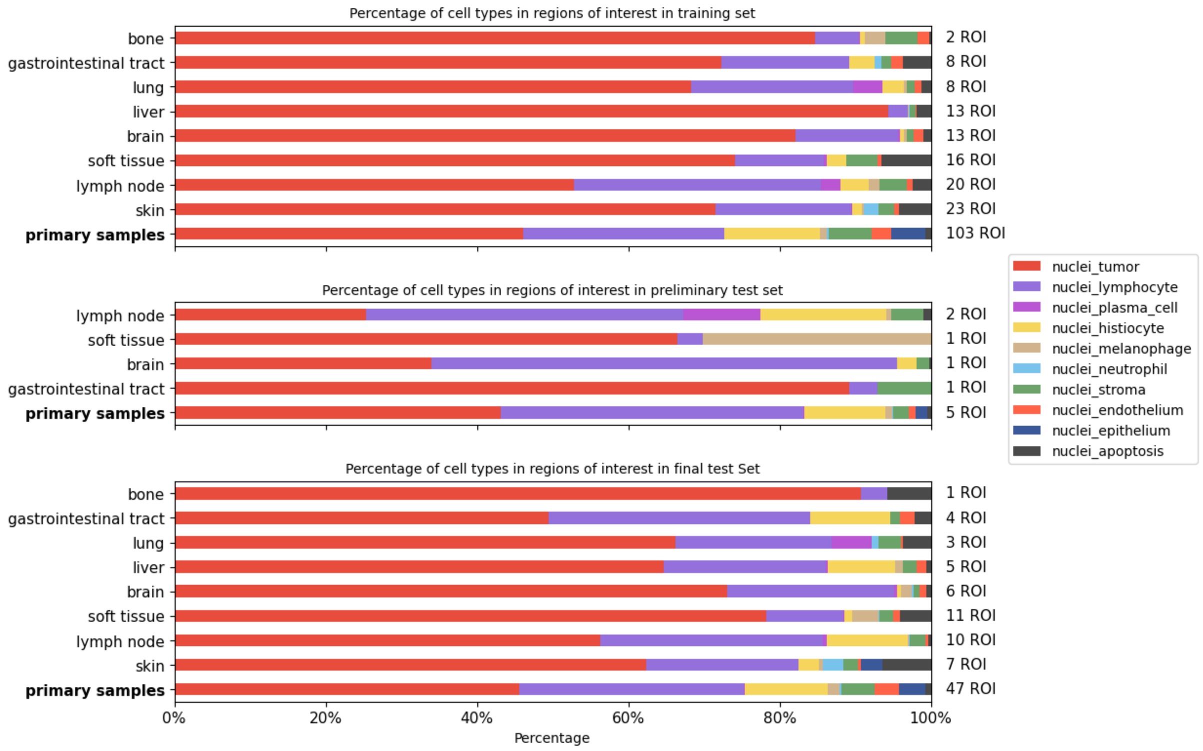

Figure 2. Distribution of nuclei and sampled tissue in the PUMA dataset for primary melanoma regions of interest and metastatic melanoma regions of interest stratified according to metastasis location. The primary samples are depicted as the last row of each figure graph.

As we published previously, more lymphocytes are present in primary samples than in metastatic samples [6]. The most common metastatic lesion sites are lymph nodes and skin metastases. In all samples, lymphocytes and tumor nuclei form the majority of nuclei.

The tissue distribution for the training, preliminary and final test set is visualized in Figure 3. In primary samples, more tumoral stroma is present compared to metastatic samples. The epidermis and necrotic area are underrepresented in both datasets.

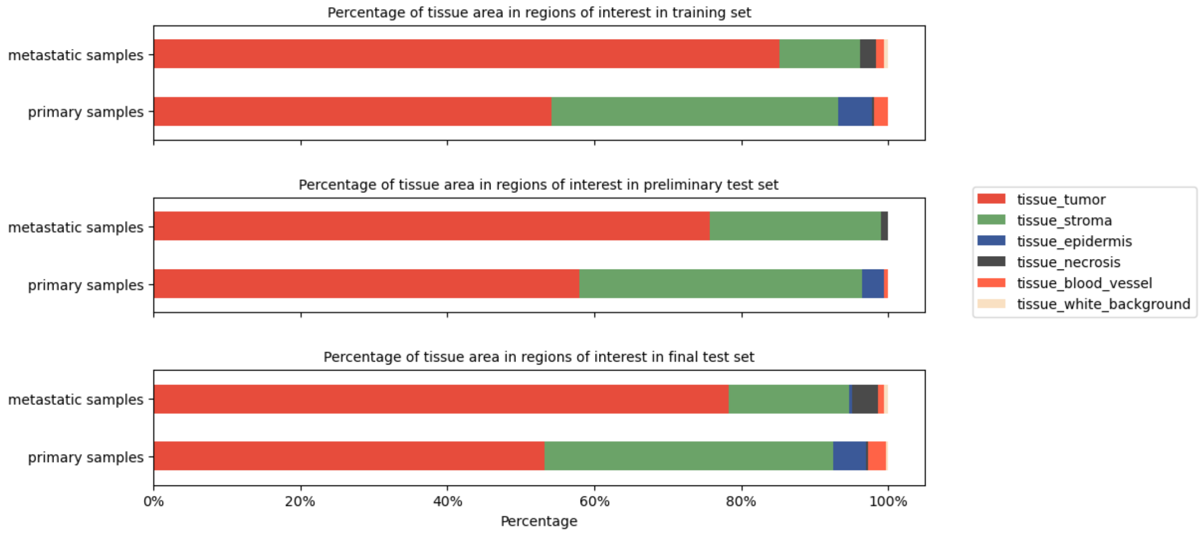

Figure 3. Distribution of tissue class area in the PUMA dataset.

A detailed description of the dataset creation process can be found in the methods section.

Analyses

To set a baseline for the PUMA dataset, we performed four experiments. The first experiment was semantic segmentation of the tumor, stroma, epidermis, blood vessel, and necrotic tissue classes. The second experiment was nuclei segmentation with three nuclei classes: tumor, lymphocyte, and other. For the third experiment, we performed nuclei segmentation with all nuclei classes: tumor, lymphocyte, plasma cell, histiocyte, melanophage, neutrophil, stroma, endothelium, epithelium, and apoptosis. Finally, we show an incorporation of tissue predictions to update the nuclei predictions as a form of heuristic post-processing.

We used 5-fold cross validation while training and report the results of inference on the 94 ROIs of the final independent test set used in the PUMA challenge. In addition, we report the results of the inter- and intraobserver agreement as performed on 12 random samples.

Tissue segmentation

In the first experiment, semantic segmentation of tissue was performed with a nnU-Net model and a Mask2Former model with the backbone replaced by the UNI pathology foundation model [31–33]. The goal of this analysis was to assess to what extent semantic segmentation of different tissue classes is possible with state-of-the-art segmentation models and to evaluate how this correlates with intra- and interobserver agreement. For evaluation of segmentation, the Dice score was computed for each class per sample and averaged across all samples (referred to as average Dice). Additionally, a Dice score was calculated per class on a concatenated sample. To create this sample, all images were combined along one axis, resulting in a single large image with the width of one image and a length equal to the number of images × the height of one image. This is referred to as the micro Dice.

Dice scores are visualized in Table 1 and a visualization of the segmentation is presented in Figure 4. The nnU-Net model achieved the highest overall Dice scores but could not recognize necrosis in the dataset. While Mask2Former Dice scores were lower, it could detect a small part of the necrosis area. When compared to the DICE score of intra- and interobserver agreement, the Dice scores for both models were low in the stroma, epidermis, blood vessel, and necrosis classes.

Table 1. Average and micro average Dice scores of semantic segmentation of tissue over all samples. Results are displayed for a nnU-Net model and a Mask2Former model with the backbone replaced by the UNI pathology foundation model. For comparison, DICE scores of intra- and interobsever agreement are displayed.

	Class	Tumor [95% CI]	Stroma [95% CI]	Epidermis [95% CI]	Blood Vessel [95% CI]	Necrosis [95% CI]	Average [95% CI]
**nnU-Net**	Average	0.87 [0.83 - 0.90]	0.59 [0.52 - 0.68]	0.91 [0.86 - 0.96]	0.54 [0.45 - 0.63]	0.93 [0.87 - 0.98]	0.77 [0.73 - 0.80]
	Micro Average	0.91 [0.88 - 0.94]	0.78 [0.71 - 0.84]	0.69 [0.42 - 0.86]	0.35 [0.23 - 0.47]	0.01 [0.00 - 0.04]	0.55 [0.54 - 0.55]
**Mask2-Former (UNI backbone)**	Average	0.81 [0.76 - 0.86]	0.47 [0.39 - 0.55]	0.90 [0.85 - 0.96]	0.46 [0.36 - 0.57]	0.92 [0.86 - 0.97]	0.71 [0.68 - 0.75]
	Micro Average	0.86 [0.82 - 0.89]	0.62 [0.52 - 0.71]	0.63 [0.34 - 0.84]	0.01 [0.00 - 0.02]	0.09 [0.00 - 0.23]	0.44 [0.43 – 0.44]
**Intra-observer**	Average	0.96 [0.93 - 0.99]	0.89 [0.72 - 0.99]	1.00 [0.99 - 1.0]	0.86 [0.75 - 0.95]	0.97 [0.92 - 1.0]	0.94 [0.90 - 0.97]
	Micro Average	0.98 [0.95 - 0.99]	0.94 [0.90 - 0.97]	0.98 [0.98 - 1.0]	0.68 [0.52 - 0.79]	0.91 [0.70 - 1.0]	0.90 [0.90 - 0.91]
**Inter-observer**	Average	0.97 [0.95 - 0.98]	0.89 [0.73 - 0.99]	1.00 [0.99 - 1.00]	0.72 [0.50 - 0.9]	0.97 [0.91 - 1.0]	0.91 [0.87 - 0.95]
	Micro Average	0.98 [0.97 - 0.99]	0.94 [0.89 - 0.97]	0.97 [0.96 - 1.0]	0.73 [0.59 - 0.83]	0.90 [0.67 - 1.0]	0.90 [0.90 - 0.90]

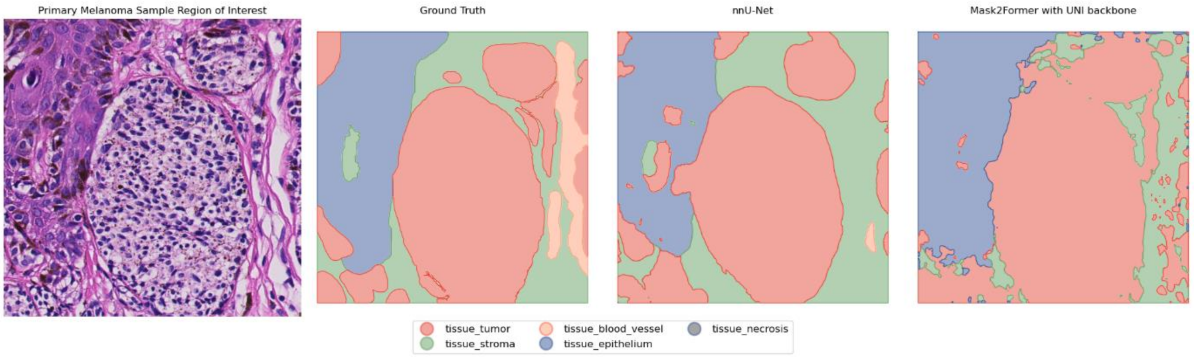

Figure 4. The visual result of semantic segmentation of tissue with the ground truth, the result of the nnU-Net model, and the Mask2Former model.

Segmentation of three nuclei classes

In the second experiment, nuclei segmentation was performed for three classes: tumor nuclei, lymphocytes (including plasma nuclei), and other. The goal of the analysis was to evaluate the usability of existing models in skin and/or melanoma histopathology, compare them with models trained on our dataset, and assess how this correlates with intra- and interobserver agreement. For this experiment we compared the NN192 model, Hover-Net and Hover-NeXt trained on the PanNuke dataset (a pan-tissue dataset that includes skin tissue) and Hover-Net and Hover-NeXt trained on our dataset. Among all evaluated models, the Hover-NeXt model trained on the PUMA dataset using three classes as input for training, demonstrated the highest performance with a F_1 score for lymphocytes close to the intra- and interobserver agreement.

The NN192 model, a melanoma specific model trained to recognize lymphocytes, showed the lowest performance. The Hover-Net and Hover-NeXt models trained on the PanNuke dataset were more successful in detecting lymphocytes. However, the PanNuke-trained Hover-Net model tended to misclassify tumor nuclei as lymphocytes. Models trained on the PUMA dataset, logically, had better performance on evaluation on the test dataset. Results are displayed in Table 2, and an example of the model predictions is shown in Figure 5.

Table 2. F_1 scores for segmentation of tumor, lymphocyte, and other nuclei categories. Results are shown for intra- and interobserver agreement, the NN192 model, Hover-Net and Hover-NeXt trained on the PanNuke dataset and Hover-Net and Hover-NeXt trained on the PUMA dataset.

	**Tumor** [95% CI]	**Lymphocyte** [95% CI]	**Other** [95% CI]	**Micro F_1** [95% CI]	**Average F_1** [95% CI]
**Intraobserver agreement**	0.92 [0.9 - 0.94]	0.85 [0.8 - 0.89]	0.82 [0.77 - 0.86]	0.89 [0.89 - 0.89]	0.86 [0.85 - 0.87]
**Interobserver agreement**	0.89 [0.87 - 0.91]	0.83 [0.79 - 0.88]	0.76 [0.71 - 0.81]	0.85 [0.85 - 0.85]	0.83 [0.82 - 0.84]
**NN192**	0.59 [0.59 - 0.6]	0.11 [0.10 - 0.11]	0.13 [0.12 - 0.14]	0.46 [0.46 - 0.46]	0.28 [0.27 - 0.28]
**Hover-Net (PanNuke)**	0.74 [0.74 - 0.75]	0.57 [0.55 - 0.58]	0.37 [0.36 - 0.38]	0.64 [0.64 - 0.64]	0.56 [0.56 - 0.56]
**Hover-NeXt (PanNuke)**	0.68 [0.67 - 0.68]	0.68 [0.67 - 0.70]	0.38 [0.37 - 0.39]	0.61 [0.61 - 0.61]	0.58 [0.58 - 0.58]
**Hover-Net (PUMA)**	0.74 [0.73 - 0.74]	0.69 [0.68 - 0.69]	0.41 [0.41 - 0.42]	0.66 [0.66 - 0.66]	0.61 [0.61 - 0.61]
**Hover-NeXt (PUMA)**	0.81 [0.81 - 0.81]	0.80 [0.79 - 0.81]	0.50 [0.49 - 0.51]	0.76 [0.76 - 0.76]	0.70 [0.70 - 0.71]

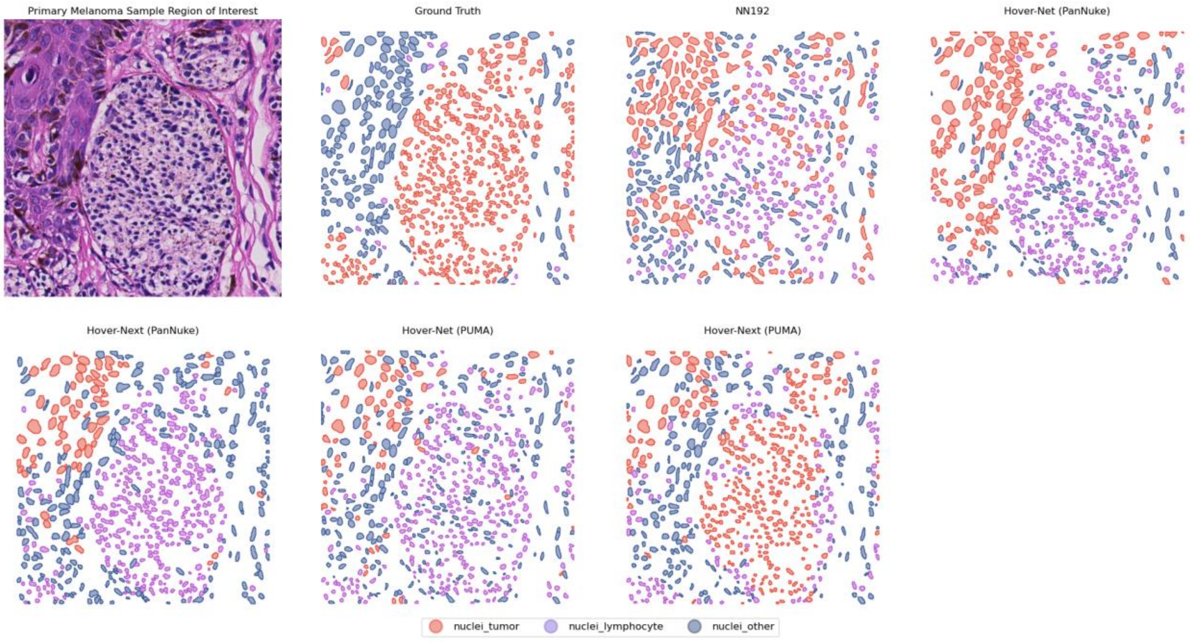

Figure 5. Visual results of segmentation of tumor nuclei, lymphocytes and other nuclei. Results are shown for the NN192 model, the Hover-Net and Hover-NeXt model trained on the PanNuke dataset and the Hover-Net and Hover-NeXt model trained on the PUMA dataset.

Classification of all nuclei classes

In the third experiment, segmentation was performed for all nuclei classes within the PUMA dataset using Hover-Net and Hover-NeXt. In this analysis, we evaluated to what extent Hover-Net and Hover-NeXt are able to correctly segment all nuclei classes present in the dataset. In addition, this analysis aimed at establishing intra- and interobserver agreement for all classes.

Both Hover-Net and Hover-Next showed low performance in terms of segmentation of all non-common classes (Table 3). In addition, the F_1 scores for Tumor and Lymphocyte were lower when compared to the same models trained with three nuclei classes. In the visual representation (Figure 5) the decreased capacity of the Hover-NeXt model of segmenting difficult cases is clearly visible.

Inter- and intraobserver F_1 scores are lower due to disagreement in the classification of plasma cells, melanophages, stroma nuclei, and endothelium. In the 12 randomly selected samples used to assess intra- and interobserver agreement, plasma cells and melanophages had a low incidence; 2 plasma cells, and 14 melanophages were present (Table 4). Stroma, endothelium, and histiocytes had higher incidence but were still subject to substantial disagreement.

Table 3. F_1 scores for intra- and interobserver agreement and model segmentation in all classes.

	**Tumor**	**Lymphocyte**	**Plasma cell**	**Histiocyte**	**Melanophage**	**Neutrophil**
**Intra observer**	0.92 [0.90 - 0.94]	0.85 [0.80 - 0.89]	0.0 [0.0 - 0.0]	0.60 [0.53 - 0.67]	0.59 [0.26 - 0.97]	0.77 [0.61 - 0.93]
**Inter Observer**	0.89 [0.87 - 0.91]	0.83 [0.79 - 0.88]	0.0 [0.0 - 0.0]	0.44 [0.37 - 0.51]	0.28 [0.00 - 0.64]	0.68 [0.50 - 0.85]
**Hover-Net (PUMA)**	0.73 [0.72 - 0.73]	0.67 [0.66 - 0.67]	0.07 [0.06 - 0.09]	0.27 [0.26 - 0.27]	0.31 [0.29 - 0.33]	0.15 [0.13 - 0.18]
**Hover-NeXt (PUMA)**	0.70 [0.70 - 0.71]	0.71 [0.70 - 0.71]	0.10 [0.09 - 0.11]	0.33 [0.32 - 0.34]	0.26 [0.25 - 0.28]	0.06 [0.03 - 0.08]
	**Stroma**	**Endothelium**	**Epithelium**	**Apoptosis**	**Micro F_1**	**Average F_1**
**Intra observer**	0.51 [0.37 - 0.64]	0.71 [0.59 - 0.84]	0.92 [0.77 - 1.07]	0.68 [0.57 - 0.79]	0.86 [0.86 - 0.86]	0.65 [0.61 - 0.70]
**Inter Observer**	0.34 [0.27 - 0.41]	0.45 [0.34 - 0.57]	0.91 [0.77 - 1.05]	0.60 [0.51 - 0.70]	0.80 [0.80 - 0.8]	0.54 [0.50 - 0.58]
**Hover-Net (PUMA)**	0.31 [0.29 - 0.32]	0.15 [0.14 - 0.17]	0.13 [0.12 - 0.14]	0.22 [0.21 - 0.24]	0.61 [0.61 - 0.61]	0.27 [0.27 - 0.28]
**Hover-NeXt (PUMA)**	0.21 [0.20 - 0.22]	0.24 [0.23 - 0.25]	0.04 [0.03 - 0.05]	0.04 [0.04 - 0.05]	0.61 [0.61 - 0.61]	0.27 [0.27 - 0.27]

Table 4. Nuclei count in intra and inter-observer ground truth dataset consisting out of 12 samples.

	Tumor	Lymphocyte	Plasma cell	Histiocyte	Melanophage
**Nuclei count**	3394	1242	2	353	14
	**Neutrophil**	**Stroma**	**Endothelium**	**Epithelium**	**Apoptosis**
**Nuclei count**	103	110	161	173	196

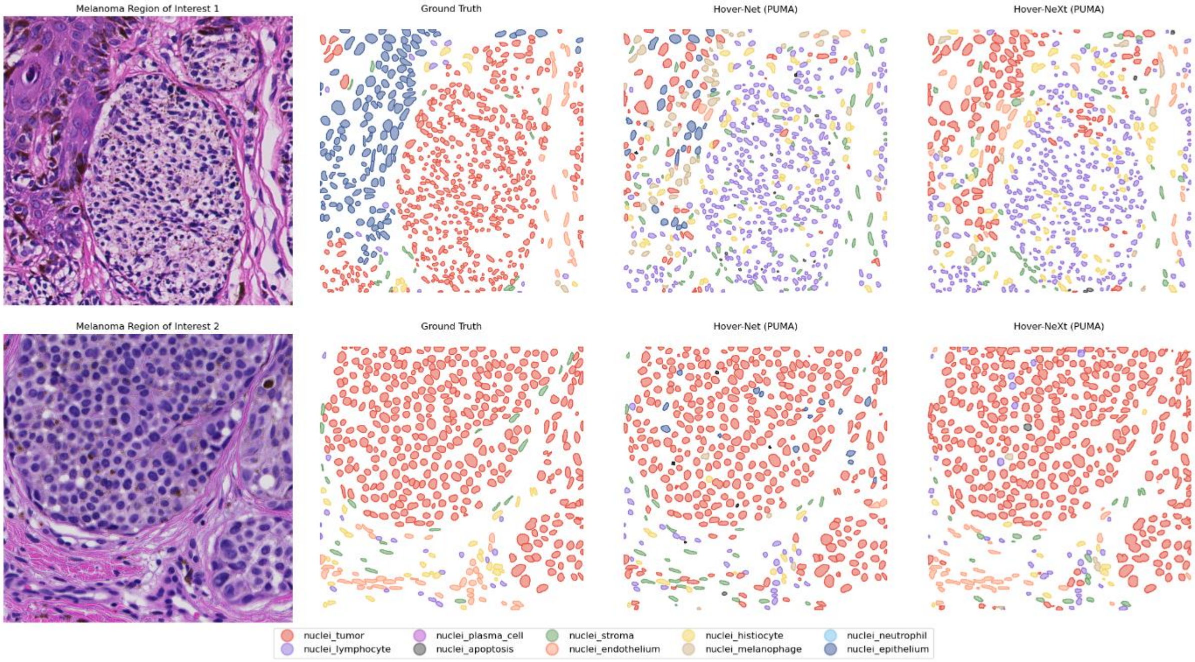

Figure 6. Visual results of segmentation of all nuclei classes. Results are shown for the Hover-Net and Hover-NeXt model trained on the PUMA dataset. In ROI 1 neither of the models is able to classify tumor nuclei and lymphocytes. In ROI 2 model performance is better with identification of tumor nuclei and endothelium nuclei.

Heuristic post processing: combining tissue and nuclei predictions

In the fourth experiment, we used the tissue mask as predicted by the nnU-Net model to improve nuclei segmentation. If a nucleus center was placed inside a tissue mask of the epidermis, the nuclei was classified as an epidermal nuclei. In addition, if a nucleus was present inside the blood vessel class, the nucleus was classified as endothelial nucleus. In Hover-Net and Hover-NeXt, the average F1 score increased from 0.27 and 0.27 to 0.31 and 0.32, respectively. This is mainly due to an increase in the F_1 score for epithelium.

Table 3. F_1 scores for heuristic post processing of the Hover-Net and Hover-NeXt output compared with their non-post processing output.

	**Tumor**	**Lymphocyte**	**Plasma cell**	**Histiocyte**	**Melanophage**	**Neutrophil**
**Hover-Net (PUMA)**	0.73 [0.72 - 0.73]	0.67 [0.66 - 0.67]	0.07 [0.06 - 0.09]	0.27 [0.26 - 0.27]	0.31 [0.29 - 0.33]	0.15 [0.13 - 0.18]
**Hover-Net (PUMA - post-processed)**	0.73 [0.73 - 0.73]	0.67 [0.66 - 0.67]	0.07 [0.05 - 0.08]	0.27 [0.26 - 0.27]	0.32 [0.30 - 0.33]	0.15 [0.12 - 0.17]
**Hover-NeXt (PUMA)**	0.70 [0.70 - 0.71]	0.71 [0.70 - 0.71]	0.10 [0.09 - 0.11]	0.33 [0.32 - 0.34]	0.26 [0.25 - 0.28]	0.06 [0.03 - 0.08]
**Hover-NeXt (PUMA – post processed)**	0.71 [0.71 - 0.71]	0.71 [0.70 - 0.71]	0.1 [0.09 - 0.11]	0.33 [0.32 - 0.34]	0.27 [0.25 - 0.28]	0.06 [0.04 - 0.08]
	**Stroma**	**Endothelium**	**Epithelium**	**Apoptosis**	**Micro F_1**	**Average F_1**
**Hover-Net (PUMA)**	0.31 [0.29 - 0.32]	0.15 [0.14 - 0.17]	0.13 [0.12 - 0.14]	0.22 [0.21 - 0.24]	0.61 [0.61 - 0.61]	0.27 [0.27 - 0.28]
**Hover-Net (PUMA - post-processed)**	0.31 [0.29 - 0.32]	0.2 [0.19 - 0.21]	0.44 [0.42 - 0.46]	0.22 [0.21 - 0.24]	0.62 [0.62 - 0.62]	0.31 [0.30 - 0.31]
**Hover-NeXt (PUMA)**	0.21 [0.20 - 0.22]	0.24 [0.23 - 0.25]	0.04 [0.03 - 0.05]	0.04 [0.04 - 0.05]	0.61 [0.61 - 0.61]	0.27 [0.27 - 0.27]
**Hover-NeXt (PUMA – post processed)**	0.21 [0.20 - 0.22]	0.25 [0.24 - 0.26]	0.52 [0.50 - 0.55]	0.05 [0.04 - 0.06]	0.62 [0.62 - 0.62]	0.32 [0.32 - 0.32]

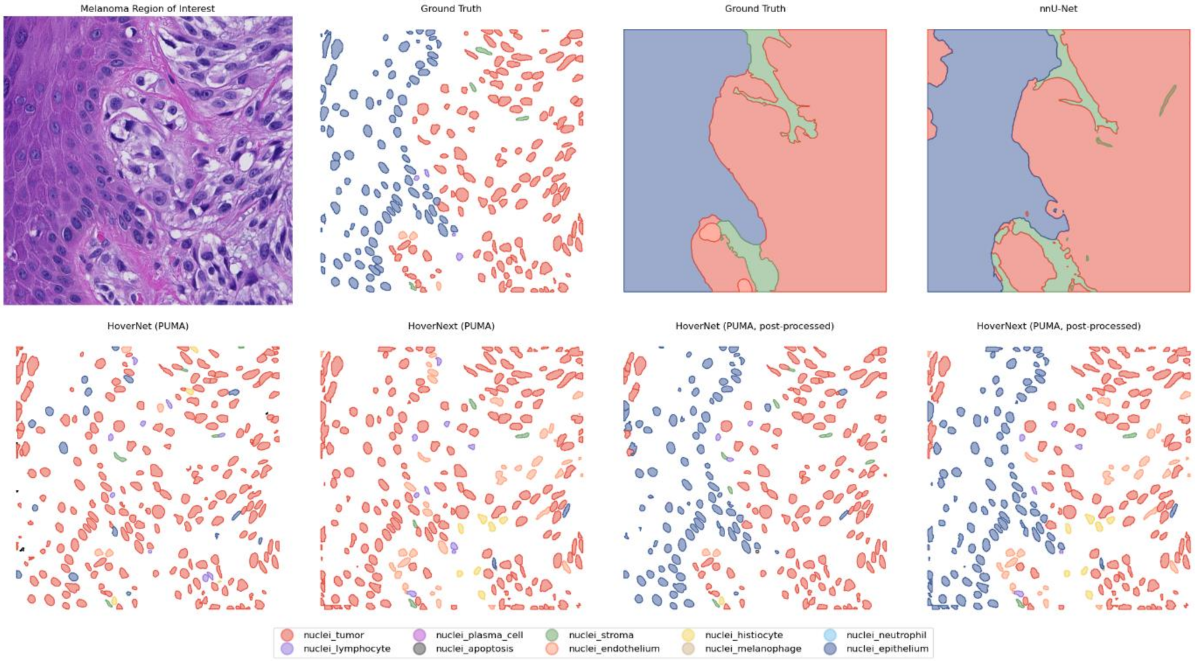

Figure 7. Visual results of heuristic post-processing. For clarification, the ground truth and original predictions of Hover-Net and Hover-NeXt are displayed.

Discussion

In the present study, we show the methodology used to create the PUMA dataset, which is the first melanoma specific nuclei and tissue dataset. In addition, we provide baseline benchmarks in which we show that the Hover-NeXt model trained with three classes (tumor, lymphocyte, and other) can detect lymphocytes almost on human interobserver level performance. Finally, we show an example of the integration of tissue and nuclei annotations leading to improved nuclei segmentation. We believe that further improvement of deep learning models through this integration is possible, and therefore, the dataset will be used to organize the PUMA challenge.

Earlier studies regarding nuclei segmentation have been performed with multiple tissue datasets [17,27] or other tissue specific datasets in, for example, breast cancer [34] and colon cancer [35]. However, thus far, no melanoma specific dataset is available. Since melanocytes and melanoma cells can mimic other cell types, such as lymphocytes and stroma cells, models created with these dataset are not fully applicable for use in melanoma histopathology [13,18]. Models created with this dataset, will be usable for creation of prognostic and/or predictive biomarkers in melanoma histopathology.

The PUMA dataset combines tissue and nuclei segmentation. Earlier studies, such as the OCELOT challenge [36] and the MuTILs model [37] showed that incorporating tissue masks in nuclei prediction increased the capability of models which detect either tumor nuclei or TILs. With this dataset, the goal is to see whether implementation of tissue masks can improve multi class nuclei segmentation for classes such epidermis, endothelium, tumor, and immune cell subsets. In this paper, we demonstrated this by means of a simple post-processing technique. However, other solutions might be passing information from a tissue segmentation model directly to a nuclei segmentation model or making use of multiple sequential models [36,38]. To make use of the creativity and knowledge of the deep learning community, we initiated the PUMA challenge to assess how precise segmentation of melanoma samples can become. Participants in the PUMA challenge can choose to participate in two

tracks. For track 1 the output needs to be nuclei segmentation for three classes (tumor, lymphocyte and other) and tissue segmentation for all classes whereas in track 2 participants need to segment all nuclei classes and tissue segmentation for all classes.

With the dataset, we also aimed to assess the performance of existing models usable in skin and/or melanoma histopathology. Due to unavailability, we were unable to assess the LUNIT scope IO model. However, we were able to evaluate the NN192 algorithm, which is used in multiple studies in which lymphocytes in melanoma histopathology were associated with a decreased chance of recurrence [39] and an increased chance of responding to immune checkpoint inhibition therapy [7]. Surprisingly, the NN192 model showed the lowest capability to detect lymphocytes. This might be due to variations in staining and scanners used in the studies performed earlier and the inability of the techniques used to correct for this. The Hover-Net and Hover-NeXt algorithm architectures can compensate for this by extensive data augmentation in the training (Hover-Net, Hover-NeXt) and inference stages (Hover-NeXt), accounting for better results with the out of the box model trained on the PanNuke dataset. However, both models trained on the PanNuke dataset still showed diminished performance in more difficult to segment melanoma samples when compared to the Hover-NeXt model trained on the PUMA dataset itself.

A strength of this study is the annotation process by a medical expert and an expert dermatopathologist using context ROI to more accurately segment and classify nuclei. In addition, we validated the annotations with a second independent pathologist (GB). From this, it became clear that there is a low interobserver agreement in the less common classes, such as histiocytes, melanophages, and stroma. Melanophages are a subtype of histocytes that have phagocytosed melanin, resulting in a more pigmented appearance. However, there is a continuum between the cells, which could explain the difference in classification. Stroma cells, in addition, are present just like histiocytes in the tissue

around the tumor, and it is not always possible to distinguish them from histiocytes on H&E stained slides.

A second strong point of the study is the manual selection of ROIs. This allowed us to focus on immune cell subsets and less common nuclei and tissue classes. In addition, we tried to include regions with artifacts or much pigmentation. We believe this makes the dataset and the models more applicable to whole slide images, which by nature have artifacts, unsharp regions, and, especially in the case of melanoma, pigmentated more difficult to segment regions.

A downside of the dataset and the study is that all samples are, due to privacy regulations, from one scanner and a single hospital. However, 76 out of 310 cases are consultation cases from referral hospitals already leading to substantial staining variation. In addition, we believe that through stain normalization and data augmentation, models can become less sensible to domain-adversarial effects. This makes models trained on this dataset applicable to whole slide images from other hospitals and scanner types.

Our study did not demonstrate high performance in segmentation of all nuclei classes. This is partly due to the used models losing discriminative ability by introducing more classes. In addition, some classes are less common in the dataset such as apoptosis or histiocytes. Furthermore, there is overlap between classes such as melanophages and histiocytes. We believe that further improvement of models is possible and hope to that through leveraging the knowledge of the deep learning community in the PUMA challenge this will become more clear.

In conclusion, we created a novel dataset with tissue and nuclei segmentations in advanced cutaneous melanoma. With this dataset we showed that deep learning based lymphocyte segmentation can achieve performance levels close to those of human interobserver agreement. In addition, we

demonstrated that it is possible to more accurately segment nuclei classes by incorporating tissue predictions. However, we believe that further improvement is possible by further integration of tissue and nuclei segmentation. Future work in the PUMA challenge will demonstrate to what extent segmentation of different nuclei can be improved.

Potential implications

The PUMA dataset and the deep learning models created with this dataset can be used for biomarker generation in the treatment of melanoma. Thus far, it is known that lymphocytes have predictive value for melanoma recurrence and immune checkpoint inhibition treatment response [6,7,40]. However, this is done either by manual scoring or scoring by automated models, which are not generalizable to new unseen tissue and scanners. With this public dataset and the generalizable deep learning models developed from it, the next step will be to translate these models into clinical application. This will result in more personalized treatment plans, such as de-escalation of immune checkpoint inhibition therapy in patients with advanced melanoma or a less intensive follow-up regimen in patients with primary melanoma. Next to the assessment of TILs as an explainable biomarker, this study will also enable future biomarker discovery studies through the assessment of other nuclei and tissue types.

In addition, this study adds a new step towards further improvement of nuclei segmentation deep learning models in H&E stained histological slides by combining tissue segmentation models into multiclass nuclei segmentation models. This process is closer to how a pathologist evaluates histological slides and already resulted in improved nuclei segmentation benchmark scores.

Methods

Dataset generation

For the dataset, digitized melanoma whole slide H&E stained images generated through regular diagnostic procedures were used from the archive of the UMC Utrecht. All slides were scanned with a Nanozoomer XR C12000–21/− 22 (Hamamatsu Photonics, Hamamatsu, Shizuoka, Japan) at 40× magnification with a resolution of 0.23 µm per pixel. Out of 310 cases in the dataset, 76 are consultation cases from referral hospitals or general practitioners leading to variation in used staining protocols. From each slide, a 40× magnified ROI of 1024×1024 pixels was selected for annotation. In addition, a context ROI of 5120×5120 pixels was sampled to provide information about the broader context for the annotation process. Selection was done by a trained medical expert (M.S.) and subsequently verified by an expert dermatopathologist (W.B.). Manual ROI selection ensured diverse tissue and nuclei types and the inclusion of more difficult to segment areas due to blurring, pigmentation, and scanning artifacts.

Nuclei segmentations were generated with Hover-Net pretrained on the PanNuke dataset [16,17]. Manual annotation adjustment was performed by author M.S. using Qupath with the following nuclei categories: tumor, stroma, vascular endothelium, histiocyte, melanophage, lymphocyte, plasma cell, neutrophil, apoptotic and epithelium [29]. Annotation categories were based on earlier datasets. In addition, we chose categories based on possible predictive value. All annotations were checked and corrected where needed by a dermatopathologist (W.B.). Intra- and inter-observer agreement (by pathologist G.B.) were determined on 12 randomly selected ROIs.

Tissue segmentations were created manually with QuPath by author M.S. using the following categories: tumor, stroma, epidermis, necrosis, blood vessel, and background. Annotation categories are based on the approach used by Hwang et al., who segmented TILs in tumor and stroma areas, and the guidelines from the International Immuno-Oncology Biomarker Working Group [15,41].

Annotations are checked and corrected when needed by a dermatopathologist (W.B.). Intra- and inter-observer agreement (by pathologist G.B.) were determined on 12 randomly selected ROIs to ensure the inclusion of all tissue types.

Benchmark models

Nuclei segmentation

To establish baseline nuclei segmentation benchmarks, we performed nuclei segmentation for two sets of experiments. The first experiment compares models that output three nuclei categories: tumor, lymphocyte, and other. The second experiment is an analysis of the segmentation of all individual nuclei categories. Model training was performed with 5-fold cross validation on the public training dataset without adjusting training parameters or data augmentation of the algorithm used. Inference was performed on the 94 ROIs of the final hidden test set of the PUMA challenge. The remaining 10 ROIs will be used in the PUMA challenge for sanity checking of submitted models.

For the first experiment, the following models were compared: NN192, Hover-Net trained on the PanNuke dataset, Hover-Net trained on the PUMA dataset, Hover-Next trained on the PanNuke dataset, Hover-Next trained on the PUMA dataset [16,17,40,42]. The NN192 model outputs four categories: tumor, lymphocytes, stroma, and other. From this output, the stroma and other categories were merged into one other category. The models trained on the PanNuke dataset classify nuclei into 5 categories: neoplastic, non-neoplastic epithelial, inflammatory, connective, and apoptotic. For the benchmark comparison, neoplastic and inflammatory nuclei were used; non-neoplastic epithelial, connective, and apoptotic were merged into the other category. For training on the PUMA dataset, the plasma cell and lymphocyte categories were merged into a single lymphocyte category, and the remaining classes were combined into the other category. For the second experiment, Hover-Net and Hover-NeXt were trained on all samples and compared.

For the calculation of the F1 score, the center distance between the predicted nuclei and the ground truth nuclei was used. For each ground truth nucleus, predictions within a 15 pixel (3.3 µm) were identified. This radius is smaller than the average size of lymphocytes, which form the smallest nuclei in the dataset. Matching was performed based on the highest predictive score (if available) or the shortest distance. After matching, the ground truth was censored until all ground truth nuclei were either matched or classified as a false negative. Using the identified true positives, false positives, and false negatives, precision (all correct predictions divided by all predictions) and recall (all correct predictions divided by all ground truth nuclei) were calculated. The class F1 score was computed as the harmonic mean of precision and recall, ranging from 0 to 1. Finally to compare models, micro F_1 (aggregation of TP, FP, and FN over all classes, followed by F1 score calculation) and Average F_1 (the average of class F_1 scores) were calculated [43]. Results are shown with a 95% confidence interval which is calculated through bootstrapping the samples.

Tissue Segmentation

For tissue segmentation, nnU-Net and Mask2Former were used to establish baseline benchmarks [31,44]. For training of nnU-Net the same fivefold cross validation was used to create an ensemble model. Mask2Former was pretrained on the COCO instance segmentation task using a Swin Transformer backbone. Images were resized to 512 × 512 pixels before loading into the model. For our experiment we replaced the backbone with the UNI pathology foundation model, which is better able at feature extraction from H&E stained histopathology, after which we finetuned the model on the whole training dataset [32,33,45]. Both models were used for inference on the final hidden test set from the PUMA challenge.

Both models and intra- and interobserver agreement were evaluated using the DICE score. The DICE score is a harmonic mean between 0 and 1, in which 1 is a perfect segmentation prediction, and 0 is no correct prediction. This can result in inflated high average DICE scores if a tissue class is only

present in a few samples, as the DICE score is 1 in the case of a correct absent prediction. To accommodate this, we calculated not only the average DICE score over all samples but also the micro average DICE. This is the DICE score for all predictions concatenated along one axis, resulting in a prediction mask of 1024 × 96.256 pixels. Results are shown with a 95% confidence interval which is generated through bootstrapping the sample results.

Post-processing

For post-processing nuclei, centers were calculated using the Point function from the python Shapely library. From these points, the spatial location inside a tissue mask prediction was determined by using the GeoPandas Python Package. Based on this location the classification label of the nuclei was adjusted.

Availability of source code and requirements

Project name: PUMA

Project home page: https://puma.grand-challenge.org/

Operating system: Platform independent

Programming language: Python 3.10

Other requirements: Hover-Net (available at: https://github.com/vqdang/hover_net), Hover-NeXt (https://github.com/digitalpathologybern/hover_next_train and https://github.com/digitalpathologybern/hover_next_inference) .

nnU-Net (available at: https://github.com/MIC-DKFZ/nnUNet) and Mask2Former (available at https://huggingface.co/docs/transformers/en/model_doc/mask2former).

QuPath 0.5.0 (available at https://qupath.github.io/)

The NN192 classification algorithm (available at https://github.com/acsbal/Automated-TIL-scoring-QuPath-Classifier-for-Melanoma) and QuPath 0.1.2 (https://github.com/qupath/qupath/releases/tag/v0.1.2).

The code and weights for the PUMA challenge inference baseline solutions and metric calculation can be found here: (https://zenodo.org/records/13897135 , https://github.com/tueimage/PUMA-challenge-eval-track1, https://github.com/tueimage/PUMA-challenge-eval-track2, https://github.com/tueimage/PUMA-challenge-baseline-track1, https://github.com/tueimage/PUMA-challenge-baseline-track2)

Qupath scripts to import .geoJSONs for viewing and exporting annotations as masks can be found here: https://zenodo.org/records/13897135.

No changes were made to the training code for the used algorithms.

License: The PUMA codebase is licensed with a CC0 1.0 license (dataset) and the MIT license.

Restrictions to use by non-academics: Both the CC0 1.0 license (dataset) and the MIT license (codebase) allow for non-commercial use. License terms can be reviewed for details.

Data Availability

The PUMA training dataset is available on Zenodo at: https://zenodo.org/records/10940194. Joining the PUMA challenge is possible through the grand challenge platform at https://puma.grand-challenge.org/. The preliminary and final test set will stay hidden until October 10th 2029 due the PUMA challenge still being open for submissions until then. Readers can request earlier access to the hidden dataset for educational or collaborative purposes by contacting the corresponding author via email.

List of abbreviations

TILs: Tumor infiltrating lymphocytes

H&E: Hematoxylin and Eosin

ROI: Region of interest

Declaration

The Biobank Research Ethics Committee (TCBio) UMC Utrecht confirms that it has reviewed the release file in accordance with the UMC Utrecht Biobank Regulations and all other applicable regulations and laws. Based on the requirements as defined in these regulations and laws, the TCBio UMC Utrecht hereby issues an approval of the aforementioned dataset (reference number TCBio 23-270/U-B).

Competing interests

The authors declare the following financial interests/personal relationships which may be considered as potential competing interests:

Karijn P.M. Suijkerbuijk reports a consulting/advisory relationship with Abbvie and Sairopa. She received honoraria from Bristol Myers Squibb and research funding from TigaTx, Bristol Myers Squibb, Philips, Genmab and Pierre Fabre. All paid to institution

The remaining authors of this manuscript have no conflicts of interest to disclose.

Funding

This research was funded by an unrestricted grant of Stichting Hanarth Fonds, The Netherlands.

Authors' contributions

Conceptualization: Schuiveling, Blokx, Suijkerbuijk, Veta

Methodology: Schuiveling, Blokx, Veta, Liu, Breimer

Software: Schuiveling, Eek, Liu

Investigation: Schuiveling, Liu

Resources: Blokx, Suijkerbuijk, Veta

Data Curation: Schuiveling, Blokx, Breimer

Writing, Original Draft: Schuiveling, Liu

Writing, Review & Editing: Blokx, Suijkerbuijk, Veta, Eek, Breimer

Supervision: Blokx, Suijkerbuijk, Veta

Project administration: Blokx, Suijkerbuijk, Veta

Funding acquisition: Suijkerbuijk, Blokx, Veta

References

1. Rahib L, Wehner MR, Matrisian LM, Nead KT. Estimated Projection of US Cancer Incidence and Death to 2040. *JAMA Netw Open*. 2021; doi: 10.1001/jamanetworkopen.2021.4708.

2. Postow MA, Sidlow R, Hellmann MD. Immune-Related Adverse Events Associated with Immune Checkpoint Blockade. *N Engl J Med*. 2018; doi: 10.1056/NEJMra1703481.

3. van Not OJ, Blokx WAM, van den Eertwegh AJM, de Meza MM, Haanen JB, Blank CU, et al.. BRAF and NRAS Mutation Status and Response to Checkpoint Inhibition in Advanced Melanoma. *JCO Precis Oncol*. 2022; doi: 10.1200/PO.22.00018.

4. Franken MG, Leeneman B, Aarts MJB, van Akkooi ACJ, van den Berkmortel FWPJ, Boers-Sonderen MJ, et al.. Trends in survival and costs in metastatic melanoma in the era of novel targeted and immunotherapeutic drugs. *ESMO Open*. 2021; doi: 10.1016/j.esmoop.2021.100320.

5. Suijkerbuijk KPM, van Eijs MJM, van Wijk F, Eggermont AMM. Clinical and translational attributes of immune-related adverse events. *Nat Cancer*. 2024; doi: 10.1038/s43018-024-00730-3.

6. van Duin IAJ, Schuiveling M, ter Maat LS, van Amsterdam WAC, van den Berkmortel F, Boers-Sonderen M, et al.. Baseline tumor-infiltrating lymphocyte patterns and response to immune checkpoint inhibition in metastatic cutaneous melanoma. *European Journal of Cancer*. 2024; doi: 10.1016/j.ejca.2024.114190.

7. Chatziioannou E, Roßner J, Aung TN, Rimm DL, Niessner H, Keim U, et al.. Deep learning-based scoring of tumour-infiltrating lymphocytes is prognostic in primary melanoma and predictive to PD-1 checkpoint inhibition in melanoma metastases. *eBioMedicine*. Elsevier; 2023; doi: 10.1016/j.ebiom.2023.104644.

8. Fu Q, Chen N, Ge C, Li R, Li Z, Zeng B, et al.. Prognostic value of tumor-infiltrating lymphocytes in melanoma: a systematic review and meta-analysis. *Oncoimmunology*. 2019; doi: 10.1080/2162402X.2019.1593806.

9. Clark WH, Elder DE, Guerry D, Braitman LE, Trock BJ, Schultz D, et al.. Model predicting survival in stage I melanoma based on tumor progression. *J Natl Cancer Inst*. 1989; doi: 10.1093/jnci/81.24.1893.

10. Hendry S, Salgado R, Gevaert T, Russell PA, John T, Thapa B, et al.. Assessing Tumor-Infiltrating Lymphocytes in Solid Tumors: A Practical Review for Pathologists and Proposal for a Standardized Method from the International Immuno-Oncology Biomarkers Working Group: Part 2: TILs in Melanoma, Gastrointestinal Tract Carcinomas, Non-Small Cell Lung Carcinoma and Mesothelioma, Endometrial and Ovarian Carcinomas, Squamous Cell Carcinoma of the Head and Neck, Genitourinary Carcinomas, and Primary Brain Tumors. *Adv Anat Pathol*. 2017; doi: 10.1097/PAP.0000000000000161.

11. Busam KJ, Antonescu CR, Marghoob AA, Nehal KS, Sachs DL, Shia J, et al.. Histologic classification of tumor-infiltrating lymphocytes in primary cutaneous malignant melanoma. A study of interobserver agreement. *Am J Clin Pathol*. 2001; doi: 10.1309/G6EK-Y6EH-0LGY-6D6P.

12. Swisher SK, Wu Y, Castaneda CA, Lyons GR, Yang F, Tapia C, et al.. Interobserver Agreement Between Pathologists Assessing Tumor-Infiltrating Lymphocytes (TILs) in Breast Cancer Using

Methodology Proposed by the International TILs Working Group. *Ann Surg Oncol*. 2016; doi: 10.1245/s10434-016-5173-8.

13. Schuiveling M, Blokx WAM, Breimer GE, Suijkerbuijk KPM, Eek D, Veta M. A Novel Dataset for Nuclei Segmentation in Melanoma Histopathology. 2024;

14. : Inflamed immune phenotype predicts favorable clinical outcomes of immune checkpoint inhibitor therapy across multiple cancer types | Journal for ImmunoTherapy of Cancer. https://jitc.bmj.com/content/12/2/e008339 Accessed 2024 Apr 18.

15. Hwang S, Ryu HJ, Kim Y, Cho SI, Oum C, Valero Puche A, et al.. Immune phenotype profiling based on anatomic origin of melanoma and impact on clinical outcomes of immune checkpoint inhibitor treatment. *JCO*. 2024; doi: 10.1200/JCO.2024.42.16_suppl.9569.

16. Graham S, Vu QD, Raza SEA, Azam A, Tsang YW, Kwak JT, et al.. Hover-Net: Simultaneous segmentation and classification of nuclei in multi-tissue histology images. *Medical Image Analysis*. 2019; doi: 10.1016/j.media.2019.101563.

17. Gamper J, Koohbanani NA, Benes K, Graham S, Jahanifar M, Khurram SA, et al.. PanNuke Dataset Extension, Insights and Baselines. arXiv;

18. Ronen S, Czaja RC, Ronen N, Pantazis CG, Iczkowski KA. Small Cell Variant of Metastatic Melanoma: A Mimicker of Lymphoblastic Leukemia/Lymphoma. *Dermatopathology*. 2019; doi: 10.1159/000503703.

19. Magro CM, Crowson AN, Mihm MC. Unusual variants of malignant melanoma. *Mod Pathol*. 2006; doi: 10.1038/modpathol.3800516.

20. Mahbod A, Polak C, Feldmann K, Khan R, Gelles K, Dorffner G, et al.. NuInsSeg: A fully annotated dataset for nuclei instance segmentation in H&E-stained histological images. *Sci Data*. Nature Publishing Group; 2024; doi: 10.1038/s41597-024-03117-2.

21. Mahbod A, Schaefer G, Bancher B, Löw C, Dorffner G, Ecker R, et al.. CryoNuSeg: A dataset for nuclei instance segmentation of cryosectioned H&E-stained histological images. *Computers in Biology and Medicine*. 2021; doi: 10.1016/j.compbiomed.2021.104349.

22. Komura D, Onoyama T, Shinbo K, Odaka H, Hayakawa M, Ochi M, et al.. Restaining-based annotation for cancer histology segmentation to overcome annotation-related limitations among pathologists. *Patterns*. 2023; doi: 10.1016/j.patter.2023.100688.

23. Park S, Ock C-Y, Kim H, Pereira S, Park S, Ma M, et al.. Artificial Intelligence–Powered Spatial Analysis of Tumor-Infiltrating Lymphocytes as Complementary Biomarker for Immune Checkpoint Inhibition in Non–Small-Cell Lung Cancer. *JCO*. Wolters Kluwer; 2022; doi: 10.1200/JCO.21.02010.

24. Choi S, Cho SI, Jung W, Lee T, Choi SJ, Song S, et al.. Deep learning model improves tumor-infiltrating lymphocyte evaluation and therapeutic response prediction in breast cancer. *npj Breast Cancer*. Nature Publishing Group; 2023; doi: 10.1038/s41523-023-00577-4.

25. Helmink BA, Reddy SM, Gao J, Zhang S, Basar R, Thakur R, et al.. B cells and tertiary lymphoid structures promote immunotherapy response. *Nature*. Nature Publishing Group; 2020; doi: 10.1038/s41586-019-1922-8.

26. Landsberg J, Tüting T, Barnhill RL, Lugassy C. The Role of Neutrophilic Inflammation, Angiotropism, and Pericytic Mimicry in Melanoma Progression and Metastasis. *Journal of Investigative Dermatology*. 2016; doi: 10.1016/j.jid.2015.11.013.

27. : MoNuSAC2020: A Multi-Organ Nuclei Segmentation and Classification Challenge | IEEE Journals & Magazine | IEEE Xplore. https://ieeexplore.ieee.org/abstract/document/9446924?casa_token=RVD5ZjFPUJwAAAAA:WqR-dWkLchFwwdtKJGo7GQwwylvOOL6cM796VDb6CDnprbLCYtcm-oMp-0W3MfHiub-Ot0Ab-g Accessed 2024 Jan 8.

28. Butler H, Daly M, Doyle A, Gillies S, Schaub T, Hagen S. The GeoJSON Format. Internet Engineering Task Force; 2016 Aug. Report No.: RFC 7946.

29. : QuPath: Open source software for digital pathology image analysis | Scientific Reports. https://www.nature.com/articles/s41598-017-17204-5 Accessed 2023 Sep 1.

30. Schuiveling M. Melanoma Histopathology Dataset with Tissue and Nuclei Annotations. Zenodo; 2024; doi: 10.5281/zenodo.10940193.

31. Isensee F, Jaeger PF, Kohl SAA, Petersen J, Maier-Hein KH. nnU-Net: a self-configuring method for deep learning-based biomedical image segmentation. *Nat Methods*. Nature Publishing Group; 2021; doi: 10.1038/s41592-020-01008-z.

32. : Towards a general-purpose foundation model for computational pathology | Nature Medicine. https://www.nature.com/articles/s41591-024-02857-3 Accessed 2024 Sep 23.

33. Cheng B, Misra I, Schwing AG, Kirillov A, Girdhar R. Masked-attention Mask Transformer for Universal Image Segmentation. arXiv;

34. : NuCLS: A scalable crowdsourcing approach and dataset for nucleus classification and segmentation in breast cancer | GigaScience | Oxford Academic. https://academic.oup.com/gigascience/article/doi/10.1093/gigascience/giac037/6586817 Accessed 2023 Sep 4.

35. Graham S, Jahanifar M, Azam A, Nimir M, Tsang Y-W, Dodd K, et al.. Lizard: A Large-Scale Dataset for Colonic Nuclear Instance Segmentation and Classification. *2021 IEEE/CVF International Conference on Computer Vision Workshops (ICCVW)*. Montreal, BC, Canada: IEEE;

36. Ryu J, Puche AV, Shin J, Park S, Brattoli B, Lee J, et al.. OCELOT: Overlapped Cell on Tissue Dataset for Histopathology. p. 23902–12.

37. Liu S, Amgad M, More D, Rathore MA, Salgado R, Cooper LAD. A panoptic segmentation dataset and deep-learning approach for explainable scoring of tumor-infiltrating lymphocytes. *npj Breast Cancer*. Nature Publishing Group; 2024; doi: 10.1038/s41523-024-00663-1.

38. Amgad M, Salgado R, Cooper LAD. MuTILs: A multiresolution deep-learning model for interpretable scoring of Tumor-Infiltrating Lymphocytes in breast carcinomas using clinical guidelines. medRxiv;

39. Aung TN, Shafi S, Wilmott JS, Nourmohammadi S, Vathiotis I, Gavrielatou N, et al.. Objective assessment of tumor infiltrating lymphocytes as a prognostic marker in melanoma using machine learning algorithms. *eBioMedicine*. Elsevier; 2022; doi: 10.1016/j.ebiom.2022.104143.

40. Acs B, Ahmed FS, Gupta S, Wong PF, Gartrell RD, Sarin Pradhan J, et al.. An open source automated tumor infiltrating lymphocyte algorithm for prognosis in melanoma. *Nat Commun*. 2019; doi: 10.1038/s41467-019-13043-2.

41. Thagaard J, Broeckx G, Page DB, Jahangir CA, Verbandt S, Kos Z, et al.. Pitfalls in machine learning-based assessment of tumor-infiltrating lymphocytes in breast cancer: A report of the International Immuno-Oncology Biomarker Working Group on Breast Cancer. *J Pathol*. 2023; doi: 10.1002/path.6155.

42. Baumann E, Dislich B, Rumberger JL, Nagtegaal ID, Martınez MR, Zlobec I. HoVer-NeXt: A Fast Nuclei Segmentation and Classification Pipeline for Next Generation Histopathology. *Medical Imaging with Deep Learning*. 2024;

43. Maier-Hein L, Reinke A, Godau P, Tizabi MD, Buettner F, Christodoulou E, et al.. Metrics reloaded: recommendations for image analysis validation. *Nat Methods*. Nature Publishing Group; 2024; doi: 10.1038/s41592-023-02151-z.

44. Cao H, Wang Y, Chen J, Jiang D, Zhang X, Tian Q, et al.. Swin-Unet: Unet-Like Pure Transformer for Medical Image Segmentation. In: Karlinsky L, Michaeli T, Nishino K, editors. *Computer Vision – ECCV 2022 Workshops*. Cham: Springer Nature Switzerland;

45. Lin T-Y, Maire M, Belongie S, Bourdev L, Girshick R, Hays J, et al.: Microsoft COCO: Common Objects in Context. arXiv.org. https://arxiv.org/abs/1405.0312v3 (2014). Accessed 2024 Sep 6.

Manuscript

Click here to access/download;Manuscript;A Novel Dataset for Nuclei and Tissue with tracked changes.docx

A novel dataset for nuclei and tissue segmentation in melanoma with baseline nuclei segmentation and tissue segmentation benchmarks

Mark Schuiveling, MD[1]; Hong Liu[2]; Daniel Eek[3]; Gerben E. Breimer, MD, PhD [4]; Karijn P.M. Suijkerbuijk[5], MD, PhD; Willeke A.M. Blokx[6*], MD, PhD; Mitko Veta, PhD[7*]

Affiliations

1. Department of Medical Oncology, University Medical Center Utrecht, Utrecht University, Heidelberglaan 100, 3584 CG Utrecht, the Netherlands. m.schuiveling@umcutrecht.nl
2. Medical Image Analysis, Department of Biomedical Engineering, Eindhoven University of Technology, Eindhoven, the Netherlands. h.liu2@tue.nl
3. Medical Image Analysis, Department of Biomedical Engineering, Eindhoven University of Technology, Eindhoven, the Netherlands. d.b.eek@student.tue.nl
4. Department of Pathology, University Medical Center Utrecht, Utrecht University, Heidelberglaan 100, 3584 CG Utrecht, the Netherlands. g.e.breimer-2@umcutrecht.nl
5. Department of Medical Oncology, University Medical Center Utrecht, Utrecht University, Heidelberglaan 100, 3584 CG Utrecht, the Netherlands. k.suijkerbuik@umcutrecht.nl
6. Department of Pathology, University Medical Center Utrecht, Utrecht University, Heidelberglaan 100, 3584 CG Utrecht, the Netherlands. w.a.m.blokx@umcutrecht.nl
7. Medical Image Analysis, Department of Biomedical Engineering, Eindhoven University of Technology, Eindhoven, the Netherlands. m.veta@tue.nl

* These authors contributed equally to this work.

Corresponding author: Mark Schuiveling, MD, Department of Medical Oncology, University Medical Center Utrecht, Utrecht University, Postbus 85500, 3508 GA Utrecht, the Netherlands m.schuiveling@umcutrecht.nl)

Abstract

Background
Melanoma is an aggressive form of skin cancer in which tumor-infiltrating lymphocytes (TILs) are a biomarker for recurrence and treatment response. Manual TIL assessment is prone to interobserver variability, and current deep learning models are not publicly accessible or have low performance. Deep learning models, however, have the potential of consistent spatial evaluation of TILs and other immune cell subsets with the potential of improved prognostic and predictive value. To make the development of these models possible, we created the Panoptic Segmentation of nUclei and tissue in advanced MelanomA (PUMA) dataset and assessed the performance of several state-of-the-art deep learning models. In addition, we show how to improve model performance further by using heuristic post-processing in which nuclei classes are updated based on their tissue localization.

Results
The PUMA dataset includes 155 primary and 155 metastatic melanoma H&E stained regions of interest with nuclei and tissue annotations from a single melanoma referral institution. The Hover-NeXt model, trained on the PUMA dataset, demonstrated the best performance for lymphocyte detection, approaching human interobserver agreement. In addition, heuristic post-processing of deep learning models improve the detection of non-common classes, such as epithelial nuclei.

Conclusion
The PUMA dataset is the first melanoma specific dataset that can be used to develop melanoma-specific nuclei and tissue segmentation models. These models can, in turn, be used for prognostic and predictive biomarker development. Incorporating tissue and nuclei segmentation is a step towards improved deep learning nuclei segmentation performance. To support the development of these models, this dataset is used in the PUMA challenge.

Keywords:

Melanoma, H&E stained histopathology, nuclei segmentation, tissue segmentation, TILs

Background

Melanoma is an aggressive form of skin cancer with increasing incidence [1]. Primary melanoma is treated with surgical excision, whereas advanced, metastasized melanoma is most commonly treated with immune checkpoint inhibition therapy, a form of cancer immunotherapy. However, half of the advanced melanoma patients do not respond to this treatment, which is costly and potentially toxic [2–5].

Previous studies showed that tumor infiltrating lymphocytes (TILs) in hematoxylin & eosin (H&E) stained slides of metastatic melanoma before the start of treatment are associated with a higher chance of response to immune checkpoint inhibition and an increase in survival [6,7]. In addition, TILs are associated with reduced recurrence rates in primary melanoma [8]. Therefore, assessment of TILs can serve as a prognostic biomarker in melanoma treatment.

Currently, TILs are scored manually, either by estimating a stromal percentage or through using a multitier system like the Clark score, which categorizes TILs as absent, non-brisk, or brisk [6,9,10]. However, substantial interobserver variability exists among pathologists [6,11,12]. A deep learning-based assessment could result in a more precise, consistent and fine-grained assessment of TILs.

To date, two deep learning-based models have been used to evaluate TILs in melanoma histopathology. The first model, NN192, uses watershed segmentation followed by a fully connected neural network [7]. However, this older, suboptimal technique results in lower performance [13]. The second model, LUNIT's Scope IO pan tumor model, is not melanoma specific and not publicly available [14,15]. Publicly available models, such as Hover-Net pretrained on the PanNuke dataset (which includes skin samples) can be used to detect TILs in melanoma, but have suboptimal performance. This is due to the model not being melanoma-specific [16,17]. As a result, misclassifications occur because melanoma cells can resemble other cell types, such as stroma or lymphocytes [13,18,19].

As for datasets which can be used to train deep learning models usable in melanoma, the NuInsSeg [20] and CryoNuSeg [21] datasets also partly consist of skin samples. However these datasets do not have separate classes for nuclei such as tumor or lymphocyte rendering them less useful for creating deep learning nuclei classification algorithms. In contract, the SegPath dataset does classify nuclei as lymphocytes using a ground truth based on immunofluorescence staining. However, it contains only one melanoma sample with CD3/CD20 annotations [22].

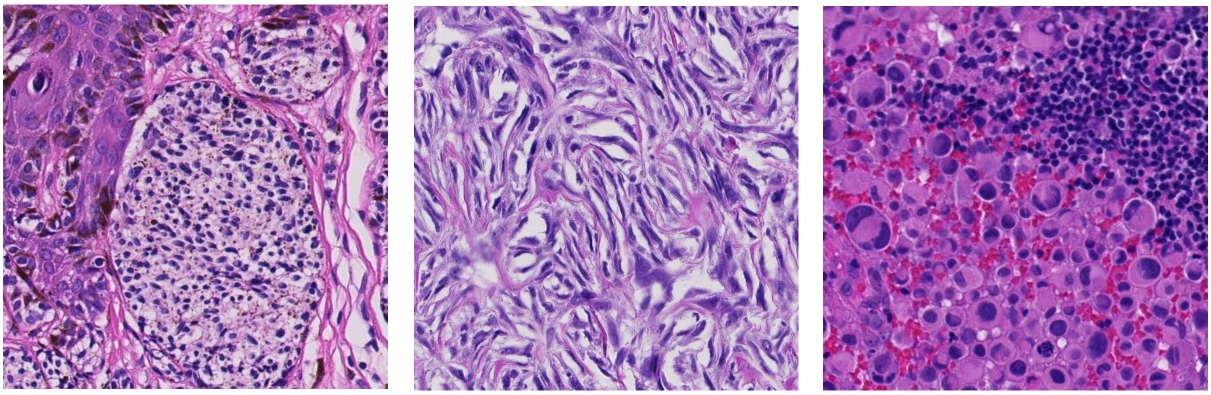

Figure 1. Examples of melanoma histopathology appearances: a lymphocyte-like morphology on the left, a stromal growth pattern in the center, and a tumor with large, variable nuclei on the right.

Next to the presence of (deep learning detected) TILs, the localization of TILs is of importance. In breast cancer and non-small cell lung cancer, TILs located within the tumor or intra-tumoral stroma regions, rather than in necrotic tissue, are predictive of outcomes [23,24]. No public dataset with tissue annotations or public model capable of segmenting melanoma tumor and necrotic tissue regions exists at this moment.

Furthermore, other immune cell subsets might also have prognostic implications. For example, neutrophils are associated with an increased chance of primary melanoma metastasizing, and B cell presence is associated with response to immune checkpoint inhibition therapy in melanoma [25,26]. The 2020 MoNuSAC challenge showed that it is possible to segment immune cell subsets in H&E stained histopathology images [27].

Consequently, there is a need for a deep learning model capable of segmenting nuclei of tumor cells and different immune cell subsets in H&E slides of melanoma. In addition, such a model should be capable of segmenting tissue areas which can be used for nuclei localization. To address these needs, we created the Panoptic Segmentation of nUclei and tissue in advanced MelanomA (PUMA) dataset. In this paper, we describe the methodology used to create the dataset. Furthermore, we provide nuclei instance segmentation and tissue semantic segmentation benchmarks as well as a first step in improving nuclei segmentation due to the integration of a tissue and nuclei segmentation.

Data description

The PUMA dataset consists of regions of interest (ROIs) with nuclei and tissue annotations. ROIs originate from H&E stained histological slides of melanoma specimens. The dataset's goal is to facilitate the development of deep learning models capable of segmenting nuclei and tissue. To stimulate the use of the dataset and create novel deep learning models, the dataset will also be used for a medical image analysis challenge hosted on the grand-challenge.org platform (accessible through https://puma.grand-challenge.org/). The models created with the dataset and the PUMA challenge, in turn, can be used for prognostic biomarker generation in melanoma treatment.

The dataset consists of 155 primary and 155 metastatic melanoma manually selected ROIs, scanned at 40× magnification (0.22 µm/px) with a resolution of 1024 × 1024 pixels. For these ROIs, annotations of both tissue and nuclei are supplied, as well as a context ROI of 5120 × 5120 pixels centered around the ROI. Annotations were created by a medical expert (author M.S.) and checked and corrected by a dermatopathologist (author W.B.). All cases were digitized in a large melanoma referral center, however 76 cases are revisions or consultations originating from other treatment hospitals. Annotations are in the .GeoJSON format, making annotations easily visualizable with the opensource pathology image viewer Qupath [28,29].

From the total dataset of 310 ROIs, a training set consisting of 103 primary and 103 metastatic ROI has been made publicly available [30]. The remaining 104 ROIs are not publicly available as they are used in the PUMA challenge. In this challenge 10 ROIs are used a preliminary test set for testing of functioning of models. The remaining 94 samples are used as an independent test set for final metric calculations. To be able to compare results from the PUMA challenge to our paper results in this paper are shown for this independent test set.

The public set consists of 97 429 nuclei in 103 primary melanoma ROIs and 103 metastatic melanoma ROIs. The preliminary test set consists out of 5 primary and 5 metastatic melanoma ROIs with a total of 4.860 nuclei. The final test set, consisting of the remaining 47 primary and 47 metastatic samples contains 45.406 nuclei. The distribution of nuclei types and metastatic sample location is visualized in Figure 2.

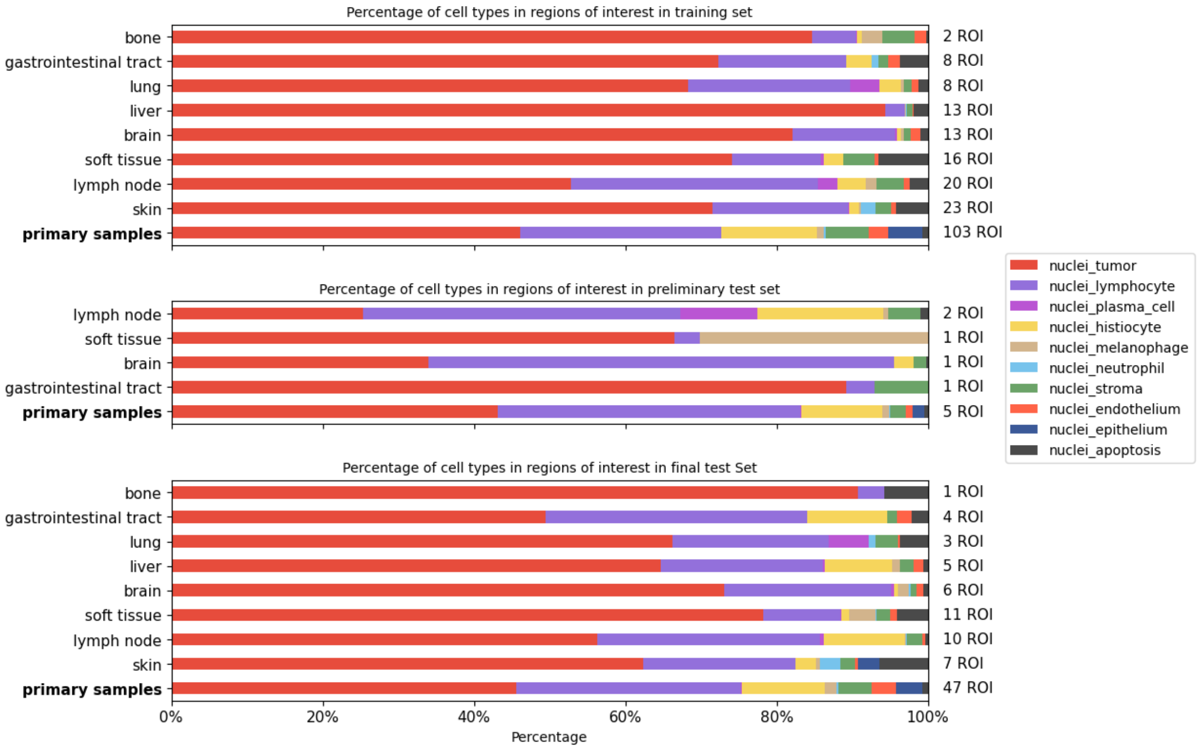

Figure 2. Distribution of nuclei and sampled tissue in the PUMA dataset for primary melanoma regions of interest and metastatic melanoma regions of interest stratified according to metastasis location. The primary samples are depicted as the last row of each figure graph.

As we published previously, more lymphocytes are present in primary samples than in metastatic samples [6]. The most common metastatic lesion sites are lymph nodes and skin metastases. In all samples, lymphocytes and tumor nuclei form the majority of nuclei.

The tissue distribution for the training, preliminary and final test set is visualized in Figure 3. In primary samples, more tumoral stroma is present compared to metastatic samples. The epidermis and necrotic area are underrepresented in both datasets.

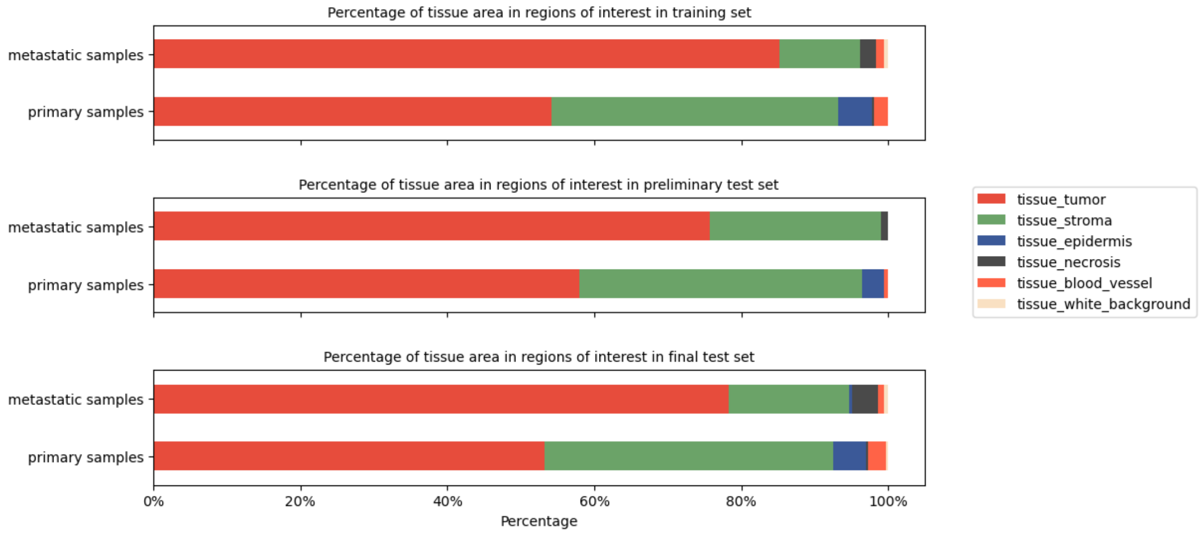

Figure 3. Distribution of tissue class area in the PUMA dataset.

A detailed description of the dataset creation process can be found in the methods section.

Analyses

To set a baseline for the PUMA dataset, we performed four experiments. The first experiment was semantic segmentation of the tumor, stroma, epidermis, blood vessel, and necrotic tissue classes. The second experiment was nuclei segmentation with three nuclei classes: tumor, lymphocyte, and other. For the third experiment, we performed nuclei segmentation with all nuclei classes: tumor, lymphocyte, plasma cell, histiocyte, melanophage, neutrophil, stroma, endothelium, epithelium, and apoptosis. Finally, we show an incorporation of tissue predictions to update the nuclei predictions as a form of heuristic post-processing.

We used 5-fold cross validation while training and report the results of inference on the 94 ROIs of the final independent test set used in the PUMA challenge. In addition, we report the results of the inter- and intraobserver agreement as performed on 12 random samples.

Tissue segmentation

In the first experiment, semantic segmentation of tissue was performed with a nnU-Net model and a Mask2Former model with the backbone replaced by the UNI pathology foundation model [31–33]. The goal of this analysis was to assess to what extent semantic segmentation of different tissue classes is possible with state-of-the-art segmentation models and to evaluate how this correlates with intra- and interobserver agreement. For evaluation of segmentation, the Dice score was computed for each class per sample and averaged across all samples (referred to as average Dice). Additionally, a Dice score was calculated per class on a concatenated sample. To create this sample, all images were combined along one axis, resulting in a single large image with the width of one image and a length equal to the number of images × the height of one image. This is referred to as the micro Dice.

Dice scores are visualized in Table 1 and a visualization of the segmentation is presented in Figure 4. The nnU-Net model achieved the highest overall Dice scores but could not recognize necrosis in the dataset. While Mask2Former Dice scores were lower, it could detect a small part of the necrosis area. When compared to the DICE score of intra- and interobserver agreement, the Dice scores for both models were low in the stroma, epidermis, blood vessel, and necrosis classes.

Table 1. Average and micro average Dice scores of semantic segmentation of tissue over all samples. Results are displayed for a nnU-Net model and a Mask2Former model with the backbone replaced by the UNI pathology foundation model. For comparison, DICE scores of intra- and interobsever agreement are displayed.

	Class	Tumor [95% CI]	Stroma [95% CI]	Epidermis [95% CI]	Blood Vessel [95% CI]	Necrosis [95% CI]	Average [95% CI]
**nnU-Net**	Average	0.87 [0.83 - 0.90]	0.59 [0.52 - 0.68]	0.91 [0.86 - 0.96]	0.54 [0.45 - 0.63]	0.93 [0.87 - 0.98]	0.77 [0.73 - 0.80]
	Micro Average	0.91 [0.88 - 0.94]	0.78 [0.71 - 0.84]	0.69 [0.42 - 0.86]	0.35 [0.23 - 0.47]	0.01 [0.00 - 0.04]	0.55 [0.54 - 0.55]
**Mask2-Former (UNI backbone)**	Average	0.81 [0.76 - 0.86]	0.47 [0.39 - 0.55]	0.90 [0.85 - 0.96]	0.46 [0.36 - 0.57]	0.92 [0.86 - 0.97]	0.71 [0.68 - 0.75]
	Micro Average	0.86 [0.82 - 0.89]	0.62 [0.52 - 0.71]	0.63 [0.34 - 0.84]	0.01 [0.00 - 0.02]	0.09 [0.00 - 0.23]	0.44 [0.43 – 0.44]
**Intra-observer**	Average	0.96 [0.93 - 0.99]	0.89 [0.72 - 0.99]	1.00 [0.99 - 1.0]	0.86 [0.75 - 0.95]	0.97 [0.92 - 1.0]	0.94 [0.90 - 0.97]
	Micro Average	0.98 [0.95 - 0.99]	0.94 [0.90 - 0.97]	0.98 [0.98 - 1.0]	0.68 [0.52 - 0.79]	0.91 [0.70 - 1.0]	0.90 [0.90 - 0.91]
**Inter-observer**	Average	0.97 [0.95 - 0.98]	0.89 [0.73 - 0.99]	1.00 [0.99 - 1.00]	0.72 [0.50 - 0.9]	0.97 [0.91 - 1.0]	0.91 [0.87 - 0.95]
	Micro Average	0.98 [0.97 - 0.99]	0.94 [0.89 - 0.97]	0.97 [0.96 - 1.0]	0.73 [0.59 - 0.83]	0.90 [0.67 - 1.0]	0.90 [0.90 - 0.90]

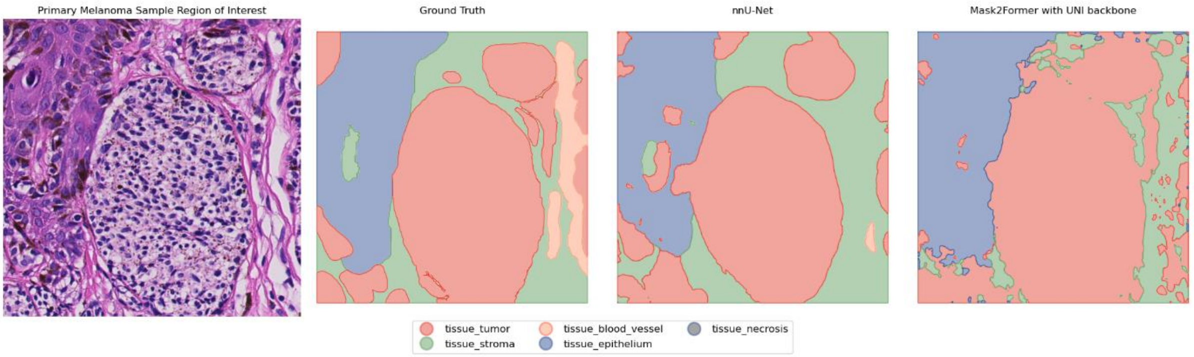

Figure 4. The visual result of semantic segmentation of tissue with the ground truth, the result of the nnU-Net model, and the Mask2Former model.

Segmentation of three nuclei classes

In the second experiment, nuclei segmentation was performed for three classes: tumor nuclei, lymphocytes (including plasma nuclei), and other. The goal of the analysis was to evaluate the usability of existing models in skin and/or melanoma histopathology, compare them with models trained on our dataset, and assess how this correlates with intra- and interobserver agreement. For this experiment we compared the NN192 model, Hover-Net and Hover-NeXt trained on the PanNuke dataset (a pan-tissue dataset that includes skin tissue) and Hover-Net and Hover-NeXt trained on our dataset. Among all evaluated models, the Hover-NeXt model trained on the PUMA dataset using three classes as input for training, demonstrated the highest performance with a F_1 score for lymphocytes close to the intra- and interobserver agreement.

The NN192 model, a melanoma specific model trained to recognize lymphocytes, showed the lowest performance. The Hover-Net and Hover-NeXt models trained on the PanNuke dataset were more successful in detecting lymphocytes. However, the PanNuke-trained Hover-Net model tended to misclassify tumor nuclei as lymphocytes. Models trained on the PUMA dataset, logically, had better performance on evaluation on the test dataset. Results are displayed in Table 2, and an example of the model predictions is shown in Figure 5.

Table 2. F_1 scores for segmentation of tumor, lymphocyte, and other nuclei categories. Results are shown for intra- and interobserver agreement, the NN192 model, Hover-Net and Hover-NeXt trained on the PanNuke dataset and Hover-Net and Hover-NeXt trained on the PUMA dataset.

	**Tumor** [95% CI]	**Lymphocyte** [95% CI]	**Other** [95% CI]	**Micro F_1** [95% CI]	**Average F_1** [95% CI]
**Intraobserver agreement**	0.92 [0.9 - 0.94]	0.85 [0.8 - 0.89]	0.82 [0.77 - 0.86]	0.89 [0.89 - 0.89]	0.86 [0.85 - 0.87]
**Interobserver agreement**	0.89 [0.87 - 0.91]	0.83 [0.79 - 0.88]	0.76 [0.71 - 0.81]	0.85 [0.85 - 0.85]	0.83 [0.82 - 0.84]
**NN192**	0.59 [0.59 - 0.6]	0.11 [0.10 - 0.11]	0.13 [0.12 - 0.14]	0.46 [0.46 - 0.46]	0.28 [0.27 - 0.28]
**Hover-Net (PanNuke)**	0.74 [0.74 - 0.75]	0.57 [0.55 - 0.58]	0.37 [0.36 - 0.38]	0.64 [0.64 - 0.64]	0.56 [0.56 - 0.56]
**Hover-NeXt (PanNuke)**	0.68 [0.67 - 0.68]	0.68 [0.67 - 0.70]	0.38 [0.37 - 0.39]	0.61 [0.61 - 0.61]	0.58 [0.58 - 0.58]
**Hover-Net (PUMA)**	0.74 [0.73 - 0.74]	0.69 [0.68 - 0.69]	0.41 [0.41 - 0.42]	0.66 [0.66 - 0.66]	0.61 [0.61 - 0.61]
**Hover-NeXt (PUMA)**	0.81 [0.81 - 0.81]	0.80 [0.79 - 0.81]	0.50 [0.49 - 0.51]	0.76 [0.76 - 0.76]	0.70 [0.70 - 0.71]

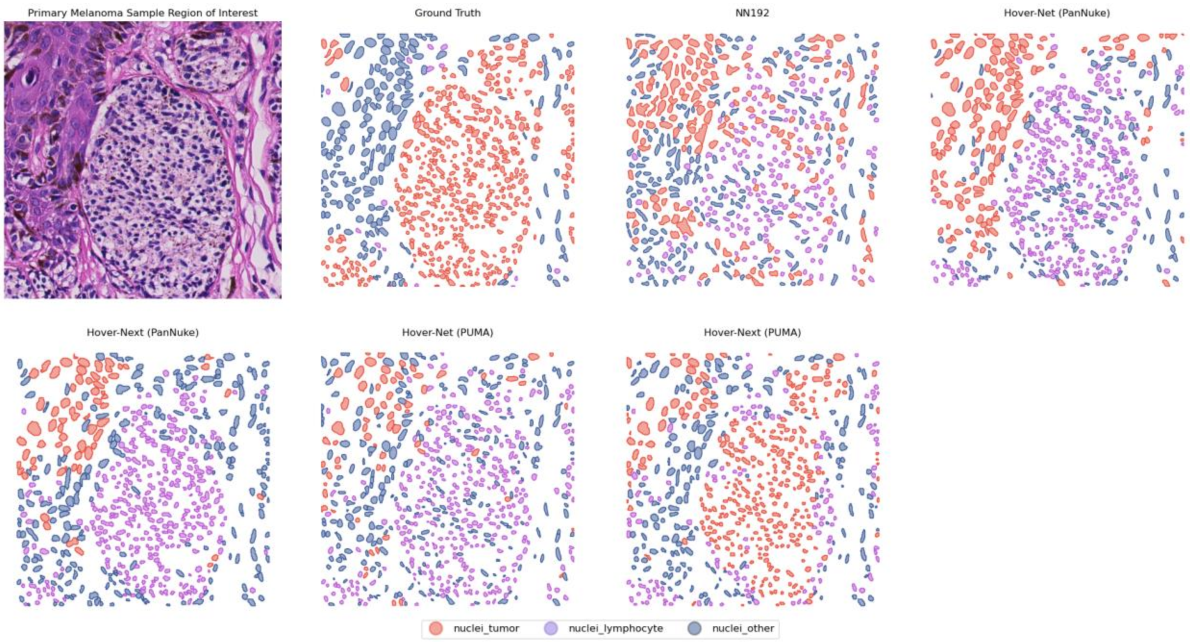

Figure 5. Visual results of segmentation of tumor nuclei, lymphocytes and other nuclei. Results are shown for the NN192 model, the Hover-Net and Hover-NeXt model trained on the PanNuke dataset and the Hover-Net and Hover-NeXt model trained on the PUMA dataset.

Classification of all nuclei classes

In the third experiment, segmentation was performed for all nuclei classes within the PUMA dataset using Hover-Net and Hover-NeXt. In this analysis, we evaluated to what extent Hover-Net and Hover-NeXt are able to correctly segment all nuclei classes present in the dataset. In addition, this analysis aimed at establishing intra- and interobserver agreement for all classes.

Both Hover-Net and Hover-Next showed low performance in terms of segmentation of all non-common classes (Table 3). In addition, the F_1 scores for Tumor and Lymphocyte were lower when compared to the same models trained with three nuclei classes. In the visual representation (Figure 5) the decreased capacity of the Hover-NeXt model of segmenting difficult cases is clearly visible.

Inter- and intraobserver F_1 scores are lower due to disagreement in the classification of plasma cells, melanophages, stroma nuclei, and endothelium. In the 12 randomly selected samples used to assess intra- and interobserver agreement, plasma cells and melanophages had a low incidence; 2 plasma cells, and 14 melanophages were present (Table 4). Stroma, endothelium, and histiocytes had higher incidence but were still subject to substantial disagreement.

Table 3. F_1 scores for intra- and interobserver agreement and model segmentation in all classes.

	**Tumor**	**Lymphocyte**	**Plasma cell**	**Histiocyte**	**Melanophage**	**Neutrophil**
**Intra observer**	0.92 [0.90 - 0.94]	0.85 [0.80 - 0.89]	0.0 [0.0 - 0.0]	0.60 [0.53 - 0.67]	0.59 [0.26 - 0.97]	0.77 [0.61 - 0.93]
**Inter Observer**	0.89 [0.87 - 0.91]	0.83 [0.79 - 0.88]	0.0 [0.0 - 0.0]	0.44 [0.37 - 0.51]	0.28 [0.00 - 0.64]	0.68 [0.50 - 0.85]
**Hover-Net (PUMA)**	0.73 [0.72 - 0.73]	0.67 [0.66 - 0.67]	0.07 [0.06 - 0.09]	0.27 [0.26 - 0.27]	0.31 [0.29 - 0.33]	0.15 [0.13 - 0.18]
**Hover-NeXt (PUMA)**	0.70 [0.70 - 0.71]	0.71 [0.70 - 0.71]	0.10 [0.09 - 0.11]	0.33 [0.32 - 0.34]	0.26 [0.25 - 0.28]	0.06 [0.03 - 0.08]
	**Stroma**	**Endothelium**	**Epithelium**	**Apoptosis**	**Micro F_1**	**Average F_1**
**Intra observer**	0.51 [0.37 - 0.64]	0.71 [0.59 - 0.84]	0.92 [0.77 - 1.07]	0.68 [0.57 - 0.79]	0.86 [0.86 - 0.86]	0.65 [0.61 - 0.70]
**Inter Observer**	0.34 [0.27 - 0.41]	0.45 [0.34 - 0.57]	0.91 [0.77 - 1.05]	0.60 [0.51 - 0.70]	0.80 [0.80 - 0.8]	0.54 [0.50 - 0.58]
**Hover-Net (PUMA)**	0.31 [0.29 - 0.32]	0.15 [0.14 - 0.17]	0.13 [0.12 - 0.14]	0.22 [0.21 - 0.24]	0.61 [0.61 - 0.61]	0.27 [0.27 - 0.28]
**Hover-NeXt (PUMA)**	0.21 [0.20 - 0.22]	0.24 [0.23 - 0.25]	0.04 [0.03 - 0.05]	0.04 [0.04 - 0.05]	0.61 [0.61 - 0.61]	0.27 [0.27 - 0.27]

Table 4. Nuclei count in intra and inter-observer ground truth dataset consisting out of 12 samples.

	**Tumor**	**Lymphocyte**	**Plasma cell**	**Histiocyte**	**Melanophage**
**Nuclei count**	3394	1242	2	353	14
	**Neutrophil**	**Stroma**	**Endothelium**	**Epithelium**	**Apoptosis**
**Nuclei count**	103	110	161	173	196

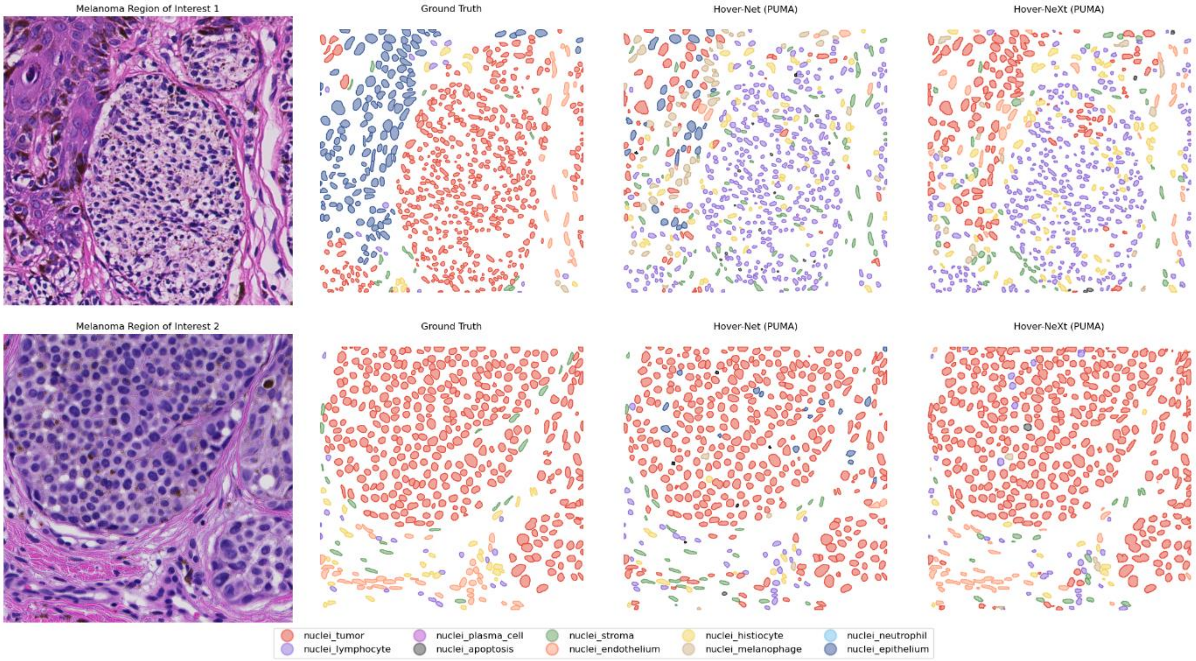

Figure 6. Visual results of segmentation of all nuclei classes. Results are shown for the Hover-Net and Hover-NeXt model trained on the PUMA dataset. In ROI 1 neither of the models is able to classify tumor nuclei and lymphocytes. In ROI 2 model performance is better with identification of tumor nuclei and endothelium nuclei.

Heuristic post processing: combining tissue and nuclei predictions

In the fourth experiment, we used the tissue mask as predicted by the nnU-Net model to improve nuclei segmentation. If a nucleus center was placed inside a tissue mask of the epidermis, the nuclei was classified as an epidermal nuclei. In addition, if a nucleus was present inside the blood vessel class, the nucleus was classified as endothelial nucleus. In Hover-Net and Hover-NeXt, the average F1 score increased from 0.27 and 0.27 to 0.31 and 0.32, respectively. This is mainly due to an increase in the F_1 score for epithelium.

Table 3. F_1 scores for heuristic post processing of the Hover-Net and Hover-NeXt output compared with their non-post processing output.

	**Tumor**	**Lymphocyte**	**Plasma cell**	**Histiocyte**	**Melanophage**	**Neutrophil**
**Hover-Net (PUMA)**	0.73 [0.72 - 0.73]	0.67 [0.66 - 0.67]	0.07 [0.06 - 0.09]	0.27 [0.26 - 0.27]	0.31 [0.29 - 0.33]	0.15 [0.13 - 0.18]
**Hover-Net (PUMA - post-processed)**	0.73 [0.73 - 0.73]	0.67 [0.66 - 0.67]	0.07 [0.05 - 0.08]	0.27 [0.26 - 0.27]	0.32 [0.30 - 0.33]	0.15 [0.12 - 0.17]
**Hover-NeXt (PUMA)**	0.70 [0.70 - 0.71]	0.71 [0.70 - 0.71]	0.10 [0.09 - 0.11]	0.33 [0.32 - 0.34]	0.26 [0.25 - 0.28]	0.06 [0.03 - 0.08]
**Hover-NeXt (PUMA – post processed)**	0.71 [0.71 - 0.71]	0.71 [0.70 - 0.71]	0.1 [0.09 - 0.11]	0.33 [0.32 - 0.34]	0.27 [0.25 - 0.28]	0.06 [0.04 - 0.08]
	**Stroma**	**Endothelium**	**Epithelium**	**Apoptosis**	**Micro F_1**	**Average F_1**
**Hover-Net (PUMA)**	0.31 [0.29 - 0.32]	0.15 [0.14 - 0.17]	0.13 [0.12 - 0.14]	0.22 [0.21 - 0.24]	0.61 [0.61 - 0.61]	0.27 [0.27 - 0.28]
**Hover-Net (PUMA - post-processed)**	0.31 [0.29 - 0.32]	0.2 [0.19 - 0.21]	0.44 [0.42 - 0.46]	0.22 [0.21 - 0.24]	0.62 [0.62 - 0.62]	0.31 [0.30 - 0.31]
**Hover-NeXt (PUMA)**	0.21 [0.20 - 0.22]	0.24 [0.23 - 0.25]	0.04 [0.03 - 0.05]	0.04 [0.04 - 0.05]	0.61 [0.61 - 0.61]	0.27 [0.27 - 0.27]
**Hover-NeXt (PUMA – post processed)**	0.21 [0.20 - 0.22]	0.25 [0.24 - 0.26]	0.52 [0.50 - 0.55]	0.05 [0.04 - 0.06]	0.62 [0.62 - 0.62]	0.32 [0.32 - 0.32]

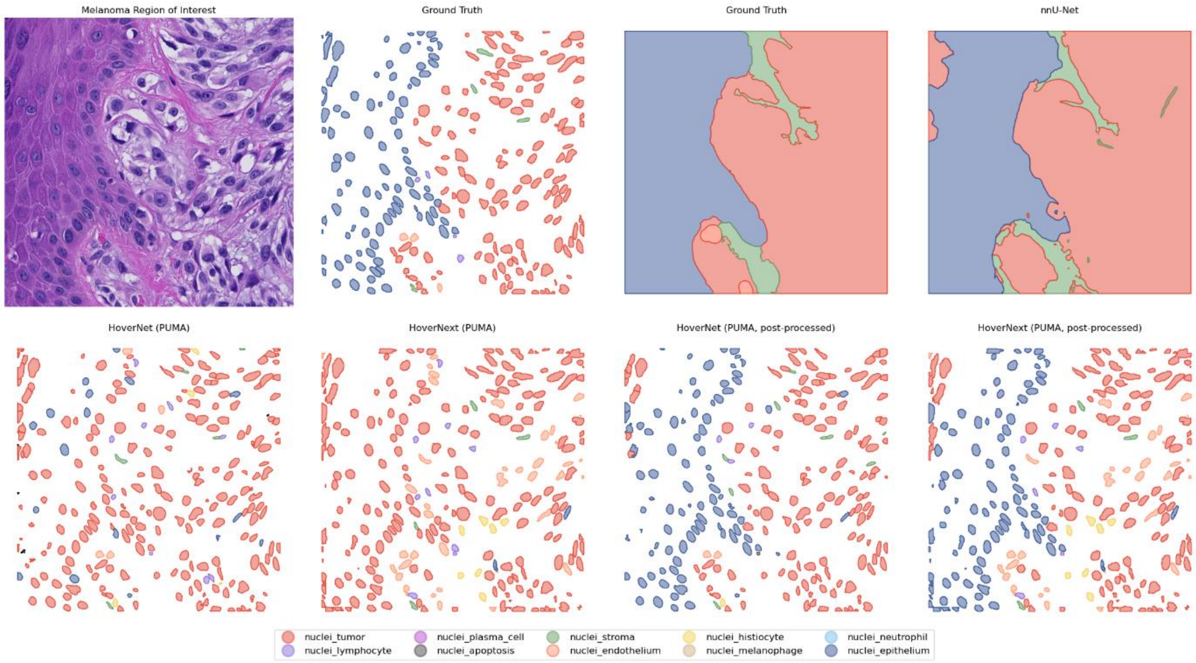

Figure 7. Visual results of heuristic post-processing. For clarification, the ground truth and original predictions of Hover-Net and Hover-NeXt are displayed.

Discussion

In the present study, we show the methodology used to create the PUMA dataset, which is the first melanoma specific nuclei and tissue dataset. In addition, we provide baseline benchmarks in which we show that the Hover-NeXt model trained with three classes (tumor, lymphocyte, and other) can detect lymphocytes almost on human interobserver level performance. Finally, we show an example of the integration of tissue and nuclei annotations leading to improved nuclei segmentation. We believe that further improvement of deep learning models through this integration is possible, and therefore, the dataset will be used to organize the PUMA challenge.

Earlier studies regarding nuclei segmentation have been performed with multiple tissue datasets [17,27] or other tissue specific datasets in, for example, breast cancer [34] and colon cancer [35]. However, thus far, no melanoma specific dataset is available. Since melanocytes and melanoma cells can mimic other cell types, such as lymphocytes and stroma cells, models created with these dataset are not fully applicable for use in melanoma histopathology [13,18]. Models created with this dataset, will be usable for creation of prognostic and/or predictive biomarkers in melanoma histopathology.

The PUMA dataset combines tissue and nuclei segmentation. Earlier studies, such as the OCELOT challenge [36] and the MuTILs model [37] showed that incorporating tissue masks in nuclei prediction increased the capability of models which detect either tumor nuclei or TILs. With this dataset, the goal is to see whether implementation of tissue masks can improve multi class nuclei segmentation for classes such epidermis, endothelium, tumor, and immune cell subsets. In this paper, we demonstrated this by means of a simple post-processing technique. However, other solutions might be passing information from a tissue segmentation model directly to a nuclei segmentation model or making use of multiple sequential models [36,38]. To make use of the creativity and knowledge of the deep learning community, we initiated the PUMA challenge to assess how precise segmentation of melanoma samples can become. Participants in the PUMA challenge can choose to participate in two

tracks. For track 1 the output needs to be nuclei segmentation for three classes (tumor, lymphocyte and other) and tissue segmentation for all classes whereas in track 2 participants need to segment all nuclei classes and tissue segmentation for all classes.

With the dataset, we also aimed to assess the performance of existing models usable in skin and/or melanoma histopathology. Due to unavailability, we were unable to assess the LUNIT scope IO model. However, we were able to evaluate the NN192 algorithm, which is used in multiple studies in which lymphocytes in melanoma histopathology were associated with a decreased chance of recurrence [39] and an increased chance of responding to immune checkpoint inhibition therapy [7]. Surprisingly, the NN192 model showed the lowest capability to detect lymphocytes. This might be due to variations in staining and scanners used in the studies performed earlier and the inability of the techniques used to correct for this. The Hover-Net and Hover-NeXt algorithm architectures can compensate for this by extensive data augmentation in the training (Hover-Net, Hover-NeXt) and inference stages (Hover-NeXt), accounting for better results with the out of the box model trained on the PanNuke dataset. However, both models trained on the PanNuke dataset still showed diminished performance in more difficult to segment melanoma samples when compared to the Hover-NeXt model trained on the PUMA dataset itself.

A strength of this study is the annotation process by a medical expert and an expert dermatopathologist using context ROI to more accurately segment and classify nuclei. In addition, we validated the annotations with a second independent pathologist (GB). From this, it became clear that there is a low interobserver agreement in the less common classes, such as histiocytes, melanophages, and stroma. Melanophages are a subtype of histocytes that have phagocytosed melanin, resulting in a more pigmented appearance. However, there is a continuum between the cells, which could explain the difference in classification. Stroma cells, in addition, are present just like histiocytes in the tissue

around the tumor, and it is not always possible to distinguish them from histiocytes on H&E stained slides.

A second strong point of the study is the manual selection of ROIs. This allowed us to focus on immune cell subsets and less common nuclei and tissue classes. In addition, we tried to include regions with artifacts or much pigmentation. We believe this makes the dataset and the models more applicable to whole slide images, which by nature have artifacts, unsharp regions, and, especially in the case of melanoma, pigmentated more difficult to segment regions.

A downside of the dataset and the study is that all samples are, due to privacy regulations, from one scanner and a single hospital. However, 76 out of 310 cases are consultation cases from referral hospitals already leading to substantial staining variation. In addition, we believe that through stain normalization and data augmentation, models can become less sensible to domain-adversarial effects. This makes models trained on this dataset applicable to whole slide images from other hospitals and scanner types.

Our study did not demonstrate high performance in segmentation of all nuclei classes. This is partly due to the used models losing discriminative ability by introducing more classes. In addition, some classes are less common in the dataset such as apoptosis or histiocytes. Furthermore, there is overlap between classes such as melanophages and histiocytes. We believe that further improvement of models is possible and hope to that through leveraging the knowledge of the deep learning community in the PUMA challenge this will become more clear.

In conclusion, we created a novel dataset with tissue and nuclei segmentations in advanced cutaneous melanoma. With this dataset we showed that deep learning based lymphocyte segmentation can achieve performance levels close to those of human interobserver agreement. In addition, we

demonstrated that it is possible to more accurately segment nuclei classes by incorporating tissue predictions. However, we believe that further improvement is possible by further integration of tissue and nuclei segmentation. Future work in the PUMA challenge will demonstrate to what extent segmentation of different nuclei can be improved.

Potential implications

The PUMA dataset and the deep learning models created with this dataset can be used for biomarker generation in the treatment of melanoma. Thus far, it is known that lymphocytes have predictive value for melanoma recurrence and immune checkpoint inhibition treatment response [6,7,40]. However, this is done either by manual scoring or scoring by automated models, which are not generalizable to new unseen tissue and scanners. With this public dataset and the generalizable deep learning models developed from it, the next step will be to translate these models into clinical application. This will result in more personalized treatment plans, such as de-escalation of immune checkpoint inhibition therapy in patients with advanced melanoma or a less intensive follow-up regimen in patients with primary melanoma. Next to the assessment of TILs as an explainable biomarker, this study will also enable future biomarker discovery studies through the assessment of other nuclei and tissue types.

In addition, this study adds a new step towards further improvement of nuclei segmentation deep learning models in H&E stained histological slides by combining tissue segmentation models into multiclass nuclei segmentation models. This process is closer to how a pathologist evaluates histological slides and already resulted in improved nuclei segmentation benchmark scores.

Methods

Dataset generation

For the dataset, digitized melanoma whole slide H&E stained images generated through regular diagnostic procedures were used from the archive of the UMC Utrecht. All slides were scanned with a Nanozoomer XR C12000–21/− 22 (Hamamatsu Photonics, Hamamatsu, Shizuoka, Japan) at 40× magnification with a resolution of 0.23 µm per pixel. Out of 310 cases in the dataset, 76 are consultation cases from referral hospitals or general practitioners leading to variation in used staining protocols. From each slide, a 40× magnified ROI of 1024×1024 pixels was selected for annotation. In addition, a context ROI of 5120×5120 pixels was sampled to provide information about the broader context for the annotation process. Selection was done by a trained medical expert (M.S.) and subsequently verified by an expert dermatopathologist (W.B.). Manual ROI selection ensured diverse tissue and nuclei types and the inclusion of more difficult to segment areas due to blurring, pigmentation, and scanning artifacts.

Nuclei segmentations were generated with Hover-Net pretrained on the PanNuke dataset [16,17]. Manual annotation adjustment was performed by author M.S. using Qupath with the following nuclei categories: tumor, stroma, vascular endothelium, histiocyte, melanophage, lymphocyte, plasma cell, neutrophil, apoptotic and epithelium [29]. Annotation categories were based on earlier datasets. In addition, we chose categories based on possible predictive value. All annotations were checked and corrected where needed by a dermatopathologist (W.B.). Intra- and inter-observer agreement (by pathologist G.B.) were determined on 12 randomly selected ROIs.

Tissue segmentations were created manually with QuPath by author M.S. using the following categories: tumor, stroma, epidermis, necrosis, blood vessel, and background. Annotation categories are based on the approach used by Hwang et al., who segmented TILs in tumor and stroma areas, and the guidelines from the International Immuno-Oncology Biomarker Working Group [15,41].

Annotations are checked and corrected when needed by a dermatopathologist (W.B.). Intra- and inter-observer agreement (by pathologist G.B.) were determined on 12 randomly selected ROIs to ensure the inclusion of all tissue types.

Benchmark models

Nuclei segmentation

To establish baseline nuclei segmentation benchmarks, we performed nuclei segmentation for two sets of experiments. The first experiment compares models that output three nuclei categories: tumor, lymphocyte, and other. The second experiment is an analysis of the segmentation of all individual nuclei categories. Model training was performed with 5-fold cross validation on the public training dataset without adjusting training parameters or data augmentation of the algorithm used. Inference was performed on the 94 ROIs of the final hidden test set of the PUMA challenge. The remaining 10 ROIs will be used in the PUMA challenge for sanity checking of submitted models.

For the first experiment, the following models were compared: NN192, Hover-Net trained on the PanNuke dataset, Hover-Net trained on the PUMA dataset, Hover-Next trained on the PanNuke dataset, Hover-Next trained on the PUMA dataset [16,17,40,42]. The NN192 model outputs four categories: tumor, lymphocytes, stroma, and other. From this output, the stroma and other categories were merged into one other category. The models trained on the PanNuke dataset classify nuclei into 5 categories: neoplastic, non-neoplastic epithelial, inflammatory, connective, and apoptotic. For the benchmark comparison, neoplastic and inflammatory nuclei were used; non-neoplastic epithelial, connective, and apoptotic were merged into the other category. For training on the PUMA dataset, the plasma cell and lymphocyte categories were merged into a single lymphocyte category, and the remaining classes were combined into the other category. For the second experiment, Hover-Net and Hover-NeXt were trained on all samples and compared.

For the calculation of the F1 score, the center distance between the predicted nuclei and the ground truth nuclei was used. For each ground truth nucleus, predictions within a 15 pixel (3.3 µm) were identified. This radius is smaller than the average size of lymphocytes, which form the smallest nuclei in the dataset. Matching was performed based on the highest predictive score (if available) or the shortest distance. After matching, the ground truth was censored until all ground truth nuclei were either matched or classified as a false negative. Using the identified true positives, false positives, and false negatives, precision (all correct predictions divided by all predictions) and recall (all correct predictions divided by all ground truth nuclei) were calculated. The class F1 score was computed as the harmonic mean of precision and recall, ranging from 0 to 1. Finally to compare models, micro F_1 (aggregation of TP, FP, and FN over all classes, followed by F1 score calculation) and Average F_1 (the average of class F_1 scores) were calculated [43]. Results are shown with a 95% confidence interval which is calculated through bootstrapping the samples.

Tissue Segmentation

For tissue segmentation, nnU-Net and Mask2Former were used to establish baseline benchmarks [31,44]. For training of nnU-Net the same fivefold cross validation was used to create an ensemble model. Mask2Former was pretrained on the COCO instance segmentation task using a Swin Transformer backbone. Images were resized to 512 × 512 pixels before loading into the model. For our experiment we replaced the backbone with the UNI pathology foundation model, which is better able at feature extraction from H&E stained histopathology, after which we finetuned the model on the whole training dataset [32,33,45]. Both models were used for inference on the final hidden test set from the PUMA challenge.

Both models and intra- and interobserver agreement were evaluated using the DICE score. The DICE score is a harmonic mean between 0 and 1, in which 1 is a perfect segmentation prediction, and 0 is no correct prediction. This can result in inflated high average DICE scores if a tissue class is only

present in a few samples, as the DICE score is 1 in the case of a correct absent prediction. To accommodate this, we calculated not only the average DICE score over all samples but also the micro average DICE. This is the DICE score for all predictions concatenated along one axis, resulting in a prediction mask of 1024 × 96.256 pixels. Results are shown with a 95% confidence interval which is generated through bootstrapping the sample results.

Post-processing

For post-processing nuclei, centers were calculated using the Point function from the python Shapely library. From these points, the spatial location inside a tissue mask prediction was determined by using the GeoPandas Python Package. Based on this location the classification label of the nuclei was adjusted.

Availability of source code and requirements

Project name: PUMA

Project home page: https://puma.grand-challenge.org/

Operating system: Platform independent

Programming language: Python 3.10

Other requirements: Hover-Net (available at: https://github.com/vqdang/hover_net), Hover-NeXt (https://github.com/digitalpathologybern/hover_next_train and https://github.com/digitalpathologybern/hover_next_inference) .

nnU-Net (available at: https://github.com/MIC-DKFZ/nnUNet) and Mask2Former (available at https://huggingface.co/docs/transformers/en/model_doc/mask2former).

QuPath 0.5.0 (available at https://qupath.github.io/)

The NN192 classification algorithm (available at https://github.com/acsbal/Automated-TIL-scoring-QuPath-Classifier-for-Melanoma) and QuPath 0.1.2 (https://github.com/qupath/qupath/releases/tag/v0.1.2).

The code and weights for the PUMA challenge inference baseline solutions and metric calculation can be found here: (https://zenodo.org/records/13897135 , https://github.com/tueimage/PUMA-challenge-eval-track1, https://github.com/tueimage/PUMA-challenge-eval-track2, https://github.com/tueimage/PUMA-challenge-baseline-track1, https://github.com/tueimage/PUMA-challenge-baseline-track2)

Qupath scripts to import .geoJSONs for viewing and exporting annotations as masks can be found here: https://zenodo.org/records/13897135.

No changes were made to the training code for the used algorithms.

License: The PUMA codebase is licensed with a CC0 1.0 license (dataset) and the MIT license.

Restrictions to use by non-academics: Both the CC0 1.0 license (dataset) and the MIT license (codebase) allow for non-commercial use. License terms can be reviewed for details.

Data Availability

The PUMA training dataset is available on Zenodo at: https://zenodo.org/records/10940194. Joining the PUMA challenge is possible through the grand challenge platform at https://puma.grand-challenge.org/. The preliminary and final test set will stay hidden until October 10th 2029 due the PUMA challenge still being open for submissions until then. Readers can request earlier access to the hidden dataset for educational or collaborative purposes by contacting the corresponding author via email.

List of abbreviations

TILs: Tumor infiltrating lymphocytes

H&E: Hematoxylin and Eosin

ROI: Region of interest

Declaration

The Biobank Research Ethics Committee (TCBio) UMC Utrecht confirms that it has reviewed the release file in accordance with the UMC Utrecht Biobank Regulations and all other applicable regulations and laws. Based on the requirements as defined in these regulations and laws, the TCBio UMC Utrecht hereby issues an approval of the aforementioned dataset (reference number TCBio 23-270/U-B).

Competing interests

The authors declare the following financial interests/personal relationships which may be considered as potential competing interests:

Karijn P.M. Suijkerbuijk reports a consulting/advisory relationship with Abbvie and Sairopa. She received honoraria from Bristol Myers Squibb and research funding from TigaTx, Bristol Myers Squibb, Philips, Genmab and Pierre Fabre. All paid to institution

The remaining authors of this manuscript have no conflicts of interest to disclose.

Funding

This research was funded by an unrestricted grant of Stichting Hanarth Fonds, The Netherlands.

Authors' contributions

Conceptualization: Schuiveling, Blokx, Suijkerbuijk, Veta

Methodology: Schuiveling, Blokx, Veta, Liu, Breimer

Software: Schuiveling, Eek, Liu

Investigation: Schuiveling, Liu

Resources: Blokx, Suijkerbuijk, Veta

Data Curation: Schuiveling, Blokx, Breimer

Writing, Original Draft: Schuiveling, Liu

Writing, Review & Editing: Blokx, Suijkerbuijk, Veta, Eek, Breimer

Supervision: Blokx, Suijkerbuijk, Veta

Project administration: Blokx, Suijkerbuijk, Veta

Funding acquisition: Suijkerbuijk, Blokx, Veta

References

1. Rahib L, Wehner MR, Matrisian LM, Nead KT. Estimated Projection of US Cancer Incidence and Death to 2040. *JAMA Netw Open*. 2021; doi: 10.1001/jamanetworkopen.2021.4708.

2. Postow MA, Sidlow R, Hellmann MD. Immune-Related Adverse Events Associated with Immune Checkpoint Blockade. *N Engl J Med*. 2018; doi: 10.1056/NEJMra1703481.

3. van Not OJ, Blokx WAM, van den Eertwegh AJM, de Meza MM, Haanen JB, Blank CU, et al.. BRAF and NRAS Mutation Status and Response to Checkpoint Inhibition in Advanced Melanoma. *JCO Precis Oncol*. 2022; doi: 10.1200/PO.22.00018.

4. Franken MG, Leeneman B, Aarts MJB, van Akkooi ACJ, van den Berkmortel FWPJ, Boers-Sonderen MJ, et al.. Trends in survival and costs in metastatic melanoma in the era of novel targeted and immunotherapeutic drugs. *ESMO Open*. 2021; doi: 10.1016/j.esmoop.2021.100320.

5. Suijkerbuijk KPM, van Eijs MJM, van Wijk F, Eggermont AMM. Clinical and translational attributes of immune-related adverse events. *Nat Cancer*. 2024; doi: 10.1038/s43018-024-00730-3.

6. van Duin IAJ, Schuiveling M, ter Maat LS, van Amsterdam WAC, van den Berkmortel F, Boers-Sonderen M, et al.. Baseline tumor-infiltrating lymphocyte patterns and response to immune checkpoint inhibition in metastatic cutaneous melanoma. *European Journal of Cancer*. 2024; doi: 10.1016/j.ejca.2024.114190.

7. Chatziioannou E, Roßner J, Aung TN, Rimm DL, Niessner H, Keim U, et al.. Deep learning-based scoring of tumour-infiltrating lymphocytes is prognostic in primary melanoma and predictive to PD-1 checkpoint inhibition in melanoma metastases. *eBioMedicine*. Elsevier; 2023; doi: 10.1016/j.ebiom.2023.104644.

8. Fu Q, Chen N, Ge C, Li R, Li Z, Zeng B, et al.. Prognostic value of tumor-infiltrating lymphocytes in melanoma: a systematic review and meta-analysis. *Oncoimmunology*. 2019; doi: 10.1080/2162402X.2019.1593806.

9. Clark WH, Elder DE, Guerry D, Braitman LE, Trock BJ, Schultz D, et al.. Model predicting survival in stage I melanoma based on tumor progression. *J Natl Cancer Inst*. 1989; doi: 10.1093/jnci/81.24.1893.

10. Hendry S, Salgado R, Gevaert T, Russell PA, John T, Thapa B, et al.. Assessing Tumor-Infiltrating Lymphocytes in Solid Tumors: A Practical Review for Pathologists and Proposal for a Standardized Method from the International Immuno-Oncology Biomarkers Working Group: Part 2: TILs in Melanoma, Gastrointestinal Tract Carcinomas, Non-Small Cell Lung Carcinoma and Mesothelioma, Endometrial and Ovarian Carcinomas, Squamous Cell Carcinoma of the Head and Neck, Genitourinary Carcinomas, and Primary Brain Tumors. *Adv Anat Pathol*. 2017; doi: 10.1097/PAP.0000000000000161.

11. Busam KJ, Antonescu CR, Marghoob AA, Nehal KS, Sachs DL, Shia J, et al.. Histologic classification of tumor-infiltrating lymphocytes in primary cutaneous malignant melanoma. A study of interobserver agreement. *Am J Clin Pathol*. 2001; doi: 10.1309/G6EK-Y6EH-0LGY-6D6P.

12. Swisher SK, Wu Y, Castaneda CA, Lyons GR, Yang F, Tapia C, et al.. Interobserver Agreement Between Pathologists Assessing Tumor-Infiltrating Lymphocytes (TILs) in Breast Cancer Using

Methodology Proposed by the International TILs Working Group. *Ann Surg Oncol*. 2016; doi: 10.1245/s10434-016-5173-8.

13. Schuiveling M, Blokx WAM, Breimer GE, Suijkerbuijk KPM, Eek D, Veta M. A Novel Dataset for Nuclei Segmentation in Melanoma Histopathology. 2024;

14. : Inflamed immune phenotype predicts favorable clinical outcomes of immune checkpoint inhibitor therapy across multiple cancer types | Journal for ImmunoTherapy of Cancer. https://jitc.bmj.com/content/12/2/e008339 Accessed 2024 Apr 18.

15. Hwang S, Ryu HJ, Kim Y, Cho SI, Oum C, Valero Puche A, et al.. Immune phenotype profiling based on anatomic origin of melanoma and impact on clinical outcomes of immune checkpoint inhibitor treatment. *JCO*. 2024; doi: 10.1200/JCO.2024.42.16_suppl.9569.

16. Graham S, Vu QD, Raza SEA, Azam A, Tsang YW, Kwak JT, et al.. Hover-Net: Simultaneous segmentation and classification of nuclei in multi-tissue histology images. *Medical Image Analysis*. 2019; doi: 10.1016/j.media.2019.101563.

17. Gamper J, Koohbanani NA, Benes K, Graham S, Jahanifar M, Khurram SA, et al.. PanNuke Dataset Extension, Insights and Baselines. arXiv;

18. Ronen S, Czaja RC, Ronen N, Pantazis CG, Iczkowski KA. Small Cell Variant of Metastatic Melanoma: A Mimicker of Lymphoblastic Leukemia/Lymphoma. *Dermatopathology*. 2019; doi: 10.1159/000503703.

19. Magro CM, Crowson AN, Mihm MC. Unusual variants of malignant melanoma. *Mod Pathol*. 2006; doi: 10.1038/modpathol.3800516.

20. Mahbod A, Polak C, Feldmann K, Khan R, Gelles K, Dorffner G, et al.. NuInsSeg: A fully annotated dataset for nuclei instance segmentation in H&E-stained histological images. *Sci Data*. Nature Publishing Group; 2024; doi: 10.1038/s41597-024-03117-2.

21. Mahbod A, Schaefer G, Bancher B, Löw C, Dorffner G, Ecker R, et al.. CryoNuSeg: A dataset for nuclei instance segmentation of cryosectioned H&E-stained histological images. *Computers in Biology and Medicine*. 2021; doi: 10.1016/j.compbiomed.2021.104349.

22. Komura D, Onoyama T, Shinbo K, Odaka H, Hayakawa M, Ochi M, et al.. Restaining-based annotation for cancer histology segmentation to overcome annotation-related limitations among pathologists. *Patterns*. 2023; doi: 10.1016/j.patter.2023.100688.

23. Park S, Ock C-Y, Kim H, Pereira S, Park S, Ma M, et al.. Artificial Intelligence–Powered Spatial Analysis of Tumor-Infiltrating Lymphocytes as Complementary Biomarker for Immune Checkpoint Inhibition in Non–Small-Cell Lung Cancer. *JCO*. Wolters Kluwer; 2022; doi: 10.1200/JCO.21.02010.

24. Choi S, Cho SI, Jung W, Lee T, Choi SJ, Song S, et al.. Deep learning model improves tumor-infiltrating lymphocyte evaluation and therapeutic response prediction in breast cancer. *npj Breast Cancer*. Nature Publishing Group; 2023; doi: 10.1038/s41523-023-00577-4.

25. Helmink BA, Reddy SM, Gao J, Zhang S, Basar R, Thakur R, et al.. B cells and tertiary lymphoid structures promote immunotherapy response. *Nature*. Nature Publishing Group; 2020; doi: 10.1038/s41586-019-1922-8.

26. Landsberg J, Tüting T, Barnhill RL, Lugassy C. The Role of Neutrophilic Inflammation, Angiotropism, and Pericytic Mimicry in Melanoma Progression and Metastasis. *Journal of Investigative Dermatology*. 2016; doi: 10.1016/j.jid.2015.11.013.

27. : MoNuSAC2020: A Multi-Organ Nuclei Segmentation and Classification Challenge | IEEE Journals & Magazine | IEEE Xplore. https://ieeexplore.ieee.org/abstract/document/9446924?casa_token=RVD5ZjFPUJwAAAAA:WqR-dWkLchFwwdtKJGo7GQwwylvOOL6cM796VDb6CDnprbLCYtcm-oMp-0W3MfHiub-Ot0Ab-g Accessed 2024 Jan 8.

28. Butler H, Daly M, Doyle A, Gillies S, Schaub T, Hagen S. The GeoJSON Format. Internet Engineering Task Force; 2016 Aug. Report No.: RFC 7946.

29. : QuPath: Open source software for digital pathology image analysis | Scientific Reports. https://www.nature.com/articles/s41598-017-17204-5 Accessed 2023 Sep 1.

30. Schuiveling M. Melanoma Histopathology Dataset with Tissue and Nuclei Annotations. Zenodo; 2024; doi: 10.5281/zenodo.10940193.

31. Isensee F, Jaeger PF, Kohl SAA, Petersen J, Maier-Hein KH. nnU-Net: a self-configuring method for deep learning-based biomedical image segmentation. *Nat Methods*. Nature Publishing Group; 2021; doi: 10.1038/s41592-020-01008-z.

32. : Towards a general-purpose foundation model for computational pathology | Nature Medicine. https://www.nature.com/articles/s41591-024-02857-3 Accessed 2024 Sep 23.

33. Cheng B, Misra I, Schwing AG, Kirillov A, Girdhar R. Masked-attention Mask Transformer for Universal Image Segmentation. arXiv;

34. : NuCLS: A scalable crowdsourcing approach and dataset for nucleus classification and segmentation in breast cancer | GigaScience | Oxford Academic. https://academic.oup.com/gigascience/article/doi/10.1093/gigascience/giac037/6586817 Accessed 2023 Sep 4.

35. Graham S, Jahanifar M, Azam A, Nimir M, Tsang Y-W, Dodd K, et al.. Lizard: A Large-Scale Dataset for Colonic Nuclear Instance Segmentation and Classification. *2021 IEEE/CVF International Conference on Computer Vision Workshops (ICCVW)*. Montreal, BC, Canada: IEEE;

36. Ryu J, Puche AV, Shin J, Park S, Brattoli B, Lee J, et al.. OCELOT: Overlapped Cell on Tissue Dataset for Histopathology. p. 23902–12.

37. Liu S, Amgad M, More D, Rathore MA, Salgado R, Cooper LAD. A panoptic segmentation dataset and deep-learning approach for explainable scoring of tumor-infiltrating lymphocytes. *npj Breast Cancer*. Nature Publishing Group; 2024; doi: 10.1038/s41523-024-00663-1.

38. Amgad M, Salgado R, Cooper LAD. MuTILs: A multiresolution deep-learning model for interpretable scoring of Tumor-Infiltrating Lymphocytes in breast carcinomas using clinical guidelines. medRxiv;

39. Aung TN, Shafi S, Wilmott JS, Nourmohammadi S, Vathiotis I, Gavrielatou N, et al.. Objective assessment of tumor infiltrating lymphocytes as a prognostic marker in melanoma using machine learning algorithms. *eBioMedicine*. Elsevier; 2022; doi: 10.1016/j.ebiom.2022.104143.

40. Acs B, Ahmed FS, Gupta S, Wong PF, Gartrell RD, Sarin Pradhan J, et al.. An open source automated tumor infiltrating lymphocyte algorithm for prognosis in melanoma. *Nat Commun*. 2019; doi: 10.1038/s41467-019-13043-2.

41. Thagaard J, Broeckx G, Page DB, Jahangir CA, Verbandt S, Kos Z, et al.. Pitfalls in machine learning-based assessment of tumor-infiltrating lymphocytes in breast cancer: A report of the International Immuno-Oncology Biomarker Working Group on Breast Cancer. *J Pathol*. 2023; doi: 10.1002/path.6155.

42. Baumann E, Dislich B, Rumberger JL, Nagtegaal ID, Martınez MR, Zlobec I. HoVer-NeXt: A Fast Nuclei Segmentation and Classification Pipeline for Next Generation Histopathology. *Medical Imaging with Deep Learning*. 2024;

43. Maier-Hein L, Reinke A, Godau P, Tizabi MD, Buettner F, Christodoulou E, et al.. Metrics reloaded: recommendations for image analysis validation. *Nat Methods*. Nature Publishing Group; 2024; doi: 10.1038/s41592-023-02151-z.

44. Cao H, Wang Y, Chen J, Jiang D, Zhang X, Tian Q, et al.. Swin-Unet: Unet-Like Pure Transformer for Medical Image Segmentation. In: Karlinsky L, Michaeli T, Nishino K, editors. *Computer Vision – ECCV 2022 Workshops*. Cham: Springer Nature Switzerland;

45. Lin T-Y, Maire M, Belongie S, Bourdev L, Girshick R, Hays J, et al.: Microsoft COCO: Common Objects in Context. arXiv.org. https://arxiv.org/abs/1405.0312v3 (2014). Accessed 2024 Sep 6.

Figure

Click here to access/download;Figure;fig1.pdf

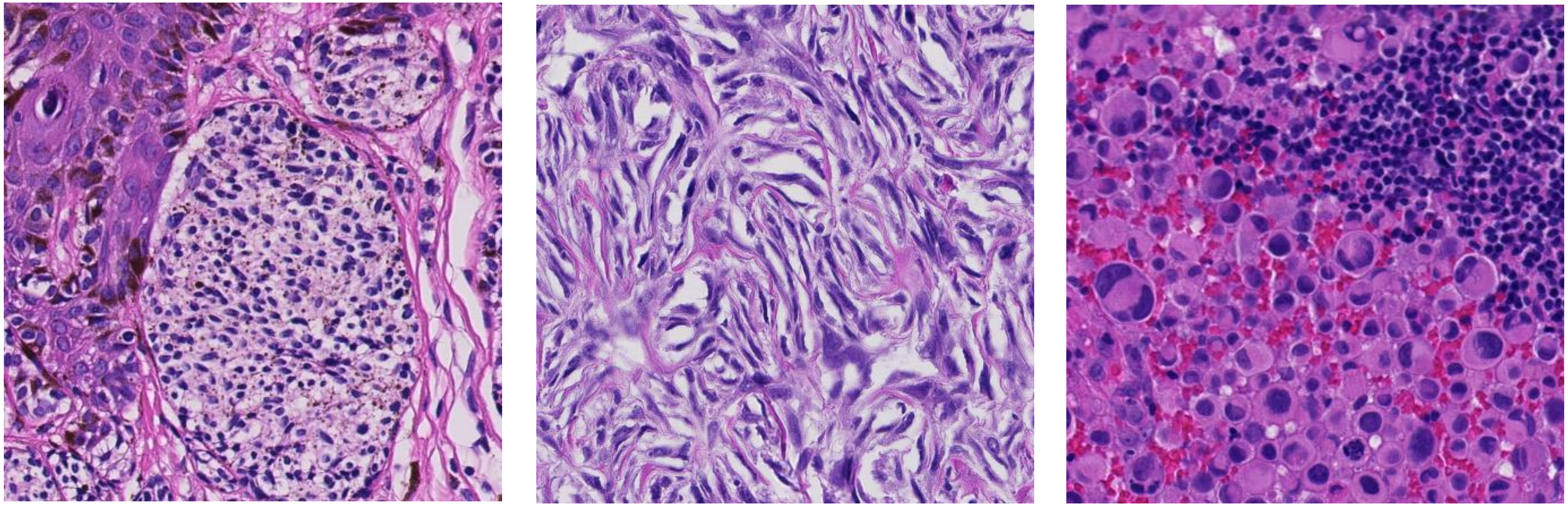

Figure 2

Click here to access/download;Figure;fig2.pdf

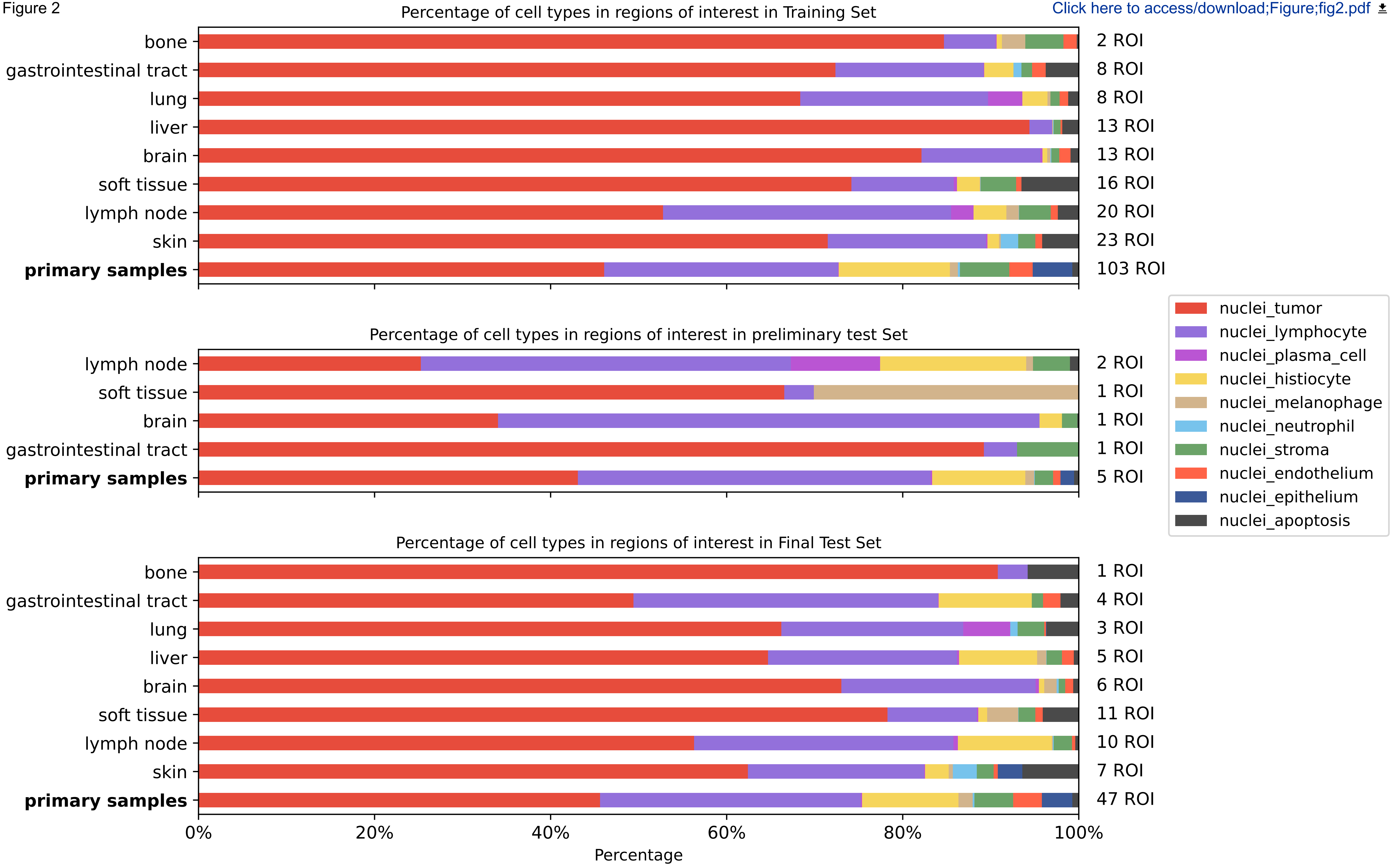

Figure 3

Click here to access/download;Figure;fig3.pdf

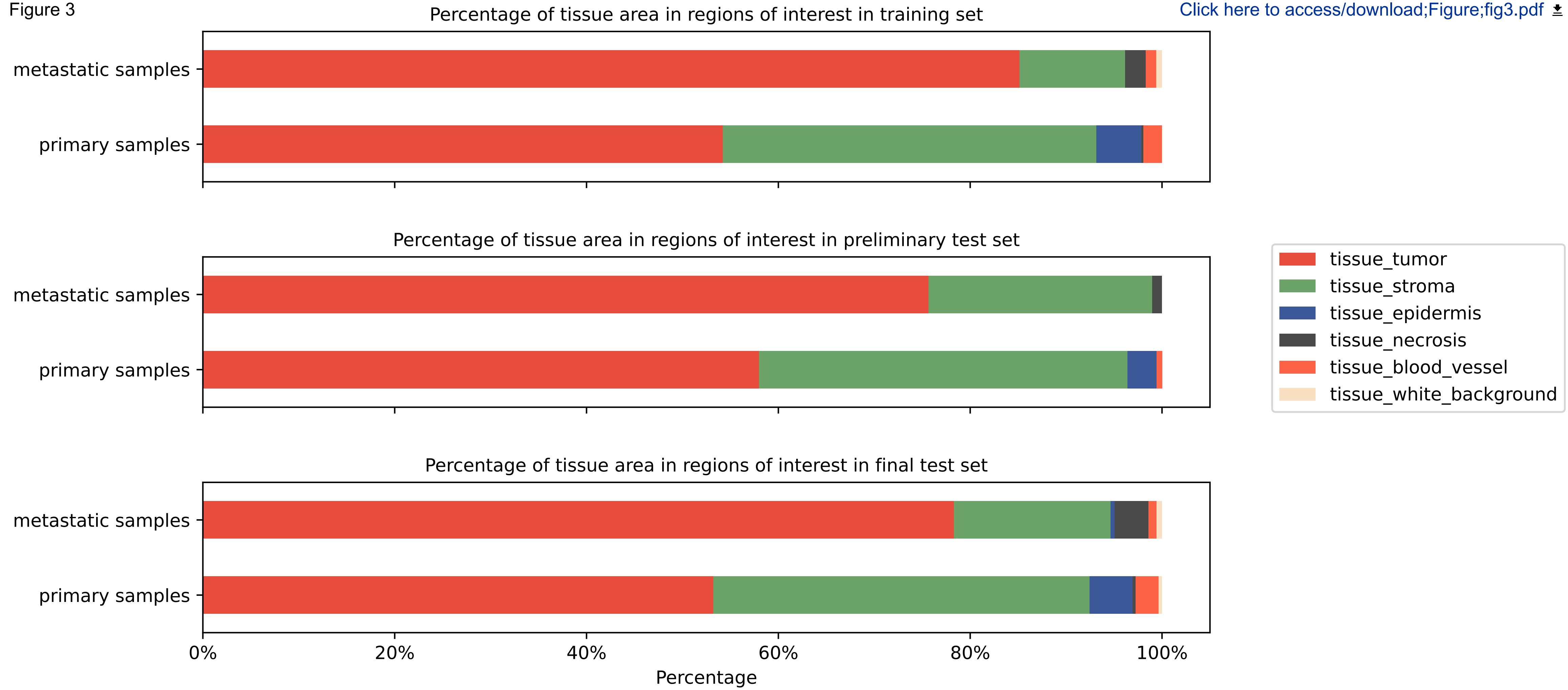

Figure 4

Click here to access/download;Figure;fig4.pdf

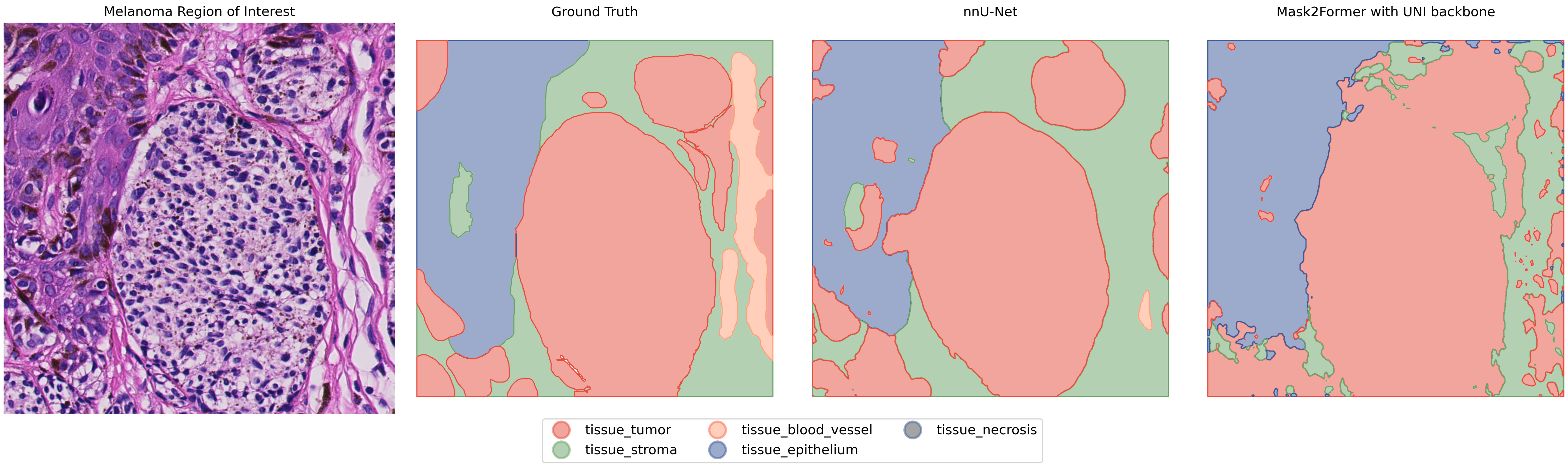

Figure 5

Click here to access/download;Figure;fig5.pdf

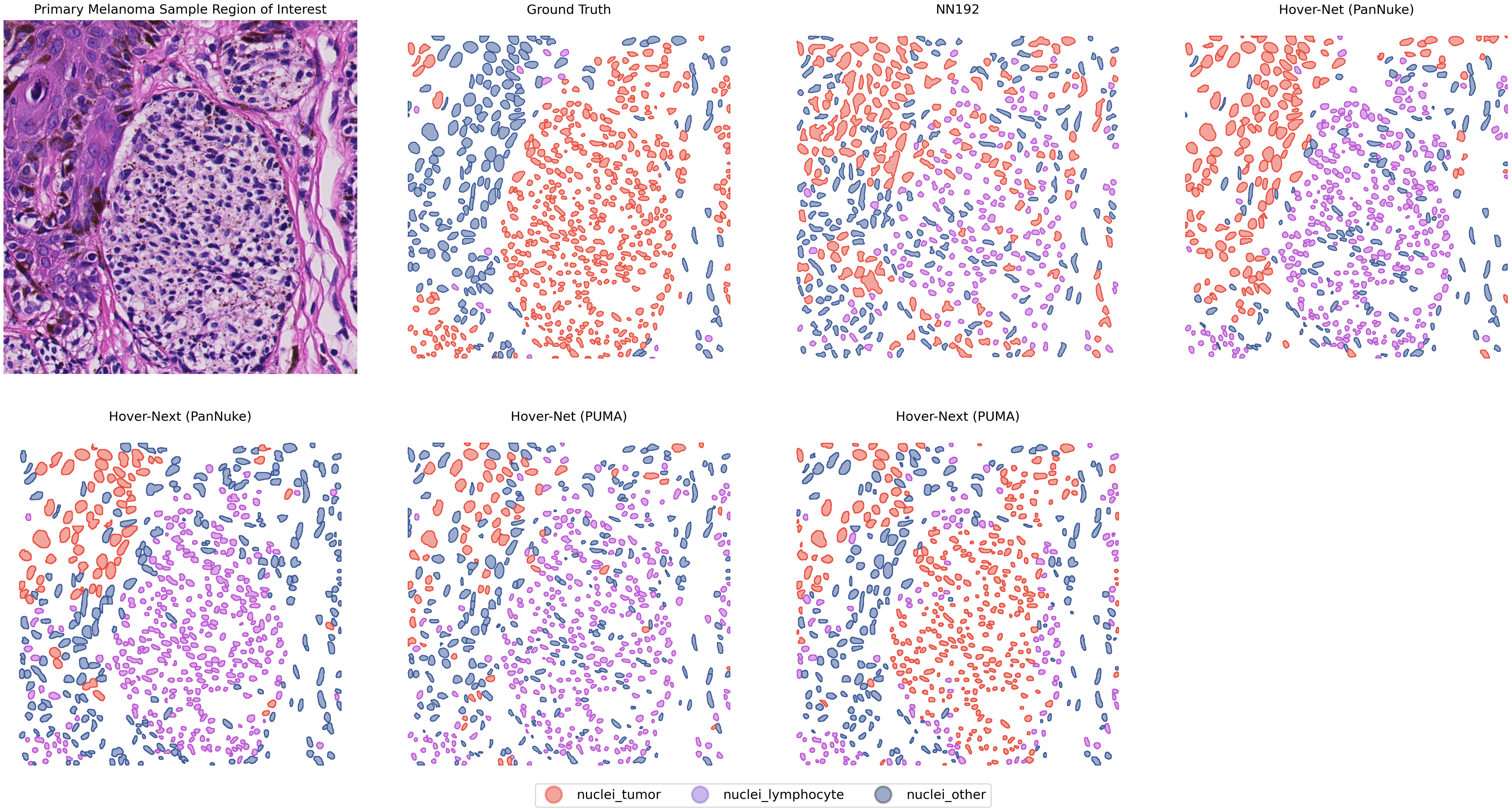

Figure 6

Click here to access/download;Figure;fig6.pdf

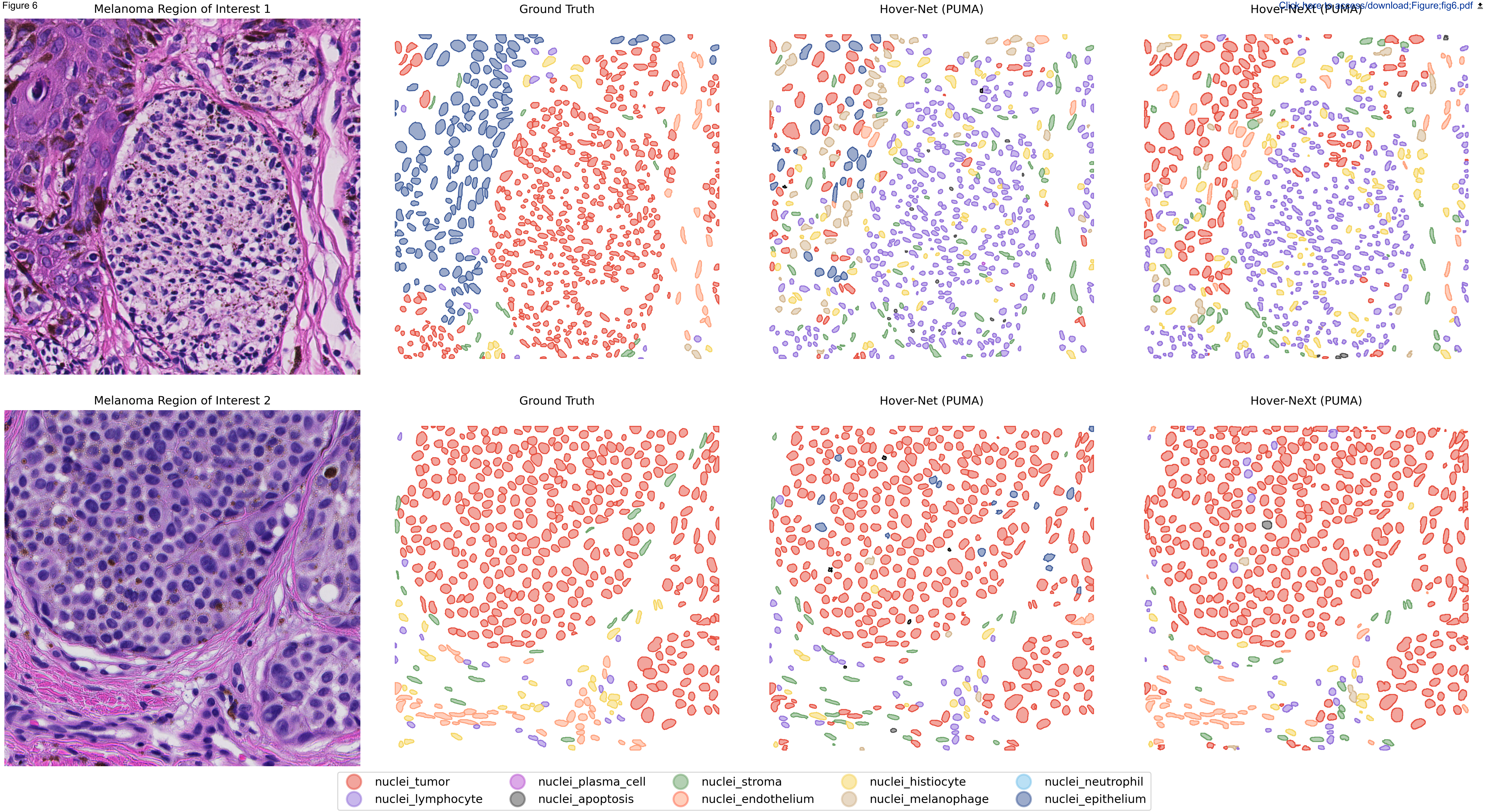

Figure 7

Click here to access/download;Figure;fig7.pdf

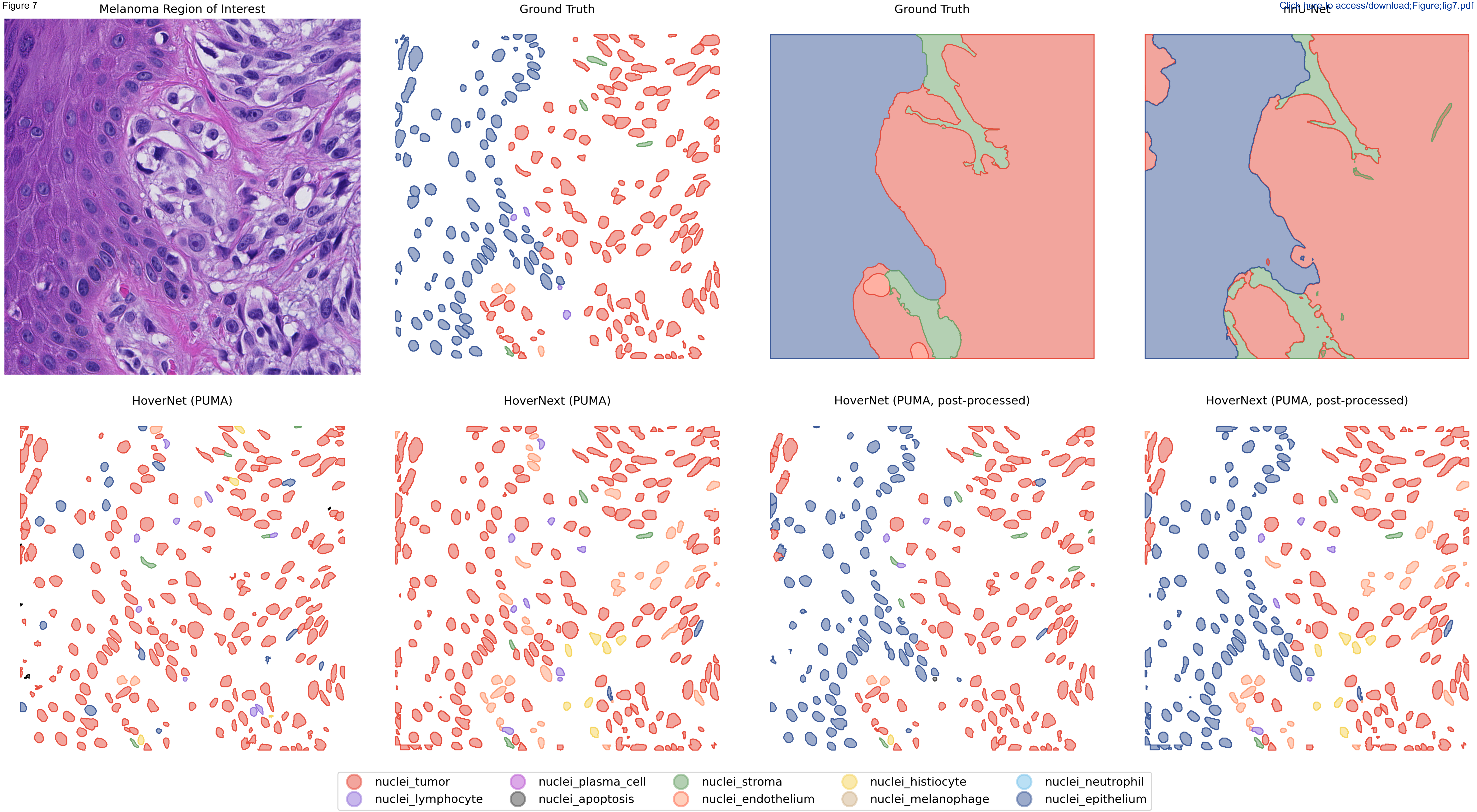

Supplement: giaf011_GIGA-D-24-00434_Revision_1 [file giaf011_giga-d-24-00434_revision_1.pdf]
