# Supplementary material for: A novel dataset for nuclei and tissue segmentation in melanoma with baseline nuclei segmentation and tissue segmentation benchmarks
Source: Gigascience. 2025 Feb 19;14:giaf011. doi: 10.1093/gigascience/giaf011 (PMC11837757; doi:10.1093/gigascience/giaf011)
Supplement: giaf011_Response_to_Reviewer_Comments_Original_Submission [file giaf011_response_to_reviewer_comments_original_submission.pdf]

Author's Response To Reviewer Comments

Re: GIGA-D-24-00434, "A Novel Dataset for Nuclei and Tissue Segmentation in Melanoma with baseline nuclei segmentation and tissue segmentation benchmarks"

Dear, Nicole Nogoy, Ph.D,

Thank you for your evaluation of our manuscript "A Novel Dataset for Nuclei and Tissue Segmentation in Melanoma with baseline nuclei segmentation and tissue segmentation benchmarks". We highly appreciate the thoughtful feedback and constructive suggestions from the reviewers and editors, which have helped us to revise our manuscript.

We have addressed each comment from the reviewers and editors point-by-point below. We hope that the revisions will meet the expectations of the journal and contribute positively to the quality of our data and its description.

We look forward to your response and thank you for the opportunity to resubmit our manuscript.

Yours sincerely, on behalf of all co-authors,

Mark Schuiveling, MD MSc
Dr. Mitko Veta, PhD

Medical Image Analysis,
Department of Biomedical Engineering,
Eindhoven University of Technology,
Eindhoven, the Netherlands

Comments from the Reviewers
Reviewer #1:
The paper presents a dataset for nuclei and tissue segmentation in melanoma with about 155 primary and 155 metastatic cases from a single referral institution. The authors use Hover-NeXt model to demonstrate the performance of lymphocyte detection. The post processing further improves the detection results. The lymphocyte detection is used to estimate TILs scores as there is evidence that TILs are associated with reduced rates in primary melanoma. The authors have used original pipelines for training HoverNet and HoVerNext so the comparison is fair.

1. For metrics however, I would suggest to add additional metrics for a fair comparison for segmentation. Is DICE loss used in training the network? If yes then the comparison is not fair and additional metrics should be used such as pixel accuracy, F1-score, Hausdorff distance and panoptic quality to estimate the performance of segmentation and classification for both tissue and nuclei segmentation. DICE loss does not give an overall picture.

We thank the reviewer for their assessment of our manuscript and valuable suggestions. When training nnU-Net and Mask2Former no changes were made to the original code. This means that for nnU-Net training a complex loss combining the sum of cross-entropy and Dice loss is used. Regarding Mask2Former, a complex loss based on dice loss, cross-entropy and focal loss is used.

Regarding the metrics for tissue segmentation:
We deliberately chose DICE score as a metric for tissue segmentation due as it is a commonly used (if not the most common) metric for medical image segmentation tasks [1]. As both our segmentation models use Dice loss in their loss calculation, we still believe it to be fair. In addition, due its common use it makes our

results easily interpretable. Haussdorf distance is not a suitable metric whereas it does not take holes in segmentation into account.

Regarding the metrics for nuclei segmentation:
We used F1 score based on centroid distance for nuclei segmentation, as the small size of nuclei makes metrics such as panoptic quality and aggregated Jaccard index unsuitable due their dependence on Intersection over Union. Foucart et al. (2023) demonstrated that IoU based metrics such as panoptic quality are overly sensitive for small annotations, leading to substantial metric changes from minor segmentation differences [2]. This results in a higher chance in misclassifications for small objects such as lymphocytes. Therefore, we used classification based on centroid distance as used by the Ocelot challenge, which follow the Metrics-Reloaded guidelines as stated by Maier-Hein et al [1].

2. Combining the tissue and nuclei segmentation/classification improves the results as expected. One of the major issues I had while reading the paper was to understand what data has been made publicly available. In data availability section, it says training dataset has been made available but then it says 104 annotated ROIs are hidden and will remain so for another five years. I am not clear if the hidden ROIs include the training dataset? Or the hidden ROIs are the test set? If this is the test set then it makes sense.

We understand the confusion this generates. We therefore adjusted the manuscript with the following with more clearly describes what part of the dataset is publicly available. In addition, it more clearly describes the dataset division which was also confusing as stated by the second reviewer.
“ From the total dataset of 310 ROIs, a training set consisting of 103 primary and 103 metastatic ROI has been made publicly available [3]. The remaining 104 ROIs are not publicly available as they are used in the PUMA challenge. In this challenge, 10 ROIs are used a preliminary test set for testing of functioning of models. The remaining 94 samples are used as an independent test set for final metric calculations. To be able to compare results from the PUMA challenge to our paper results in this paper are shown for this independent test set.

The tissue distribution for the training, preliminary and final test set is visualized in Figure 3. In primary samples, more tumoral stroma is present compared to metastatic samples. The epidermis and necrotic area are underrepresented in both datasets.

Figure 3. Distribution of tissue class area in the PUMA dataset.
‟

3. I have also looked at the grand challenge website. I couldn't find a link between the paper "data availability" section and the challenge website. I would suggest to make it clear in the data availability. You can specify training data set available at ..., validation available at ... and test can be run on grand challenge. If that's the case. The other point as I mentioned above is related to the metrics used for evaluation they should be broad.

We agree with the reviewer that a link to the PUMA challenge needs to be added to the data availability statement. We therefore added this to data availability statement which resulted in the following:

“The PUMA training dataset is available on Zenodo at: https://zenodo.org/records/10940194. Joining the PUMA challenge is possible through the grand challenge platform at https://puma.grand-challenge.org/. The preliminary and final test set will stay hidden until October 10th 2029 due the PUMA challenge still being open for submissions until then. Readers can request earlier access to the hidden dataset for educational or collaborative purposes by contacting the corresponding author via email.”

Reviewer #2: *
General Comments: The paper is well-written and easy to follow.
We thank the reviewer for their kind words.

1. * Current Text: "We will use this dataset to organize the PUMA challenge in which the goal is to further improve model performance." --> Recommendation: Remove "will" as the PUMA challenge is already in progress.

We agree with the reviewer that this is no longer the correct statement. The abstract conclusion has been changed into the following:

"The PUMA dataset is the first melanoma specific dataset that can be used to develop melanoma-specific nuclei and tissue segmentation models. These models can, in turn, be used for prognostic and predictive biomarker development. Incorporating tissue and nuclei segmentation is a step towards improved deep learning nuclei segmentation performance. To support the development of these models, this dataset is used in the PUMA challenge. "

2. * As this dataset is currently used for the PUMA challenge, further details about the challenge, including a link to the challenge website, should be added to the text.

We agree that this should be added. As stated in the response to reviewer 1 a link to the challenge has been added to the data availability statement. In addition, we added a link to the data description:
"The PUMA dataset consists of regions of interest (ROIs) with nuclei and tissue annotations. ROIs originate from H&E stained histological slides of melanoma specimens. The dataset's goal is to facilitate the development of deep learning models capable of segmenting nuclei and tissue. To stimulate the use of the dataset and create novel deep learning models, the dataset will also be used for a medical image analysis challenge hosted on the grand-challenge.org platform (accessible through https://puma.grand-challenge.org/). The models created with the dataset and the PUMA challenge, in turn, can be used for prognostic biomarker generation in melanoma treatment. "

3. * Figure 2 shows the distribution of nuclei and sampled tissue in the PUMA dataset for primary melanoma regions (half of the dataset). However, what about the distribution for metastatic ROIs (the other half)?

Figure 2 shows the distribution for the primary samples (in bold). The other samples are all metastatic samples. Due to the confusion we updated the figure caption:

"Figure 2. Distribution of nuclei and sampled tissue in the PUMA dataset for primary melanoma regions of interest and metastatic melanoma regions of interest stratified according to metastasis location. The primary samples are depicted as the last row of each graph. "

4. * Current Text: "We used 5-fold cross-validation while training and report the results of inference on the 94 ROIs of the final independent test set used in the PUMA challenge." Earlier in the text, it states: "The test set consists of 50,490 nuclei in 52 primary and 52 metastatic ROIs" (104 images). Upon careful reading, I understood that 94 images are used as the final test set, while 10 are used for validation. To prevent confusion, this information should be stated clearly and as early as possible in the text, such as on page 6.

We agree that this is not clear in the current manuscript. We therefore adjusted this on page 6 to make it

more clear. The changes made are visible in the response of question 2 from reviewer 1.

5. * Since this dataset is specifically designed for histological image analysis of melanoma, other publicly available nuclei instance segmentation (or classification) datasets, such as NuInsSeg or CryoNuSeg, which include H&E-stained skin images, should be mentioned. These datasets can be used for tasks like pre-training to enhance the performance of DL-based approaches on the PUMA dataset.
https://www.sciencedirect.com/science/article/pii/S0010482521001438
https://www.nature.com/articles/s41597-024-03117-2

We agree that these datasets, which also include skin samples, should also be mentioned. Next to this dataset, we also added a section about the SegPath dataset as this also contains a melanoma sample. The following has been added to the introduction of the manuscript:
" As for datasets which can be used to train deep learning models usable in melanoma, the NulnsSeg [4] and CryoNuSeg [5] datasets also partly consist of skin samples. However these datasets do not have separate classes for nuclei such as tumor or lymphocyte rendering them less useful for creating deep learning nuclei classification algorithms. In contract, the SegPath dataset does classify nuclei into distinct groups using a ground truth based on immunofluorescence staining. However, it contains only one melanoma sample with CD3/CD20 annotations [6]. "

6. * The hyperparameters of the used nn-UNet should be added to the text. Has any ensembling been employed?

nnU-Net is a segmentation framework that automatically adapts to the characteristics of a given dataset. By analyzing the provided training data, it configures a U-Net-based segmentation pipeline tailored to the dataset[7]. We utilized nnU-Net as-is, without modifying its default hyperparameters, which is why no hyperparameters were explicitly added to the Methods section.
In our Methods, we state:
"For training of nnU-Net the same fivefold cross validation was used to create an ensemble model."
This follows the standard practice in nnU-Net's training code and reflects the framework's default ensembling strategy.

7. * The nn-UNet is primarily designed for 3D datasets. What motivated its use for 2D image segmentation in this work rather than opting for more advanced architectures specifically designed for 2D segmentation, such as those based on vision transformers?

nnU-Net is a versatile framework capable of handling both 2D and 3D datasets across various imaging modalities [7]. It has been successfully applied to 2D segmentation tasks in histopathology with high performance [8].
In addition, we explored a vision transformer based architecture with a Mask2Former model with the backbone replaced by the UNI pathology foundation model [7,9,10]. Nn-UNet, however, had better performance as it tunes automatically all its hyperparameters during the training which for small datasets such as ours gives better performance.

8. * The results reported in Table 2 are based on the F1 score. Given that the challenge name implies panoptic segmentation of nuclei, why were common evaluation metrics for nuclei instance segmentation and classification tasks, such as Average Panoptic Quality or Average AJI, not used? Is the F1 score sensitive to the ability of the model to separate overlapping nuclei?

We used F1 score based on centroid distance for nuclei segmentation, as the small size of nuclei makes metrics such as panoptic quality and aggregated Jaccard index unsuitable due their dependence on Intersection over Union. Foucart et al. (2023) demonstrated that IoU based metrics such as panoptic quality are overly sensitive for small annotations, leading to substantial metric changes from minor segmentation differences [2]. This results in a higher chance in misclassifications for small objects such as lymphocytes. Therefore, we used classification based on centroid distance as used by the Ocelot challenge, which follow the Metrics-Reloaded guidelines as stated by Maier-Hein et al [1].
This metric is also able to separate overlapping nuclei due the assessment of each individual nuclei from a .geoJSON (or JSON in the PUMA challenge) with polygon coordinates.

9. * A challenge participant has noted a mismatch between the annotation and image file training_set_metastatic_roi_103 ((https://grand-challenge.org/forums/forum/panoptic-segmentation-of-nuclei-and-tissue-in-advanced-melanoma-737/topic/potential-annotation-issue-in-file-training_set_metastatic_roi_103-2877/). Please comment on this issue and confirm that no such problems exist in your validation and test datasets.

We regret that this has happened. We deleted the sample from the dataset to level the playing field for the PUMA challenge. After the challenge deadline (1st of March 2025) has been passed we will upload the correct image. As an additional check, we added an overlay of the preliminary and final test set to the rebuttal to show that there are no more errors in the dataset. This is downloadable from surfdrive with the following link: https://surfdrive.surf.nl/files/index.php/s/PHjbEjbokfNx244 (password gigascience).

10. * While annotations are currently provided in .geojson format, it would be beneficial to include them in other conventional formats, such as .png files or as overlays on the images. This could be achieved either by including additional files in the dataset or by adding a Qupath script in the GitHub repository to save the annotations for all images in various formats.

We added qupath scripts to the zenodo repository with the weights which give users the possibility to export the annotations as masks. A link to this has been added to the availability of source code and requirements section:
" Qupath scripts to import .geoJSONs for viewing and exporting annotations as masks can be found here: https://zenodo.org/records/13897135."

11. * From my understanding, the context ROIs of 5120×5120 pixels are provided only for the training set. If a model relies on this information during training, similar data would also be needed for the validation and test sets. Are context ROIs provided for validation and test data as well? Please clarify.

We understand that this might seem strange. Our idea was that these context regions of interest could be used to generate more data through inference on these samples. Giving the context regions of interest for the validation and test set would give participants the opportunity to reconstruct the ground truth from these data sets. This would not be in line with the goal of the challenge which is generating the best possible deep learning model for inference on previously unseen data.
However, after the 5 year embargo this data will be released together with the annotations and regions of interest of the test set.

12. * On page 26, the link https://pumachallenge.com/ is not working. Please update or replace it with a functional link.

We replaced the link with a link to the PUMA grand challenge website. (Project home page:

https://puma.grand-challenge.org/)

13. * "This research was funded by an unrestricted grant of Stichting Hanarth Fonds, The Netherlands.." --> Please remove one of the dots at the end

The dot was removed.

References:

1. Maier-Hein L, Reinke A, Godau P, Tizabi MD, Buettner F, Christodoulou E, et al.. Metrics reloaded: recommendations for image analysis validation. Nat Methods. Nature Publishing Group; 2024; doi: 10.1038/s41592-023-02151-z.
2. Foucart A, Debeir O, Decaestecker C. Panoptic quality should be avoided as a metric for assessing cell nuclei segmentation and classification in digital pathology. Sci Rep. Nature Publishing Group; 2023; doi: 10.1038/s41598-023-35605-7.
3. Schuiveling M. Melanoma Histopathology Dataset with Tissue and Nuclei Annotations. Zenodo; 2024; doi: 10.5281/zenodo.10940193.
4. Mahbod A, Polak C, Feldmann K, Khan R, Gelles K, Dorffner G, et al.. NuInsSeg: A fully annotated dataset for nuclei instance segmentation in H&E-stained histological images. Sci Data. Nature Publishing Group; 2024; doi: 10.1038/s41597-024-03117-2.
5. Mahbod A, Schaefer G, Bancher B, Löw C, Dorffner G, Ecker R, et al.. CryoNuSeg: A dataset for nuclei instance segmentation of cryosectioned H&E-stained histological images. Computers in Biology and Medicine. 2021; doi: 10.1016/j.compbiomed.2021.104349.
6. Komura D, Onoyama T, Shinbo K, Odaka H, Hayakawa M, Ochi M, et al.. Restaining-based annotation for cancer histology segmentation to overcome annotation-related limitations among pathologists. Patterns. 2023; doi: 10.1016/j.patter.2023.100688.
7. Isensee F, Jaeger PF, Kohl SAA, Petersen J, Maier-Hein KH. nnU-Net: a self-configuring method for deep learning-based biomedical image segmentation. Nat Methods. Nature Publishing Group; 2021; doi: 10.1038/s41592-020-01008-z.
8. Spronck J, Gelton T, Eekelen L van, Bogaerts J, Tessier L, Rijthoven M van, et al.. nnUNet meets pathology: Bridging the gap for application to whole slide images and computational biomarkers. Medical Imaging with Deep Learning. PMLR; p. 1859–74.
9. : Towards a general-purpose foundation model for computational pathology | Nature Medicine. https://www.nature.com/articles/s41591-024-02857-3 Accessed 2024 Sep 23.
10. Cheng B, Misra I, Schwing AG, Kirillov A, Girdhar R. Masked-attention Mask Transformer for Universal Image Segmentation. arXiv;
