# Supplementary material for: A novel dataset for nuclei and tissue segmentation in melanoma with baseline nuclei segmentation and tissue segmentation benchmarks
Source: Gigascience. 2025 Feb 19;14:giaf011. doi: 10.1093/gigascience/giaf011 (PMC11837757; doi:10.1093/gigascience/giaf011)
Supplement: giaf011_Response_to_Reviewer_Comments_Revision_1 [file giaf011_response_to_reviewer_comments_revision_1.pdf]

Author's Response To Reviewer Comments

Dear Dr. Nogoy,

Thank you for your email and for providing the detailed feedback on our manuscript, "A novel dataset for nuclei and tissue segmentation in melanoma with baseline nuclei segmentation and tissue segmentation benchmarks" (GIGA-D-24-00434R1).

I am grateful for the comments on how to properly cite software and related resources as this will properly guide readers to where these resources can be found. As a medical doctor, this was something I did not do before, and I apologize for not formatting these citations correctly in the original submission. Following your instructions, I have updated the citations and references to meet the journal's standards.

I hope these changes bring the manuscript closer to being suitable for publication. However, if any further revisions are needed, please do not hesitate to contact me.

Kind regards,
Mark Schuiveling
PhD candidate medical oncology
UMC Utrecht, the Netherlands
