# Supplementary material for: A novel dataset for nuclei and tissue segmentation in melanoma with baseline nuclei segmentation and tissue segmentation benchmarks
Source: Gigascience. 2025 Feb 19;14:giaf011. doi: 10.1093/gigascience/giaf011 (PMC11837757; doi:10.1093/gigascience/giaf011)
Supplement: giaf011_Reviewer_1_Report_Original_Submission — Shan Raza -- 11/20/2024 [file giaf011_reviewer_1_report_original_submission.pdf]

Reviewer Report

Title: A novel dataset for nuclei and tissue segmentation in melanoma with baseline nuclei segmentation and tissue segmentation benchmarks

Version: Original Submission **Date:** 11/20/2024

Reviewer name: Shan Raza

Reviewer Comments to Author:

The paper presents a dataset for nuclei and tissue segmentation in melanoma with about 155 primary and 155 metastatic cases from a single referral institution. The authors use Hover-NeXt model to demonstrate the performance of lymphocyte detection. The post processing further improves the detection results. The lymphocyte detection is used to estimate TILs scores as there is evidence that TILs are associated with reduced rates in primary melanoma. The authors have used original pipelines for training HoverNet and HoVerNext so the comparison is fair. For metrics however, I would suggest to add additional metrics for a fair comparison for segmentation. Is DICE loss used in training the network? If yes then the comparison is not fair and additional metrics should be used such as pixel accuracy, F1-score, Hausdorff distance and panoptic quality to estimate the performance of segmentation and classification for both tissue and nuclei segmentation. DICE loss does not give an overall picture. Combining the tissue and nuclei segmentation/classification improves the results as expected. One of the major issues I had while reading the paper was to understand what data has been made publicly available. In data availability section, it says training dataset has been made available but then it says 104 annotated ROIs are hidden and will remain so for another five years. I am not clear if the hidden ROIs include the training dataset? Or the hidden ROIs are the test set? If this is the test set then it makes sense. I have also looked at the grand challenge website. I couldn't find a link between the paper "data availability" section and the challenge website. I would suggest to make it clear in the data availability. You can specify training data set available at ..., validation available at ... and test can be run on grand challenge. If that's the case. The other point as I mentioned above is related to the metrics used for evaluation they should be broad.

Methods

Are the methods appropriate to the aims of the study, are they well described, and are necessary controls included? Choose an item.

Conclusions

Are the conclusions adequately supported by the data shown? Choose an item.

Reporting Standards

Does the manuscript adhere to the journal's guidelines on [minimum standards of reporting?]() Choose an item.

Choose an item.

Statistics

Are you able to assess all statistics in the manuscript, including the appropriateness of statistical tests used? Choose an item.

Quality of Written English

Please indicate the quality of language in the manuscript: Choose an item.

Declaration of Competing Interests

Please complete a declaration of competing interests, considering the following questions:

- Have you in the past five years received reimbursements, fees, funding, or salary from an organisation that may in any way gain or lose financially from the publication of this manuscript, either now or in the future?
- Do you hold any stocks or shares in an organisation that may in any way gain or lose financially from the publication of this manuscript, either now or in the future?
- Do you hold or are you currently applying for any patents relating to the content of the manuscript?
- Have you received reimbursements, fees, funding, or salary from an organization that holds or has applied for patents relating to the content of the manuscript?
- Do you have any other financial competing interests?
- Do you have any non-financial competing interests in relation to this paper?

If you can answer no to all of the above, write 'I declare that I have no competing interests' below. If your reply is yes to any, please give details below.

I declare that I have no competing interests.

I agree to the open peer review policy of the journal. I understand that my name will be included on my report to the authors and, if the manuscript is accepted for publication, my named report including any attachments I upload will be posted on the website along with the authors' responses. I agree for my report to be made available under an Open Access Creative Commons CC-BY license (http://creativecommons.org/licenses/by/4.0/). I understand that any comments which I do not wish to be included in my named report can be included as confidential comments to the editors, which will not be published.

Choose an item.

To further support our reviewers, we have joined with Publons, where you can gain additional credit to further highlight your hard work (see: https://publons.com/journal/530/gigascience). On publication of this paper, your review will be automatically added to Publons, you can then choose whether or not to claim your Publons credit. I understand this statement.

Yes Choose an item.
