# Supplementary material for: A novel dataset for nuclei and tissue segmentation in melanoma with baseline nuclei segmentation and tissue segmentation benchmarks
Source: Gigascience. 2025 Feb 19;14:giaf011. doi: 10.1093/gigascience/giaf011 (PMC11837757; doi:10.1093/gigascience/giaf011)
Supplement: giaf011_Reviewer_2_Report_Original_Submission — Amirreza Mahbod -- 11/21/2024 [file giaf011_reviewer_2_report_original_submission.pdf]

Reviewer Report

Title: A novel dataset for nuclei and tissue segmentation in melanoma with baseline nuclei segmentation and tissue segmentation benchmarks

Version: Original Submission **Date:** 11/21/2024

Reviewer name: Amirreza Mahbod

Reviewer Comments to Author:

* General Comments: The paper is well-written and easy to follow.
* Current Text: "We will use this dataset to organize the PUMA challenge in which the goal is to further improve model performance." --> Recommendation: Remove "will" as the PUMA challenge is already in progress.
* As this dataset is currently used for the PUMA challenge, further details about the challenge, including a link to the challenge website, should be added to the text.
* Figure 2 shows the distribution of nuclei and sampled tissue in the PUMA dataset for primary melanoma regions (half of the dataset). However, what about the distribution for metastatic ROIs (the other half)?
* Current Text: "We used 5-fold cross-validation while training and report the results of inference on the 94 ROIs of the final independent test set used in the PUMA challenge." Earlier in the text, it states: "The test set consists of 50,490 nuclei in 52 primary and 52 metastatic ROIs" (104 images). Upon careful reading, I understood that 94 images are used as the final test set, while 10 are used for validation. To prevent confusion, this information should be stated clearly and as early as possible in the text, such as on page 6.
* Since this dataset is specifically designed for histological image analysis of melanoma, other publicly available nuclei instance segmentation (or classification) datasets, such as NuInsSeg or CryoNuSeg, which include H&E-stained skin images, should be mentioned. These datasets can be used for tasks like pre-training to enhance the performance of DL-based approaches on the PUMA dataset.
https://www.sciencedirect.com/science/article/pii/S0010482521001438
https://www.nature.com/articles/s41597-024-03117-2
* The hyperparameters of the used nn-UNet should be added to the text. Has any ensembling been employed?
* The nn-UNet is primarily designed for 3D datasets. What motivated its use for 2D image segmentation in this work rather than opting for more advanced architectures specifically designed for 2D segmentation, such as those based on vision transformers?
* The results reported in Table 2 are based on the F1 score. Given that the challenge name implies panoptic segmentation of nuclei, why were common evaluation metrics for nuclei instance segmentation and classification tasks, such as Average Panoptic Quality or Average AJI, not used? Is the F1 score sensitive to the ability of the model to separate overlapping nuclei?
* A challenge participant has noted a mismatch between the annotation and image file training_set_metastatic_roi_103 ((https://grand-challenge.org/forums/forum/panoptic-segmentation-

of-nuclei-and-tissue-in-advanced-melanoma-737/topic/potential-annotation-issue-in-file-training_set_metastatic_roi_103-2877/). Please comment on this issue and confirm that no such problems exist in your validation and test datasets.
* While annotations are currently provided in .geojson format, it would be beneficial to include them in other conventional formats, such as .png files or as overlays on the images. This could be achieved either by including additional files in the dataset or by adding a Qupath script in the GitHub repository to save the annotations for all images in various formats.
* From my understanding, the context ROIs of 5120Ã—5120 pixels are provided only for the training set. If a model relies on this information during training, similar data would also be needed for the validation and test sets. Are context ROIs provided for validation and test data as well? Please clarify.
* On page 26, the link https://pumachallenge.com/ is not working. Please update or replace it with a functional link.
* "This research was funded by an unrestricted grant of Stichting Hanarth Fonds, The Netherlands.." --> Please remove one of the dots at the end

Methods

Are the methods appropriate to the aims of the study, are they well described, and are necessary controls included? Choose an item.

Conclusions

Are the conclusions adequately supported by the data shown? Choose an item.

Reporting Standards

Does the manuscript adhere to the journal's guidelines on [minimum standards of reporting?](minimum standards of reporting) Choose an item.

Choose an item.

Statistics

Are you able to assess all statistics in the manuscript, including the appropriateness of statistical tests used? Choose an item.

Quality of Written English

Please indicate the quality of language in the manuscript: Choose an item.

Declaration of Competing Interests

Please complete a declaration of competing interests, considering the following questions:

- Have you in the past five years received reimbursements, fees, funding, or salary from an organisation that may in any way gain or lose financially from the publication of this manuscript, either now or in the future?
- Do you hold any stocks or shares in an organisation that may in any way gain or lose financially from the publication of this manuscript, either now or in the future?
- Do you hold or are you currently applying for any patents relating to the content of the manuscript?
- Have you received reimbursements, fees, funding, or salary from an organization that holds or has applied for patents relating to the content of the manuscript?
- Do you have any other financial competing interests?
- Do you have any non-financial competing interests in relation to this paper?

If you can answer no to all of the above, write 'I declare that I have no competing interests' below. If your reply is yes to any, please give details below.

I declare that I have no competing interests. I plan to participate in the PUMA challenge but have no financial or scientific relationship with the challenge organizers.

I agree to the open peer review policy of the journal. I understand that my name will be included on my report to the authors and, if the manuscript is accepted for publication, my named report including any attachments I upload will be posted on the website along with the authors' responses. I agree for my report to be made available under an Open Access Creative Commons CC-BY license (http://creativecommons.org/licenses/by/4.0/). I understand that any comments which I do not wish to be included in my named report can be included as confidential comments to the editors, which will not be published.

Choose an item.

To further support our reviewers, we have joined with Publons, where you can gain additional credit to further highlight your hard work (see: https://publons.com/journal/530/gigascience). On publication of this paper, your review will be automatically added to Publons, you can then choose whether or not to claim your Publons credit. I understand this statement.

Yes Choose an item.
